# Safety Profile of SGLT-2 Inhibitors in Older Adults: A Systematic Review and Network Meta-Analysis

**DOI:** 10.3390/medsci14010153

**Published:** 2026-03-20

**Authors:** Kannan Sridharan, Gowri Sivaramakrishnan

**Affiliations:** 1 Department of Pharmacology & Therapeutics, College of Medicine & Health Sciences, Arabian Gulf University, Manama P.O. Box 26671, Bahrain; 2 Bahrain Defence Force Royal Medical Services, Riffa P.O. Box 28743, Bahrain; gowri.sivaramakrishnan@gmail.com

**Keywords:** SGLT2i, empagliflozin, dapagliflozin, canagliflozin, sotagliflozin

## Abstract

Background: Sodium–glucose cotransporter-2 inhibitors (SGLT2i) are widely used in older adults for diabetes, heart failure, and kidney disease. This is the first network meta-analysis focusing on the effects of SGLT2i in older adults. Methods: Databases were searched for randomized clinical trials comparing SGLT2i against non-SGLT2i controls or other SGLT2i in relevant populations. Key safety outcomes included acute renal failure (ARF), genital infections, volume depletion, mortality, and serious adverse events (SAEs). Pooled odds ratios (OR) with 95% confidence intervals (CI) were generated using random-effects models for direct and mixed treatment comparisons. Results: From 97 included trials in the meta-analysis, SGLT2i versus non-SGLT2i were associated with reduced risks of ARF (OR 0.86, 95% CI 0.79–0.94), mortality (OR 0.84, 0.75–0.93), and SAEs (OR 0.84, 0.78–0.89), but increased risks of genital infections (OR 3.32, 2.68–4.12) and volume depletion (OR 1.18, 1.09–1.27). The risk of genital infections was observed more frequently with higher doses (high-dose OR 4.73 vs. low-dose OR 2.90) and escalated sharply with age (≥75 years OR 9.29, 3.13–27.6). The mortality benefit was strongest in adults ≥75 years (OR 0.58, 0.38–0.88). Intra-class analysis revealed distinct safety profiles; for instance, empagliflozin reduced the ARF risk, while sotagliflozin increased the volume depletion risk. Bootstrap and trial sequential analyses confirmed the results’ robustness. Grading of Recommendations Assessment, Development, and Evaluation assessment indicated moderate certainty of evidence. Conclusions: In older adults, SGLT2i maintain a favorable benefit–risk profile, with significant reductions in mortality and SAEs, though risks of genital infections and volume depletion require vigilance. The risk of genital infections exhibits a strong dose–response relationship and increases markedly in the oldest adults, while the mortality benefit appears to be most pronounced in those aged 75 years and older. This study provides actionable insights for personalized therapy in geriatric care.

## 1. Introduction

Sodium–glucose cotransporter-2 inhibitors (SGLT2i) have rapidly gained prominence for their FDA-approved indications in type 2 diabetes (T2D), both as monotherapy and in combination with other agents, and more recently for heart failure (HF) with reduced (HFrEF) and preserved (HFpEF) ejection fraction [1,2]. In the United States, the use of SGLT2i has surged, increasing from 0.4% to 5.7% among patients with T2D, from 0.3% to 18.6% in HFrEF, and from 0.5% to 9.9% in HFpEF [3,4]. An estimated one-third of adults over the age of 65 meet the diagnostic criteria for T2D, while the condition is diagnosed in approximately 90% of this population [5]. Similarly, the worldwide prevalence of HF in older adults is estimated to range from 4.7% to 13.3% [6].

Physiological aging involves significant changes, including alterations in renal tubular function, electrolyte balance, and the sensitivity of critical organs such as the heart, brain, liver, and kidneys [7]. Despite these changes, several clinical trials have demonstrated that SGLT2i retain their pharmacodynamic effects, such as glycemic control and reduction in HbA1c, in older adults [8]. Concerning the safety profile of SGLT2i, clinical trials and subsequent meta-analyses in general populations have established risks including urinary tract infections (UTI), genital infections, ketoacidosis (KA), fractures, and lower limb amputations [9,10,11]. However, a comprehensive, quantitative synthesis of the safety data for SGLT2i, specifically in older adults, remains lacking. The sole qualitative systematic assessment, conducted by Scheen et al., did not employ meta-analytic tools [12]. This absence of a quantitative synthesis is a clinically significant gap, as it precludes the ability to compare safety outcomes across different age subgroups (e.g., young–old vs. old–old) or to detect potential differences between individual SGLT2i drugs, which is essential for personalized prescribing in this heterogeneous population [12]. Furthermore, it is unclear whether intra-class differences exist among individual SGLT2i regarding the risk of various adverse events in this subpopulation. A primary constraint for conducting a conventional meta-analysis in older adults is the absence of direct head-to-head clinical trials.

A network meta-analysis (NMA) is a sophisticated methodological tool that allows for the indirect comparison of multiple interventions through a common comparator, even in the absence of direct head-to-head trials [13]. Given the pressing need for a quantitative safety evaluation that accounts for age and dose, we undertook this study to systematically analyze and quantify the risks of key safety outcomes associated with SGLT2i use in older adults, aiming to provide evidence-based clinical recommendations.

## 2. Methods

### 2.1. Search Strategy

This study was carried out as an ancillary meta-analysis of the systematic review assessing the overall safety profile of SGLT2i, for which the protocol has been registered [14]. The following databases were searched for appropriate articles: PubMed, Cochrane Central, and Google Scholar. The search strategy used in the present study is summarized in the Electronic Supplementary Table S1, with the latest search carried out on 30 November 2025. No restrictions were placed on either the publication year or language. Conference proceedings were not included. A search of published articles was also carried out to identify other publications that were suitable for inclusion in this review. The present network meta-analysis is reported in compliance with the Preferred Reporting Items for Systematic Reviews and Meta-Analyses (PRISMA) statement [15].

### 2.2. Eligibility Criteria

We included only randomized clinical trials (RCTs) that met the following criteria.

Population: Adults with disorders such as diabetes mellitus, heart failure and chronic kidney disease, for which SGLT2i was administered. Following the initial search, data were extracted and synthesized only from studies that were either exclusively carried out in older adults or where the average age reported for participants was 65 years and above, or from trials that specifically reported outcomes for this subgroup.

Intervention: Any of the SGLT2i including Bexagliflozin, Empagliflozin, Dapagliflozin, Canagliflozin, Enavogliflozin, Ertugliflozin, Henagliflozin, Ipragliflozin, Licogliflozin, Luseogliflozin, Remogliflozin, Sotagliflozin, and Tofogliflozin were considered.

Control: Non-SGLT2i (placebo/standard of care/any active drug) or any of the above listed SGLT2i.

Outcome: All the reported safety outcomes were categorized as follows: acute renal failure (ARF), amputation, constipation, diarrhea, KA, fracture, genital infections, hepatic dysfunction, hyperkalemia, hypoglycemia, hypotension, malignancies, myocardial infarction, mortality, polyuria, serious adverse events (SAE), stroke, UTI, and volume depletion. Additionally, we also considered the discontinuation of therapy due to adverse events as an outcome.

### 2.3. Study Procedure

An independent search was carried out by two authors who obtained the following details: trial identification, year, diagnosis of study participants [type 2 diabetes (T2D), type 1 diabetes (T1D), heart failure (HF), acute coronary syndrome (ACS), chronic kidney disease (CKD), Coronavirus disease 2019 (COVID-19), pulmonary arterial hypertension (PAH), implantable cardioverter defibrillator or cardiac resynchronization therapy defibrillator (ICD/CRT-D), atrial high-rate episodes (AHRE), cardiorenal syndrome (CRS)], demographic characteristics of the study participants [age, gender distribution, baseline HbA1c, baseline estimated glomerular filtration rate (eGFR), body mass index (BMI), and duration of diabetes], drug-related details (name, dose, frequency, and duration) and outcomes. Data extraction was performed independently by two reviewers. Any discrepancies in the extracted data were resolved through discussion between the two reviewers until a consensus was reached. Conflicts in data retrieval were resolved through discussion until a consensus was attained. The risk of bias of the included studies was assessed using domains of the Cochrane risk of bias tool [16], including generation of random sequence, concealment of allocation, blinding of participants, study personnel, outcome assessment, incomplete outcome reporting, and selective reporting of outcomes. Publication bias for SGLT2i versus non-SGLT2i was assessed using a funnel plot and Egger’s regression analysis [17]. A random-effects model was used to generate direct, indirect, and mixed treatment comparison pooled estimates. Data from head-to-head clinical trials provided direct comparison pooled estimates while indirect pooled estimates were obtained from clinical trials using common comparators. The direct and indirect comparison pooled estimates were used to generate mixed comparison pooled estimates. Direct comparison pooled estimates were generated for the outcomes between SGLT2i and non-SGLT2i. Mixed treatment comparison pooled estimates were obtained for several categories (Table 1). The classification of dose categories for each SGLT2i included in this study is summarized in Electronic Supplementary Table S2. Odds ratios [95% confidence intervals] (OR 95% CI) were used as effect estimates. H statistics were applied to evaluate inconsistency between direct and indirect pooled estimates, classified as follows: mild (<3), modest (3–6), and large (>6) [18]. For direct pairwise comparisons, I^2^ values were used for quantifying heterogeneity as follows: low (<25%), moderated (25 ≤ 50%), high (>50 ≤ 75%), and substantial (>75%) [19]. To address potential heterogeneity in the definition and reporting of safety outcomes across the included trials, we accepted the outcome definitions as reported by the original study authors and did not attempt to reclassify events post hoc. This is an inherent limitation of study-level meta-analyses, as we could not adjust for patient-level confounders such as frailty, functional status, or polypharmacy, which may influence the risk of adverse events in older adults. NMA relies on two fundamental assumptions: transitivity and consistency. Transitivity, the assumption that the distribution of effect modifiers is similar across comparisons, was ensured, as the population was homogenous in terms of being older adults and indications. Consistency, the agreement between direct and indirect evidence within the network, was formally assessed using the H statistic for inconsistency, which was <3 for all outcomes, indicating mild inconsistency and supporting the validity of the mixed treatment comparisons. These assessments provide confidence that the assumptions underlying our network meta-analysis were adequately met. The inclusion of studies that directly compare one SGLT2i against another is essential for addressing the primary objectives of this NMA. First, including head-to-head trials enables the construction of a fully connected evidence network, allowing for indirect comparisons between all interventions, even in the absence of direct trials for every possible pair. This maximizes the statistical power and precision of our mixed treatment comparisons. Second, a key objective of our study was to investigate potential intra-class differences in safety profiles among individual SGLT2i drugs in older adults. Head-to-head trials provide the most direct and reliable evidence for such comparisons. By including them, our NMA generates pooled estimates for intra-class comparisons that are informed by both direct trial data (where available) and indirect data through common comparators, providing the most comprehensive and reliable answer possible. Finally, head-to-head trials of SGLT2i typically enroll patient populations and employ study designs that are comparable to placebo-controlled trials of the same class, thereby satisfying the transitivity assumption required for valid NMA. MetaXL© was used to estimate mixed comparison pooled estimates. A sensitivity analysis was carried out using the leave-one-out meta-analysis method, where data from one study was excluded at a time to assess the impact on pooled estimates. Bootstrapping of the pooled estimates for 1000 iterations was carried out to confirm the robustness of the pooled estimates. The grading of pooled estimates was carried out using the GRADE working group approach [20]. Meta-regression for risk of genital infections was carried out separately, using various covariates (Table 1). Meta-regression analysis was only carried out for the following outcomes, which were associated with significant pooled estimates, as the other outcomes were constrained by sample size: SAE, volume depletion, mortality and genital infections. A random-effects meta-regression analysis was performed to examine the association between study-level covariates and treatment effects. Univariable analyses were conducted for each covariate. A multivariable meta-regression model was subsequently fitted. All analyses used restricted maximum likelihood estimation for between-study variance, and results are presented as OR with 95% CI. *p*-values of ≤0.05 were considered significant for pooled estimates of covariates in meta-regression.

## 3. Results

### 3.1. Search Results

**Figure 1:**
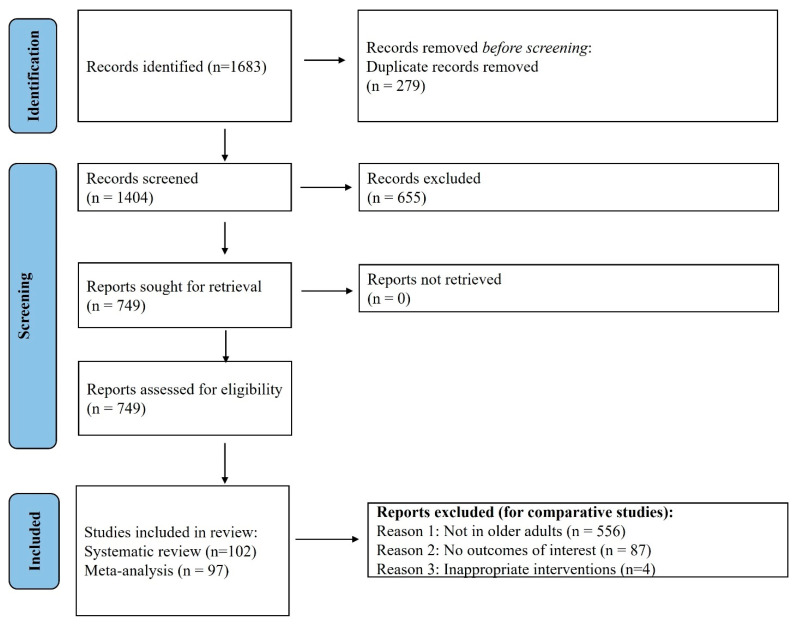
PRISMA flow diagram. A total of 1683 studies were obtained with the search strategy, of which 102 were included in the systematic review.

A total of 102 studies were included in this review from the total of 1683 obtained with the search strategy (Figure 1). Of these, five studies reported that nil patients developed some of the outcomes of interest related to this review; 97 studies were included in the meta-analysis. The key characteristics of included studies are outlined in Electronic Supplementary Table S2. The majority of studies had predominant males. The baseline HbA1c ranged between 5.5 and 9.1%, and the mean estimated glomerular filtration rate (eGFR) was between 23.8 and 156.9 mL/min/1.73 m^2^. The mean BMI ranged between 23.1 and 35.1 kg/m^2^, and the duration of diabetes mellitus was between 4 and 24.1 years. The mean (SD) duration of treatment was 0.66 (0.75) years. Although we intended to include any SGLT2i, the eligible studies contained only the following drugs within the class: Empagliflozin, Dapagliflozin, Canagliflozin, Ertugliflozin, Licogliflozin, Luseogliflozin and Sotagliflozin. The most frequently studied SGLT2 inhibitors were empagliflozin (n = 32) and dapagliflozin (n = 31). The predominant clinical indications were T2D (n = 45) and heart failure (n = 29).

### 3.2. Overall Pooled Estimates

**Figure 2:**
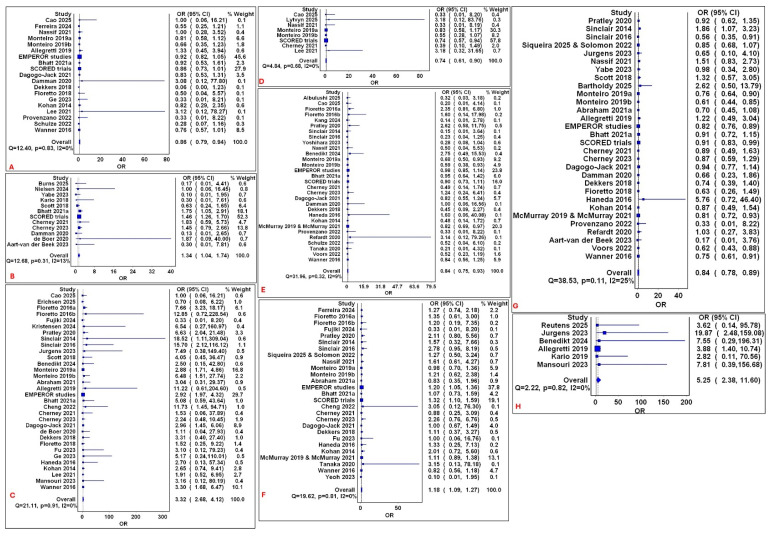
Forest plots of significant overall pairwise pooled estimates for the SGTL2is class. Forest plots of direct comparison pooled estimates of SGLT2i compared to non-SGLT2i for the following outcomes: (**A**): ARF; (**B**): diarrhea; (**C**): genital infections; (**D**): myocardial infarction; (**E**): mortality; (**F**): volume depletion; (**G**): serious adverse events; and (**H**): polyuria. The square box represents the point estimates and the diamond represents the pooled estimate, with horizontal lines representing 95% CI.

Overall, SGLT2 inhibitors demonstrated a favorable benefit–risk profile compared to non-SGLT2i controls, with significant reductions in several major adverse outcomes alongside expected increases in specific class-effect adverse events. In the direct comparison pooled estimates, compared to non-SGLT2i, SGLT2i was associated with a significantly reduced risk of ARF (OR: 0.86; 95% CI: 0.79, 0.94; Figure 2A; 19 studies and 30,825 participants); myocardial infarction (OR: 0.74; 95% CI: 0.61, 0.9; Figure 2D; 8 studies and 13,183 participants); mortality (OR: 0.84; 95% CI: 0.75, 0.93; Figure 2E; 17 studies and 33,733 participants); and SAEs (OR: 0.84; 95% CI: 0.78, 0.89; Figure 2G; 30 studies and 40,991 participants). Conversely, SGLT2i use was associated with an increased risk of diarrhea (OR: 1.34; 95% CI: 1.04, 1.74; Figure 2B; 12 studies and 14,060 participants); genital infections (OR: 3.32; 95% CI: 2.68, 4.12; Figure 2C; 32 studies and 25,971 participants); volume depletion (OR: 1.18; 95% CI: 1.09, 1.27; Figure 2F; 27 studies and 41,528 participants); and polyuria (OR: 5.25; 95% CI: 2.38, 11.6; Figure 2H; 6 studies and 691 participants). Mild heterogeneity was observed, with I^2^ values ranging between 0 and 25%. No significant risks were observed for amputation, constipation, therapy discontinuation, diabetic ketoacidosis, fracture, hepatic dysfunction, hyperkalemia, hypotension, malignancies, stroke, hypoglycemia, and UTI (Electronic Supplementary Figures S1–S12).

### 3.3. Pooled Estimates for Dose Categories

**Figure 3:**
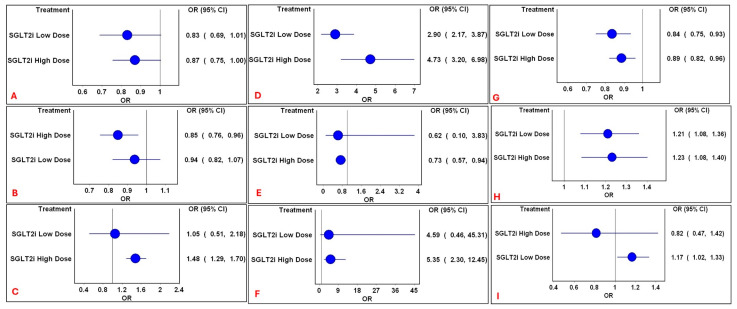
Forest plots of significant mixed treatment comparison pooled estimates between dose categories of SGLT2i. Comparison of significant mixed treatment comparison pooled estimates between dose categories of SGLT2i for the following outcomes: (**A**): ARF; (**B**): mortality; (**C**): diarrhea; (**D**): genital infections; (**E**): myocardial infarction; (**F**): polyuria; (**G**): serious adverse events; (**H**): volume depletion; and (**I**): hypotension. Circles represent the pooled estimates following mixed treatment comparisons with non-SGLT2i. The vertical line represents the line of no difference and horizontal lines represent 95% CI.

The safety profile of SGLT2i was modulated by dose, with higher doses generally amplifying both the beneficial and harmful effects. High doses of SGLT2i were associated with reduced risks of ARF, mortality and myocardial infarction, but increased risks of diarrhea and polyuria (Figure 3). A low dose was associated with an increased risk of hypotension, while both the dose categories were associated with increased risks of genital infections and volume depletion, but a reduced risk of SAEs. Mild heterogeneity was observed (H = 1). No significant differences were observed in the other outcomes (Electronic Supplementary Figures S13–S22).

### 3.4. Pooled Estimates for Age Categories

**Figure 4:**
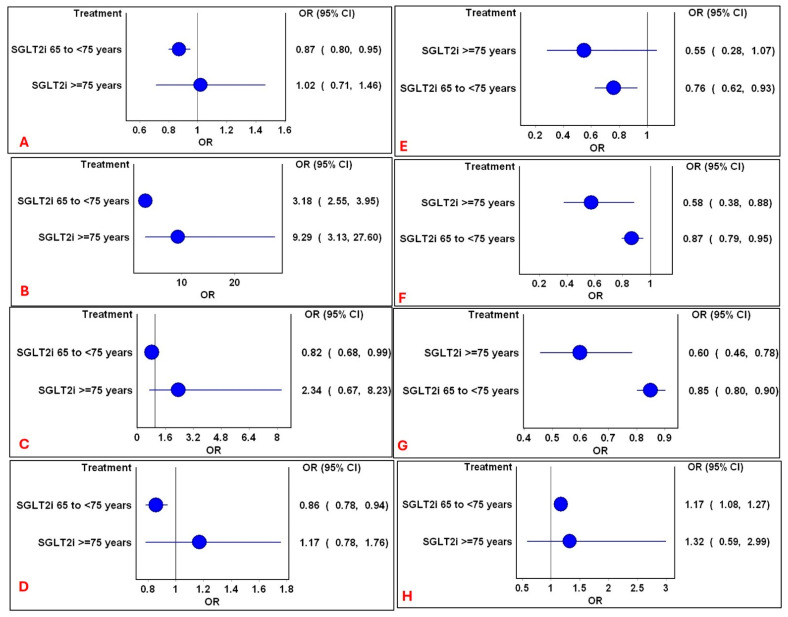
Forest plots of significant mixed treatment comparison pooled estimates between age categories for SGLT2i. Comparison of significant mixed treatment comparison pooled estimates of SGLT2i in various age categories for the following outcomes: (**A**): ARF; (**B**): genital infections; (**C**): hyperkalemia; (**D**): hypoglycemia; (**E**): myocardial infarction; (**F**): mortality; (**G**); serious adverse events; and (**H**): volume depletion. Circles represent the pooled estimates following mixed treatment comparisons with non-SGLT2i. The vertical line represents the line of no difference and horizontal lines represent 95% CI.

Advanced age significantly amplified both the risks and benefits associated with SGLT2i therapy. The mixed treatment comparison pooled estimates for various age categories are outlined in Figure 4. Amongst the age group between 65 and 75 years, there were low risks of ARF, hyperkalemia, hypoglycemia, and myocardial infarction, but an increased risk of volume depletion was observed. An increased risk of genital infection with reduced risks of SAEs and mortality were observed in both age categories. Mild heterogeneity was observed (H = 1). No significant differences were observed in the other outcomes (Electronic Supplementary Figures S23–S27).

### 3.5. Pooled Estimates for Intra-SGLT2i Class of Drugs

**Figure 5:**
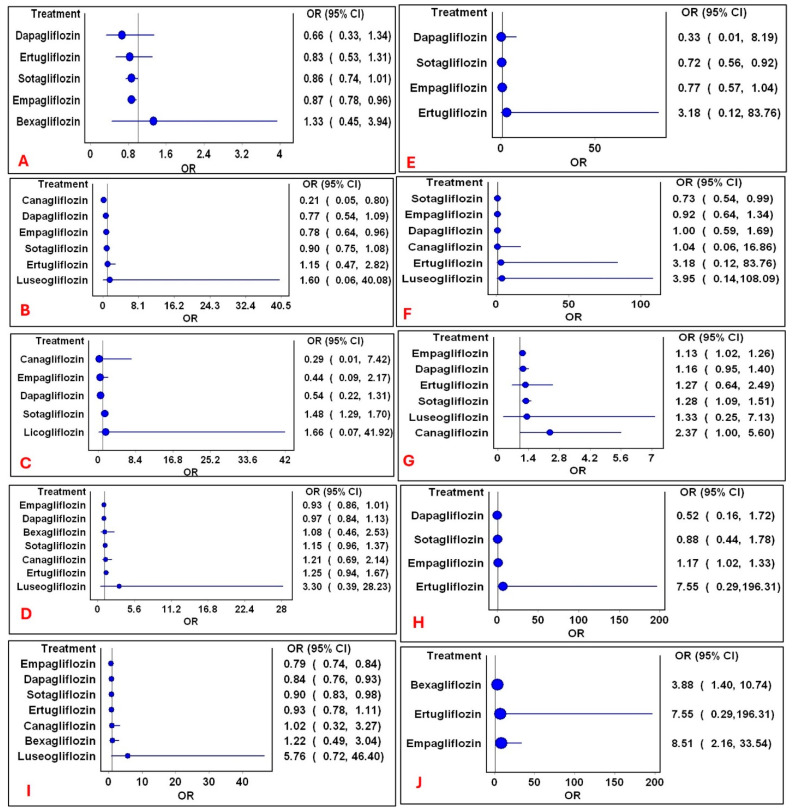
Forest plots of significant mixed treatment comparison pooled estimates within SGLT2i. Comparison of significant mixed treatment comparison pooled estimates of drugs within SGLT2i class for the following outcomes: (**A**): ARF; (**B**): mortality; (**C**): diarrhea; (**D**): discontinuation rate; (**E**): myocardial infarction; (**F**): stroke; (**G**): volume depletion; (**H**): hypotension; (**I**): serious adverse events; and (**J**): polyuria. Circles represent the pooled estimates following mixed treatment comparisons with non-SGLT2i. The vertical line represents the line of no difference and horizontal lines represent 95% CI.

Individual SGLT2i agents exhibited distinct safety profiles, highlighting the clinical relevance of drug selection. The network structure for individual outcomes is depicted in Electronic Supplementary Figure S28. Comparisons of pooled estimates for individual drugs within the SGLT2i class are outlined in Figure 5. Intraclass differences were observed as follows: empagliflozin was observed as having a reduced risk of ARF and mortality, but an increased risk of hypotension and polyuria. Canagliflozin was associated with a reduced risk of mortality. Sotagliflozin was associated with increased risks of diarrhea and volume depletion, but reduced risks of myocardial infarction and stroke. Mild heterogeneity was observed (H = 1). No significant differences were observed in the other outcomes (Electronic Supplementary Figures S29–S36).

### 3.6. Bootstrap and TSA Results

The bootstrap analyses confirmed the statistically significant pooled estimates observed with SGLT2is (Electronic Supplementary Figures S37–S44).

The results of TSA also corroborate the statistically significant pooled estimates observed with SGLT2i compared to non-SGLT2i (Electronic Supplementary Figures S45–S51).

### 3.7. Leave-One-out Sensitivity Analysis, Publication Bias and Risk of Bias Analyses

Leave-one-out sensitivity analysis did not reveal any significant impact on the pooled estimates with the removal of each study in all significant outcomes, except for diarrhea observed with SGLT2i (Electronic Supplementary Tables S4–S11).

Funnel plot and Egger’s regression analyses did not reveal the presence of any publication bias for the significant pooled estimates observed with SGLT2i (Electronic Supplementary Figures S52–S59).

The risk of bias assessment revealed an overall low risk for most of the domains. However, unclear/high risk was observed for allocation concealment and blinding in some studies.

### 3.8. Grading the Strength of Estimates of Key Comparisons

The strengths of evidence for key estimates for SGLT2i were graded as being moderate and were mainly limited by the serious limitations on the potential risk of bias of included studies (Table 2).

### 3.9. Meta-Regression Analyses

The results of meta-regression analyses are summarized in Table 3. Only the treatment duration (≥6 months) was significantly associated with mortality.

## 4. Discussion

### 4.1. Summary of Key Findings

This comprehensive network meta-analysis provides a detailed quantification of the safety profile of SGLT2 inhibitors, specifically in older adults, a population underrepresented in previous pooled analyses. Our results confirm several established class effects, including a significantly increased risk of genital infections and volume depletion, alongside beneficial reductions in the risk of SAEs, ARF, and mortality. Critically, this study goes beyond confirmation to elucidate important modifiers of risk within this vulnerable population. We present three novel findings with direct clinical implications: first, a pronounced dose–response relationship, where higher doses amplified both the benefits (reduced ARF, mortality, MI) and harms (genital infections, diarrhea, polyuria); second, a marked age-dependent effect, with the risk of genital infections escalating sharply (nine-fold) and the mortality benefit becoming most pronounced in adults ≥75 years; and third, significant intra-class differences in safety profiles among individual SGLT2i agents. These findings underscore that the safety profile of SGLT2i in older adults is not uniform, but is critically influenced by the specific drug choice, dosage, and patient age, offering nuanced evidence to guide clinical decision-making.

### 4.2. Comparison with Existing Literature

Our analysis robustly confirms the well-documented renoprotective and cardioprotective benefits of SGLT2i in the older adult population. This aligns with known mechanisms, including natriuresis, enhanced renal blood flow via vascular endothelial growth factors, and attenuation of inflammatory pathways and the renin–angiotensin system [21,22]. Importantly, SGLT2i may directly counter the key processes of kidney aging, such as oxidative stress and cellular senescence [23]. Our results corroborate real-world evidence showing reduced eGFR decline (OR = 0.6, 95% CI:0.38–0.96) in older adults with T2D and hypertension [24]. This nephroprotection appears to be unique to the SGLT2i class and extends to non-diabetic conditions like CKD [25,26], underscoring a fundamental tissue-protective property. The consistent renal benefit supports the use of SGLT2i as a first-line therapy for kidney protection in older adults, even in the presence of mild-to-moderate age-related eGFR decline.

We also confirm the predictable class-effect increases in genital infections and volume depletion. The increased risk of genital infections, driven by pharmacologically induced glycosuria [27], is a consistent finding. Our results extend this by quantifying the substantial clinical impact, noting its association with therapy discontinuation [28] and its occurrence, even in males [28], albeit with a reduced risk in circumcised men [29]. Similarly, the increased risk of volume depletion is consistent with the osmotic diuresis induced by SGLT2i, which can be compounded by age-related physiological changes such as a blunted thirst response and autonomic dysfunction [30,31].

Several novel findings deserve a mention in this study. First, we identified a clear dose–response relationship. Higher doses were associated with greater reductions in ARF, mortality, and MI, but also with a disproportionately higher risk of genital infections (OR 4.73 vs. 2.90 with low dose) and other adverse effects like diarrhea and polyuria. This trade-off suggests that initiating therapy at a lower dose in high-risk older adults may be a prudent strategy to balance efficacy and tolerability. Second, advanced age (≥75 years) emerged as a critical effect modifier. The nine-fold increased risk of genital infections in this subgroup is a striking finding, likely reflecting the confluence of glycosuria with age-related immunological senescence and urogenital mucosal changes [32]. Paradoxically, this oldest subgroup also derived the most substantial mortality benefit (OR 0.58). This underscores a vital clinical distinction: while the risk of manageable side effects like genital infections escalates with age, so does the potential for preventing catastrophic outcomes. Therefore, concern over these risks should not lead to therapeutic inertia in the very elderly. Third, we identified significant intra-class differences that were previously unquantified in older adults. Empagliflozin was associated with reduced ARF and mortality but increased hypotension and polyuria. Canagliflozin showed a mortality benefit. Sotagliflozin, a dual SGLT1/2 inhibitor, displayed a unique profile with increased diarrhea and volume depletion, but reduced MI and stroke. These distinctions are clinically relevant for personalized prescribing; for instance, sotagliflozin might be considered in patients with high cardiovascular risk but require careful monitoring for volume status, especially in those prone to dehydration.

In summary, our analysis affirms that SGLT2 inhibitors retain their foundational cardiorenal and mortality benefits in older adults while carrying a predictable and manageable safety profile. However, the risks are not static; they are meaningfully influenced by drug choice, dose, and patient age. Clinical practice must evolve from a blanket class-level assessment to a nuanced, patient-centric approach. For the very elderly (≥75 years), the substantial mortality benefit must be weighed against a markedly higher risk of genital infections, necessitating proactive patient education and hygiene measures. Initiating therapy at a lower dose may be a reasonable strategy to mitigate adverse events associated with increasing dose ranges. The distinct safety profiles of individual agents offer opportunities for tailored therapy based on a patient’s specific risk profile. By proactively managing modifiable risks, clinicians can confidently harness the substantial survival advantage these agents offer, ensuring that older adults are not deprived of this transformative therapy due to disproportionate fear of its side effects.

Based on our findings, we propose the following practical recommendations for prescribing SGLT2i in older adults. For all older adults: Initiate therapy with a clear discussion of the common, manageable side effects, particularly genital infections and volume depletion. Counsel patients on preventive hygiene measures and the importance of maintaining adequate hydration, especially during intercurrent illness or hot weather. For adults aged ≥75 years: Recognize that this subgroup derives the greatest mortality benefit but also faces the highest risk of genital infections (nine-fold). Proactive monitoring and education are paramount. Do not withhold therapy due to fear of this manageable side effect. Regarding dose selection: Consider initiating treatment with a lower dose in frail individuals or those at high risk for volume depletion or genital infections to improve tolerability. The dose can be up-titrated later, based on clinical response and tolerability, balancing the enhanced efficacy of higher doses against their increased risk of adverse effects. Regarding drug selection: Leverage the distinct safety profiles of individual agents. For example, sotagliflozin may be considered in patients with high cardiovascular risk but requires careful monitoring for volume depletion and diarrhea. Empagliflozin’s favorable renal profile may be preferred in patients with chronic kidney disease.

### 4.3. Strengths, Limitations and Way Forward

This study has several strengths. First, it is the first comprehensive network meta-analysis to specifically quantify the comparative safety profile of SGLT2i in the older adult population, a group with distinct physiological and clinical vulnerabilities. The use of NMA allowed for the indirect comparison of individual drugs, dose categories, and age subgroups in the absence of direct head-to-head trials, generating novel, clinically relevant insights. Second, the analysis adhered to rigorous methodological standards, including a pre-registered protocol, a broad literature search without language restrictions, dual independent data extraction, and comprehensive risk-of-bias and publication bias assessments. Third, the robustness of the findings was confirmed through multiple sensitivity analyses, including bootstrapping and TSA, which support the reliability and stability of the pooled estimates. Finally, the application of the GRADE framework and meta-regression provides a transparent assessment of the evidence certainty and explores potential sources of heterogeneity, enhancing the interpretability and clinical utility of our results.

However, the findings of this review should be interpreted by considering several limitations. First, while the NMA enabled indirect comparisons, the results for specific drug-to-drug or subgroup comparisons are inherently less robust than those from dedicated, large-scale randomized trials. Second, despite the large overall sample, the number of events for certain safety outcomes (such as amputations and ketoacidosis) and within specific subgroups (particularly adults ≥ 75 years) remained limited, leading to wide confidence intervals and precluding definitive conclusions for these rarer events. Third, our analyses were conducted at the study level; patient-level data were not available, which restricted our ability to adjust for important confounding variables (such as frailty status, comorbidities, concomitant medications) or to perform more granular subgroup analyses. The absence of frailty data is a particularly important limitation, as frailty is a major determinant of vulnerability to adverse drug events in geriatric populations and may significantly modify the risk–benefit calculus observed in our study. Fourth, the included trials were primarily designed to assess efficacy, and safety reporting may have been inconsistent or incomplete. It is important to note that safety outcomes were often secondary endpoints, which may have led to less rigorous ascertainment and under-reporting of certain adverse events compared to trials that were designed with safety as a primary outcome. Fifth, while discrepancies in study selection and data extraction were resolved through discussion and consensus between the two reviewers, a standard practice in systematic reviews, we acknowledge the theoretical limitation that in the absence of a predefined arbitration process, unresolved disagreements could potentially introduce bias, although this did not occur in the present review. Furthermore, most included trials had follow-up durations of only a few months, leaving the long-term safety profile of SGLT2i in older adults uncertain.

Future research should prioritize the conduct of large-scale, pragmatic trials or well-designed observational studies that are prospectively powered to evaluate safety endpoints in older adults, with deliberate inclusion of the very old and frail. Patient-level meta-analyses would be invaluable for exploring effect modifiers and identifying specific risk factors for adverse events within this heterogeneous population. Further investigation into the mechanistic basis for the observed intra-class differences and the marked age-related increase in genital infection risk is warranted to inform drug selection and preventive strategies. In the interim, clinicians should incorporate our findings, particularly the strong mortality benefit, age-dependent risks, and the distinct drug profiles, into shared decision-making, balancing class-wide benefits with individualized risk assessment, based on patient age, functional status, and the specific SGLT2i drug.

## 5. Conclusions

In conclusion, this systematic review and NMA provides robust, quantitative evidence that SGLT2i offer a favorable benefit–risk profile in older adults, conferring significant reductions in mortality, SAE, and ARF compared to non-SGLT2i therapies. The class is associated with increased risks of genital infections and volume depletion, risks that are demonstrably modulated by drug choice, dosage, and patient age. Specifically, the risk of genital infections exhibits a strong dose–response relationship and increases markedly in the oldest adults, while the mortality benefit appears to be most pronounced in those aged 75 years and older. These findings affirm the overall safety of SGLT2i in geriatric care while highlighting the necessity for a personalized, vigilant approach to prescribing. Clinicians should integrate these nuanced safety signals, balancing the compelling class-wide benefits against agent-specific and patient-specific risk factors, to optimize therapeutic outcomes for a growing and vulnerable older adult population.

## Figures and Tables

**Table 1:** Summary of comparisons and covariates included in the network meta-analysis.

Analysis Category	Specific Comparisons/Strata	Covariates Assessed in Meta-Regression
Age Groups	65 to <75 years ≥75 years	
Dose Categories	Low dose High dose	
Intra-Class Comparisons	Individual SGLT2i drugs (such as Empagliflozin, Dapagliflozin, Canagliflozin, and Sotagliflozin, along with several others)	
Meta-Regression		Body Mass Index (BMI): <30 vs. ≥30 kg/m^2^ eGFR: <90 vs. ≥90 mL/min/1.73 m^2^ Primary Indication: Diabetes vs. Non-diabetes Treatment Duration: <6 months vs. ≥6 months Trial Design: Blinded vs. Open label

**Table 2:** Grading the strength of key comparison estimates.

Key Outcomes with SGLT2i Compared to Non-SGLT2i in Older Adults	Comparative Risks per 1000 Patients (95% Confidence Intervals)		Effect Estimates and the Quality of Evidence for the Pooled Estimates
	Assumed Risk ^1^	Corresponding Risk	
ARF	44	38 (35 to 41)	0.86 [0.79, 0.94]; Moderate ^2^
Diarrhea	50	66 (52 to 84)	1.34 [1.04, 1.74]; Moderate ^2^
Genital infections	7	23 (19 to 28)	3.32 [2.68, 4.12]; Moderate ^2^
Myocardial infarction	23	17 (14 to 21)	0.74 [0.61, 0.9]; Moderate ^2^
Mortality	45	38 (34 to 42)	0.84 [0.75, 0.93]; Moderate ^2^
Volume depletion	40	47 (44 to 50)	1.18 [1.09, 1.27]; Moderate ^2^
Serious adverse event	205	178 (167 to 187)	0.84 [0.78, 0.89]; Moderate ^2^

^1^—Median risk in the non-SGLT2i arm; ^2^—downgraded one level for serious limitations in the potential risk of bias of included studies. Moderate: The estimated effect is close to the true effect, but there remains a reasonable possibility that future well-conducted studies could change the magnitude of the estimate and, in some cases, the direction of the effect.

**Table 3:** Summary of multivariable meta-regression analyses.

Covariates	OR with 95% CI			
	Genital Infection	Mortality	Volume Depletion	Serious Adverse Events
Indications (compared to non-diabetes)	0.68 [0.22, 2.11]	1.55 [0.98, 2.46]	0.97 [0.57, 1.64]	1.08 [0.81, 1.45]
eGFR (compared to ≥90 mL/min/1.73m^2^)	1.80 [0.01, 286.68]	3.34 [0.38, 29.61]	Not estimable	
BMI (compared to <30 kg/m^2^)	0.85 [0.19, 3.83]	1.46 [0.92, 2.31]	0.96 [0.57, 1.62]	1.13 [0.87, 1.48]
Treatment duration (compared to <6 months)	0.63 [0.14, 2.90]	2.56 [1.05, 6.27] *	0.87 [0.48, 1.59]	1.06 [0.81, 1.38]
Blinding (compared to blinding)	0.42 [0.02, 10.59]	0.56 [0.22, 1.41]	0.54 [0.17, 1.73]	0.98 [0.53, 1.81]

* Statistically significant.

## Data Availability

The data presented in this study are available on request from the corresponding author. (Some of the data are not publicly available due to privacy).

## Supplementary Materials

The following supporting information can be downloaded at: https://www.mdpi.com/article/10.3390/medsci14010153/s1, Electronic Supplementary Table S1. Search strategy; Electronic Supplementary Table S2. Classification of doses of SGLT2i; Electronic Supplementary Table S3. Key characteristics of the included studies; Electronic Supplementary Table S4. Leave-one-out sensitivity analysis for SGLT2is compared to non-SGLT2i group for mortality; Electronic Supplementary Table S5. Leave-one-out sensitivity analysis for SGLT2is compared to non-SGLT2i group for ARF; Electronic Supplementary Table S6. Leave-one-out sensitivity analysis for SGLT2is compared to non-SGLT2i group for diarrhea; Electronic Supplementary Table S7. Leave-one-out sensitivity analysis for SGLT2is compared to non-SGLT2i group for genital infections; Electronic Supplementary Table S8. Leave-one-out sensitivity analysis for SGLT2is compared to non-SGLT2i group for volume depletion; Electronic Supplementary Table S9. Leave-one-out sensitivity analysis for SGLT2is compared to non-SGLT2i group for polyuria; Electronic Supplementary Table S10. Leave-one-out sensitivity analysis for SGLT2is compared to non-SGLT2i group for serious adverse events; Electronic Supplementary Table S11. Leave-one-out sensitivity analysis for SGLT2is compared to non-SGLT2i group for myocardial infarction; Electronic Supplementary Figure S1. Forest plot for the risk of amputation with SGLT2i compared to non-SGLT2i; Electronic Supplementary Figure S2. Forest plot for the risk of constipation with SGLT2i compared to non-SGLT2i; Electronic Supplementary Figure S3. Forest plot for the risk of therapy discontinuation with SGLT2i compared to non-SGLT2i; Electronic Supplementary Figure S4. Forest plot for the risk of diabetic ketoacidosis with SGLT2i compared to non-SGLT2i; Electronic Supplementary Figure S5. Forest plot for the risk of fracture with SGLT2i compared to non-SGLT2i; Electronic Supplementary Figure S6. Forest plot for the risk of hepatic dysfunction with SGLT2i compared to non-SGLT2i; Electronic Supplementary Figure S7. Forest plot for the risk of hyperkalemia with SGLT2i compared to non-SGLT2i; Electronic Supplementary Figure S8. Forest plot for the risk of hypotension with SGLT2i compared to non-SGLT2i; Electronic Supplementary Figure S9. Forest plot for the risk of malignancies with SGLT2i compared to non-SGLT2i; Electronic Supplementary Figure S10. Forest plot for the risk of stroke with SGLT2i compared to non-SGLT2i; Electronic Supplementary Figure S11. Forest plot for the risk of hypoglycemia with SGLT2i compared to non-SGLT2i; Electronic Supplementary Figure S12. Forest plot for the risk of UTI with SGLT2i compared to non-SGLT2i; Electronic Supplementary Figure S13. Forest plot for mixed treatment comparison pooled estimates for the risk of constipation with various doses of SGLT2is compared to non-SGLT2i group; Electronic Supplementary Figure S14. Forest plot for mixed treatment comparison pooled estimates for the risk of therapy discontinuation with various doses of SGLT2is compared to non-SGLT2i group; Electronic Supplementary Figure S15. Forest plot for mixed treatment comparison pooled estimates for the risk of diabetic ketoacidosis with various doses of SGLT2is compared to non-SGLT2i group; Electronic Supplementary Figure S16. Forest plot for mixed treatment comparison pooled estimates for the risk of fracture with various doses of SGLT2is compared to non-SGLT2i group; Electronic Supplementary Figure S17. Forest plot for mixed treatment comparison pooled estimates for the risk of hepatic dysfunction with various doses of SGLT2is compared to non-SGLT2i group; Electronic Supplementary Figure S18. Forest plot for mixed treatment comparison pooled estimates for the risk of hyperkalemia with various doses of SGLT2is compared to non-SGLT2i group; Electronic Supplementary Figure S19. Forest plot for mixed treatment comparison pooled estimates for the risk of hypoglycemia with various doses of SGLT2is compared to non-SGLT2i group; Electronic Supplementary Figure S20. Forest plot for mixed treatment comparison pooled estimates for the risk of malignancies with various doses of SGLT2is compared to non-SGLT2i group; Electronic Supplementary Figure S21. Forest plot for mixed treatment comparison pooled estimates for the risk of stroke with various doses of SGLT2is compared to non-SGLT2i group; Electronic Supplementary Figure S22. Forest plot for mixed treatment comparison pooled estimates for the risk of urinary tract infections with various doses of SGLT2is compared to non-SGLT2i group; Electronic Supplementary Figure S23. Forest plot for mixed treatment comparison pooled estimates for the risk of therapy discontinuation of SGLT2is compared to non-SGLT2i group amongst various age groups; Electronic Supplementary Figure S24. Forest plot for mixed treatment comparison pooled estimates for the risk of fracture with SGLT2is compared to non-SGLT2i group amongst various age groups; Electronic Supplementary Figure S25. Forest plot for mixed treatment comparison pooled estimates for the risk of malignancies with SGLT2is compared to non-SGLT2i group amongst various age groups; Electronic Supplementary Figure S26. Forest plot for mixed treatment comparison pooled estimates for the risk of stroke with SGLT2is compared to non-SGLT2i group amongst various age groups; Electronic Supplementary Figure S27. Forest plot for mixed treatment comparison pooled estimates for the risk of urinary tract infection with SGLT2is compared to non-SGLT2i group amongst various age groups; Electronic Supplementary Figure S28. Network plot for mixed treatment comparison pooled estimates for the individual SGLT2is compared to non-SGLT2i group across outcomes; Electronic Supplementary Figure S29. Forest plot for mixed treatment comparison pooled estimates for the risk of constipation with individual SGLT2is compared to non-SGLT2i group; Electronic Supplementary Figure S30. Forest plot for mixed treatment comparison pooled estimates for the risk of hypoglycemia with individual SGLT2is compared to non-SGLT2i group; Electronic Supplementary Figure S31. Forest plot for mixed treatment comparison pooled estimates for the risk of hyperkalemia with individual SGLT2is compared to non-SGLT2i group; Electronic Supplementary Figure S32. Forest plot for mixed treatment comparison pooled estimates for the risk of fracture with individual SGLT2is compared to non-SGLT2i group; Electronic Supplementary Figure S33. Forest plot for mixed treatment comparison pooled estimates for the risk of hepatic dysfunction with individual SGLT2is compared to non-SGLT2i group; Electronic Supplementary Figure S34. Forest plot for mixed treatment comparison pooled estimates for the risk of amputation with individual SGLT2is compared to non-SGLT2i group; Electronic Supplementary Figure S35. Forest plot for mixed treatment comparison pooled estimates for the risk of DKA with individual SGLT2is compared to non-SGLT2i group; Electronic Supplementary Figure S36. Forest plot for mixed treatment comparison pooled estimates for the risk of malignancies with individual SGLT2is compared to non-SGLT2i group; Electronic Supplementary Figure S37. Bootstrap histogram for the pooled estimates for the risk of volume depletion with SGLT2is compared to non-SGLT2i group; Electronic Supplementary Figure S38. Bootstrap histogram for the pooled estimates for the risk of mortality with SGLT2is compared to non-SGLT2i group; Electronic Supplementary Figure S39. Bootstrap histogram for the pooled estimates for the risk of polyuria with SGLT2is compared to non-SGLT2i group; Electronic Supplementary Figure S40. Bootstrap histogram for the pooled estimates for the risk of serious adverse events with SGLT2is compared to non-SGLT2i group; Electronic Supplementary Figure S41. Bootstrap histogram for the pooled estimates for the risk of diarrhea with SGLT2is compared to non-SGLT2i group; Electronic Supplementary Figure S42. Bootstrap histogram for the pooled estimates for the risk of myocardial infarction with SGLT2is compared to non-SGLT2i group; Electronic Supplementary Figure S43. Bootstrap histogram for the pooled estimates for the risk of genital infections with SGLT2is compared to non-SGLT2i group; Electronic Supplementary Figure S44. Bootstrap histogram for the pooled estimates for the risk of ARF with SGLT2is compared to non-SGLT2i group; Electronic Supplementary Figure S45. TSA plot for the risk of ARF with SGLT2is compared to non-SGLT2i group; Electronic Supplementary Figure S46. TSA plot for the risk of mortality with SGLT2is compared to non-SGLT2i group; Electronic Supplementary Figure S47. TSA plot for the risk of SAE with SGLT2is compared to non-SGLT2i group; Electronic Supplementary Figure S48. TSA plot for the risk of genital infections with SGLT2is compared to non-SGLT2i group; Electronic Supplementary Figure S49. TSA plot for the risk of polyuria with SGLT2is compared to non-SGLT2i group; Electronic Supplementary Figure S50. TSA plot for the risk of myocardial infarction with SGLT2is compared to non-SGLT2i group; Electronic Supplementary Figure S51. TSA plot for the risk of diarrhea with SGLT2is compared to non-SGLT2i group; Electronic Supplementary Figure S52. Funnel plot for the risk of diarrhea with SGLT2is compared to non-SGLT2i group; Electronic Supplementary Figure S53. Funnel plot for the risk of ARF with SGLT2is compared to non-SGLT2i group; Electronic Supplementary Figure S54. Funnel plot for the risk of mortality with SGLT2is compared to non-SGLT2i group; Electronic Supplementary Figure S55. Funnel plot for the risk of SAE with SGLT2is compared to non-SGLT2i group; Electronic Supplementary Figure S56. Funnel plot for the risk of polyuria with SGLT2is compared to non-SGLT2i group; Electronic Supplementary Figure S57. Funnel plot for the risk of volume depletion with SGLT2is compared to non-SGLT2i group; Electronic Supplementary Figure S58. Funnel plot for the risk of myocardial infarction with SGLT2is compared to non-SGLT2i group; Electronic Supplementary Figure S59. Funnel plot for the risk of genital infections with SGLT2is compared to non-SGLT2i group. References [33,34,35,36,37,38,39,40,41,42,43,44,45,46,47,48,49,50,51,52,53,54,55,56,57,58,59,60,61,62,63,64,65,66,67,68,69,70,71,72,73,74,75,76,77,78,79,80,81,82,83,84,85,86,87,88,89,90,91,92,93,94,95,96,97,98,99,100,101,102,103,104,105,106,107,108,109,110,111,112,113,114,115,116,117,118,119,120,121,122,123,124,125,126,127,128,129,130,131,132,133,134] are cited in the supplementary materials.
