# Supplementary material for: Safety Profile of SGLT-2 Inhibitors in Older Adults: A Systematic Review and Network Meta-Analysis
Source: Med Sci (Basel). 2026 Mar 20;14(1):153. doi: 10.3390/medsci14010153 (PMC13028317; doi:10.3390/medsci14010153)

Electronic Supplementary Table S1. Search strategy.

**PubMed**
((SGLT-2 inhibitor[Title/Abstract] OR sodium glucose transporter[Title/Abstract] OR canagliflozin[Title/Abstract] OR dapagliflozin[Title/Abstract] OR empagliflozin[Title/Abstract] OR ertugliflozin[Title/Abstract] OR sotagliflozin[Title/Abstract] OR bexagliflozin[Title/Abstract]) AND (random[Title/Abstract] OR randomized[Title/Abstract] OR randomised[Title/Abstract])) NOT (Review[Publication Type] OR Meta-analysis[Publication Type])
**Cochrane CENTRAL**
#1 ("SGLT-2"):ti,ab,kw (Word variations have been searched)<br>#2 ("sodium glucose co-transporter 2"):ti,ab,kw (Word variations have been searched)<br>#3 (canagliflozin):ti,ab,kw (Word variations have been searched)<br>#4 (dapagliflozin):ti,ab,kw (Word variations have been searched)<br>#5 (empagliflozin):ti,ab,kw (Word variations have been searched)<br>#6 (ertugliflozin):ti,ab,kw (Word variations have been searched)<br>#7 (sotagliflozin):ti,ab,kw (Word variations have been searched)<br>#8 (bexagliflozin):ti,ab,kw (Word variations have been searched)<br>#9 #1 OR #2 OR #3 OR #4 OR #5 OR #6 OR #7 OR #8<br>#10 ("randomized clinical trial"):ti,ab,kw (Word variations have been searched)<br>#11 #9 AND #10

Electronic Supplementary Table S2. Classification of doses of SGLT2i.

SGLT2i	Low doses (mg/day)	High doses (mg/day)
Bexagliflozin	≤5	≥20
Canagliflozin	≤100	≥300
Dapagliflozin	≤5	≥10
Empagliflozin	≤10	≥25
Ertugliflozin	≤5	≥15
Licogliflozin	≤5	≥10
Luseogliflozin	≤2.5	≥5
Sotagliflozin	≤200	≥400

Electronic Supplementary Table S3. Key characteristics of the included studies.

Study id	Indications	Interventions	Mean/Median age (years)	Male: Female (%)	HbA1c (%)	eGFR (ml/min/1.73m^2)	BMI (kg/m^2)	Duration of diabetes mellitus (years)	Dosing regimen	Duration of treatment (years)
Abraham 2021a (EMPERIAL-Reduced) [33]	HF	E	69	77.6: 22.4	No details	56.8	No details		10 mg/day	0.23
		Placebo	70	71.2: 28.8		53			Not applicable	
Abraham 2021b (EMPERIAL-Preserved) [33]		E	74	55.4: 44.6		54.5			10 mg/day	
		Placebo	75	58.2: 41.8		58.5			Not applicable	
Allegretti 2019 [34]	T2D/CKD	B	69.3	58.6: 41.4	8.01	45.4	30.29	15.54	20 mg/day	3
		Placebo	69.9	67.1: 32.9	7.95	44.78	30.1	16.28	Not applicable	
Anker 2021a [35]	HF without T2D	E	67.6	75.7: 24.3	5.8	62.7	27.2	No details	10 mg/day	1
		Placebo	66.3	74.6: 25.4	5.7	63	27		Not applicable	
	HF/T2D	E	66.8	77.3: 22.7	7.4	61	28.8		10 mg/day	
		Placebo	66.6	76.5: 23.5	7.4	61.4	28.6		Not applicable	
Anker 2021b [36]	HF	E	71.8	55.4: 44.6	No details	60.6	29.77		10 mg/day	Median (2.2)
		Placebo	71.9	55.3: 44.7		60.6	29.9		Not applicable	
Bhatt 2021a [37]	T2D/HF	S	69	67.4: 32.6	7.1	49.2	30.4		200 mg/day titrated to 400 mg/day	Median (0.75)

		Placebo	70	65.1: 34.9	7.2	50.5	31.1		Not applicable	
Bhatt 2021b [38], Sridhar 2024 [39] & Aggarwal 2025 [40]	T2D/CKD	S	69	55.7: 44.3	8.3	44.4	31.9		200 mg/day titrated to 400 mg/day	Median (1.33)
		Placebo	69	54.5: 45.5	8.3	44.7	31.7		Not applicable	
Charaya 2022 [41]	HF	D	72.6	58: 42	No details	55.65	No details		10 mg/day	0.1
		Standard of care	74.2	52: 48		52.7			Not applicable	
Cheng 2022 [42]	T2D/HT	E	71.2	64.5: 35.5	7.7	83	25.5	No details	25 mg/day	0.25
		Placebo	71.7	59.7: 40.3	7.7	81.5	25.8		Not applicable	
Cherney 2021 [43]	T2D/ CKD stage IV	S	66.8	47.8: 52.2	8.3	23.8	30.9	19.6	200 mg/day	
		S	67.3	53.3: 46.7	8.3	23.9	32.1	18.5	400 mg/day	
		Placebo	68	45.2: 54.8	8.4	24.1	31.7	20.7	Not applicable	
Cherney 2023 [44]	T2D/ CKD stage III	S	69.6	54.4: 45.6	8.3	45.2	32.3	17.2	200 mg/day	0.5
		S	69.5	57.6: 42.4	8.3	45.1	32.4	16.3	400 mg/day	
		Placebo	69.3	57.3: 42.7	8.3	44.8	32.5	17.7	Not applicable	
Dagogo-Jack 2021 [45]	T2D/CKD	Er	68.3	64.1: 35.9	8.2	49.6	32.3	15.3	5 mg/day	0.35
		Er	68.2	62.7: 37.3	8.2	49.4	32.4	15.2	15 mg/day	
		Placebo	68	66.1: 33.9	8.2	49	32.6	16.4	Not applicable	
Damman 2020 [46]	HF	E	79	60: 40	No details	55	No details		10 mg/day	0.16
		Placebo	73	74: 26		55			Not applicable	
de Boer 2020 [47]	T2D/HF	L	70	93.3: 6.7		76.3	33.3		2.5 mg/day	0.25
		L	72.5	75: 25		61.3	31.9		10 mg/day	
		L	66	80: 20		69.8	32		50 mg/day	

		E	68.5	67.7: 33.3		63.5	31.2		25 mg/day	
		Placebo	71	57.6: 42.4		66.5	31.3		Not applicable	
Dekkers 2018 [48]	T2D/ CKD stage III/IV	D	66	55.2: 44.8	8.4	37.6	34.7	17.2	5 mg/day	2
		D	66.3	52.7: 47.3	8.2	38	34.8	16.7	10 mg/day	
		Placebo	66.5	58: 42	8.1	38.4	34.6	13.5	Not applicable	
Fioretto 2018 [49]	T2D/ CKD stage III	D	65.3	56.9: 43.1	8.33	53.3	32.6	14.3	10 mg/day	0.5
		Placebo	66.2	56.5: 43.5	8.03	53.6	31.6	14.5	Not applicable	
Fu 2023 [50]	T2D/HF	D	70.7	70: 30	7.9	67.5	24.7	No details	10 mg/day	1
		Placebo	70.4	73.3: 26.7	8	67.5	24.7		Not applicable	
Ge 2023 [51]	PAH	D/Sacubitril/Valsartain	68	60: 40	No details		23.4	No details	D-5 to 10 mg/day, Sacubitril/Valsartain-100 mg/day	0.5
		Sacubitril/Valsartain	69	48.33: 51.67			23.5		100 mg/day	
		Placebo	67.5	46.8: 53.2	8.1	156.9	33.2	13.1	Not applicable	
Haneda 2016 [52]	T2D/CKD	Lu	68.4	75.8: 24.2	7.72	52	25.45	10.4	2.5-5 mg/day	1
		Placebo	67.9	78: 22	7.69	52.4	25.81	12.6	Not applicable	
Kario 2019 [53]	T2D	E	70.9	52.9: 47.1	6.6	68.4	26.1	10.6	10 mg/day	0.25
		Placebo	69.3	52.4: 47.6	6.6	70.1	26	9.6	Not applicable	
Katsiadas 2022 [54]	T2D	D	68.11	83.9: 16.1	7.45	72	27.6	No details	10 mg/day	1
		Control	71.87	71.87: 28.13	7.2	68.8	27.9		Not specified	

Kohan 2014 [55]	T2D/CKD	D	66	66.3: 33.7	8.3	No details		16.9	5 mg/day	2
		D	68	65.9: 34.1	8.22			18.2	10 mg/day	
		Placebo	67	63.1: 36.9	8.53			15.7	Not applicable	
Lee 2021 [56]	T2D/ Prediabetes with HF	E	68.2	65.4: 34.6		69.5	30.9	8.2	10 mg/day	0.7
		Placebo	69.2	81.1: 18.9		65.1	30.4	10	Not applicable	
Lundin 2022 [57]	ACS	E	67	80: 20	6	68	27	No details	25 mg/day	0.83
		Placebo	68	81.8: 18.2	6.1	73	27		Not applicable	
Mansouri 2023 [58]	T2D/RA	E	67.46	73: 27	7.43	No details	27.03	No details	25 mg/day	0.16
		Placebo	65.47	60.5: 39.5	7.42		28.29		Not applicable	
McMurray 2019 [59]	HF	D	66.2	76.2: 23.8	No details	66	28.2	No details	10 mg/day	Median (1.5)
		Placebo	66.5	77: 23		65.5	28.1		Not applicable	
Packer 2020 [60]	HF	E	67.2	76.5: 23.5	No details	61.8	28		10 mg/day	Median (1.33)
		Placebo	66.5	75.6: 24.4		62.2	27.8		Not applicable	
Palau 2022 [61]	HF	D	69.8	77.8: 22.2		64.1	27.3		10 mg/day	0.25
		Placebo	67.3	75.6: 24.4		69.4	28.1		Not applicable	
Provenzano 2022 [62]	CKD	D	69.5	76.1: 23.9	No details	58.1	No details		10 mg/day	0.08
		Eplerenone							50 mg/day	
		D/Eplerenone							D-10 mg/day & eplerenone-50 mg/day	
Qiu 2014 [63]	T2D	C	58.6	43: 57	7.6	86.9	33	6.7	100 mg/day	0.35
		C	56.7	47.3: 52.7	7.6	85.9	32.3	7.3	300 mg/day	
		Placebo	57	49.5: 50.5	7.7	84.8	32.3	7	Not	

									applicable	
Ramirez-Rodriguez 2018 [64]	Prediabetes	D	51.5	80: 20	5.8	No details	30.3	No details	10 mg/day	0.25
		Placebo	46.7	75: 25	5.8		33		Not applicable	
Rau 2021 [65]	T2D	E	62.8	80: 20	No details	77	31.4		10 mg/day	0.25
		Placebo	61.2	81.8: 18.2		88	31.2		Not applicable	
RECOVERY 2023 [66]	COVID-19	E	61.5	63: 37	No details				10 mg/day	0.08
		Standard of care		62: 38					Not applicable	
Refardt 2020 [67]	SIADH	E	74	37: 63	No details		24	No details	25 mg/day	0.01
		Placebo	76	36: 64			23.1		Not applicable	
Schulze 2022 [68]	HF	E	72.9	63.3: 36.7	6.6	58.2	31.1	No details	25 mg/day	0.01
		Placebo	76.5	58.6: 41.4	6.4	62.2	29.9		Not applicable	
Scott 2018 [69]	T2D	D	66.6	60.8: 39.2	7.8	76.9	31.5	10.7	5-10 mg/day	0.5
		Sitagliptin	65.7	55: 45	7.7	79.4	31.8	10.5	100 mg/day	
Tanaka 2020 [70]	T2D/HF	C	68.3	77.9: 22.1	No details		24.1	No details	100 mg/day	0.5
		Glimepiride	68.9	71.7: 28.3			25.4		0.5-6 mg/day	
van Aart-van der Beek 2023 [71]	T2D	D, Exenatide and D/Exenatide to each group in cross-over trial	70.5	80: 20		60.4	33.2	No details	D-10 mg/d Exenatide – 2 mg/week	0.12
van Bommel 2020 [72]	T2D	D	63	79: 21		85	No details	9.8	10 mg/day	0.25
		Gliclazide	63	75: 25		89		10.7	30 mg/day	
Van Meijel 2020 [73]	T1D	D & Placebo (crossover trial)	49.7	40: 60	7.5	No details	25.1	24.1	10 mg/day	0.17

Vianna 2020 [74]	T2D	D	57	46.7: 53.3	No details	89	31.2	4	10 mg/day	0.25
		Gliclazide	58.6	53.8: 46.2		87	30.6	4	120 mg/day	
Vilsboll 2019 [75] & Vilsboll 2020 [76]	T2D	D/Saxagliptin/ Metformin	55.7	54.3: 45.7	9	94.6	32.5	9.6	D-10 mg/day, saxagliptin-5 mg/day & metformin ≥ 1.5 g/day	0.25
		Insulin/Metformin	55.3	53.6: 46.4	9.1	97.3	32	9.3	Insulin-according to blood sugar & metformin ≥ 1.5 g/day	
Voors 2022 [77]	HF	E	71	67.5: 32.5	No details	52.7	28.35	No details	10 mg/day	0.25
		Placebo	70	64.9: 35.1		56.4	29.08		Not applicable	
Wanner 2016 [78]	T2D/CKD	E	67.1	67.3: 32.7	8.07	48.4	31		10 to 25 mg/day	4
		Placebo	67.1	68.9: 31.1	8.03	48.6	30.9		Not applicable	
Yeoh 2023 [79]	HF	D	79	43: 57	6.2	40.7	32	No details	10 mg/day	0.01
		Placebo	79	48: 52	5.8	40.7	33		Not applicable	
Albulushi 2025 [80]	T2D/HF	D	65	30: 20	7.9	74	28.9	No details	10 mg/day	1
		Placebo	66	28: 22	8.2	72	29.1		Not applicable	
Bartholdy 2025 [81]	CKD	D	67	80: 30	No details				10 mg/day	0.5
		Placebo	66.9	77: 35					Not applicable	
Benedikt 2024	HF with	Er	65	91: 9	No	65	28.6		5 mg/day	1

[82]	ICD/CRT-D	Placebo	66	92: 8	details	57	28.2		Not applicable	
Burns 2025 [83]	Alzheimer's disease	D	71.7	18: 12	No details		28.3		10 mg/day	0.23
		Placebo	69.8	13: 3			28.5		Not applicable	
Cao 2025 [84]	T2D/Cardio renal syndrome	E	68.66	53: 47	7	No details	23.42		10 mg/day	1
		Standard of care	69.51	55: 45	6.98		23.4		Not applicable	
Erichsen 2025 [85]	Alzheimer's disease	E	67.4	7: 5	No details				10 mg/day	0.08
		Placebo	71.7	5: 4					Not applicable	
Ferreira 2024 [86] & Voors 2022 [77]	HF	E	69	173: 86	No details	52.5	29.5	No details	10 mg/day	0.25
		Placebo	67.5	170: 92		56	30.5		Not applicable	
Fioretto 2016 [87] (inclusive of data from 9 trials): Ferranini 2010 [88], Bailey 2010 [89], Bolinder 2012 [90], Rosenstock 2012 [91], Wilding 2012 [92], Jabbour 2014 [93], Strojek 2011 [94], Cefalu 2015 [95],	T2D (≥65 years)	D	69.9	392: 228	8	72.8	31.7	13.2	10 mg/day	2
		Placebo	69.7	401: 254	8	73.4	32	12.7	Not applicable	
	T2D (≥75 years)	D	77.1	54: 43	8.1	72.6	30.7	15.4	10 mg/day	
		Placebo	77.8	46: 31	7.8	68.6	31.9	14.9	Not applicable	

Leiter 2014 [96]										
Fujiki 2024 [97]	T2D with CVD requiring ICD/CRT-D	E	72	63: 9		57.7	25.1	No details	10 mg/day	0.5
		Placebo	70	62: 8		54	25.3		Not applicable	
Kang 2024 [98]	HF	Er	65.4	40: 23	No details	No details	24.3		5 mg/day	1
		Placebo	67.3	38: 27			25		Not applicable	
Kawanami 2025 [99]	HF	D	74	35: 21	6.2		24		10 mg/day	0.2
		Placebo	78	39: 19	5.9		23.6		Not applicable	
Kristensen 2024 [100]	T2D	E	68	11: 5	6.9	85	28	15	10 mg/day	0.08
		Placebo							Not applicable	
Lytvyn 2025 [101]	T2D/HF	Er	69.8	15: 2	8.2	63.5	30.5	No details	15 mg/day	0.25
		Placebo	69.4	13: 4	7.6	66.4	32.4		Not applicable	
McMurray 2024 [102]	HF	D	69 & 69	111: 45 & 122: 35	No details	58.5	28, 29, 29 and 28		10 mg/day	0.31
		Placebo				60			Not applicable	
Monteiro 2019a [103]	T2D	E	68.8	72: 28	8	68	30.5		10 to 25 mg/day	0.25
		Placebo	69.1	68: 32	8.03	68	30.4		Not applicable	
Monteiro 2019b [103]	T2D	E	77.9	63: 37	8	62.1	28.9		10 to 25 mg/day	
		Placebo	78.1	74: 26	7.94	62.1	29.6		Not applicable	
Nantsupawat 2024 [104]	AHRE	D	70	19: 8	5.8	72.4	23.1		10 mg/day	0.25
		Placebo	69.7	12: 15	5.8	62.7	23.2		Not applicable	

Nielsen 2024 [105]	T2D	E	65	No details			28		10 mg/day	
		Placebo							Not applicable	
Pastore 2024 [106]	HF	D	68	36: 8		67	26.5		10 mg/day	0.08
		Placebo	68	37: 7		68	28		Not applicable	
Pratley 2020 [107] (data from 7 trials from 8 articles): Terra 2017 [108], Rosenstock 2018 [109], Pratley 2018 [110], Hollander 2018 [111], Dagogo-Jack 2018 [112], Grunberger 2018 [113], Miller 2018 [114], Aronson 2018 [115]	T2D	Er	70	226: 223	8.1	71.7	30.3	11.5	5 mg/day	0.05
		Er	69.9	191: 197	8	72.2	31	10.7	15 mg/day	
		Placebo/Sitagliptin/Glipizide	70.1	200: 227	8.1	70.2	30.5	11.1	Not applicable for the placebo	
Reutens 2025 [116]	T2D	E	69	10: 5	7.9	No details	30.9	No details	25 mg/day	0.25
		Placebo	66.9	11: 6	8.1		31.4		Not applicable	
Shigeno 2024 [117]	T2D	L	69.4	7: 3	7.6		26.1	6.1	2.5 mg/day	0.92
		Metformin	67.7	8: 4	7.4		23.4	10.8	500 mg/day	
Sinclair 2014	T2D	C	70	81: 78	7.9	77.3	30.8	10.5	100 mg/day	0.5

[118] (data from 4 trials): Stenlof 2013 [119], Bode 2013 [120], Lavalle-Gonzalez 2013 [121], Wilding 2013 [122]		C	69.1	77: 72	7.9	71.4	30.5	10.8	300 mg/day	
		Placebo	68.7	71: 66	7.8	75.9	30.8	10.2	Not applicable	
Sinclair 2016 [123] (data from 7 trials: Stenlof 2013 [119], Lavalle-Gonzalez 2013 [121], Wilding 2013 [122], Forst 2014 [124], Bode 2013 [120], Cefalu 2013 [95], Yale 2014 [125]	T2D/CKD	C	77.5	103: 60	7.9	62.7	29.7	16	100 mg/day	0.5
		C	77.4	117: 55	7.8	63.8	30	16.3	300 mg/day	
		Placebo	77.6	96: 59	7.9	62.5	30.2	16.8	Not applicable	
Siqueira 2005 [126] & Solomon 2022 [127]	HF	D	71.8	56.4: 43.6	No details	61	29.8	No details	10 mg/day	Median (2.3)
		Placebo	58.3	73.3: 26.4		61	29.9		Not applicable	
Yoshihara 2023 [128]	T2D/HF	D	72.1	104: 40	6.9	66.9	25.3		10 mg/day	2
		Placebo	72.2	99: 42	6.9	64.6	24.8		Not applicable	

Zhou 2024 [129]	T2D/CRS	E	68.18	32: 18	7.35	No details	23.18		10 mg/day	0.25
		Control	71.21	36: 22	7.25		23.38		Not applicable	
Jurgens 2021 [130]	T2D	E	66	76: 24	7.7	79.5	31.9	14	25 mg/day	0.25
		Placebo	67	84: 16	7.5	79.3	29.8	15	Not applicable	
Nassif 2021 [131]	HF	D	69	43.2: 56.8	6	56	35.1	No details	10 mg/day	0.25
		Placebo	71	43.2: 56.8	6.2	54	34.6		Not applicable	
Yabe 2023 [132]	T2D	E	74.2	75: 25	7.6	81.9	25.7	12.4	10 mg/day	1
		Placebo	74	69.8: 30.2	7.6	79.1	25.4	11.8	Not applicable	
Antlanger 2022 [133]	T2D/CKD	E	71	91.6: 8.4	6.9	33	31	No details	10 mg/day	0.25
		Placebo	69	58.3: 41.7	6.8	37	28		Not applicable	
Veelen 2023 [134]	Pre-diabetic	D & Placebo	66.3	57.1: 42.9	5.5	99	30		D- 10 mg/day	0.04

T2D-Type 2 diabetes; T1D-Type 1 diabetes; HF-Heart failure; ACS-acute coronary syndrome; CKD-chronic kidney disease; COVID-19-Coronavirus disease 2019; PAH-Pulmonary arterial hypertension; ICD/CRT-D-implantable cardioverter-defibrillator or cardiac resynchronization therapy-defibrillator; AHRE: Atrial high-rate episodes; and CRS: Cardiorenal syndrome.

E-Empagliflozin; D-Dapagliflozin; C-Canagliflozin; Er-Ertugliflozin; L-Licogliflozin; Lu-Luseogliflozin; and S-Sotagliflozin.

Electronic Supplementary Table S4. Leave-one-out sensitivity analysis for SGLT2is compared to non-SGLT2i group for mortality.

Excluded study	Pooled OR	LCI 95%	HCI 95%
Albulushi 2025	0.84	0.75	0.93
Cao 2025	0.84	0.75	0.93
Fioretto 2016a	0.85	0.78	0.92
Fioretto 2016b	0.83	0.74	0.93
Kang 2024	0.84	0.76	0.93
Pratley 2020	0.84	0.76	0.92
Sinclair 2014	0.84	0.76	0.93
Sinclair 2016	0.85	0.77	0.93
Yoshihara 2023	0.85	0.78	0.94
Nassif 2021	0.83	0.75	0.93
Benedikt 2024	0.84	0.76	0.93
Monteiro 2019a	0.86	0.78	0.95
Monteiro 2019b	0.86	0.78	0.95
EMPEROR studies	0.80	0.73	0.89
Bhatt 2021a	0.82	0.73	0.92
SCORED trials	0.82	0.72	0.92
Cherney 2021	0.84	0.75	0.93
Cherney 2023	0.83	0.74	0.93
Dagogo-Jack 2021	0.83	0.74	0.93
Damman 2020	0.83	0.74	0.93
Dekkers 2018	0.84	0.75	0.93
Haneda 2016	0.83	0.74	0.93
Kohan 2014	0.84	0.75	0.93
McMurray 2019 & McMurray 2021	0.83	0.73	0.94
Provenzano 2022	0.83	0.75	0.93
Refardt 2020	0.83	0.75	0.93
Schulze 2022	0.83	0.75	0.93
Tanaka 2020	0.84	0.75	0.93
Voors 2022	0.84	0.76	0.94
Wanner 2016	0.83	0.74	0.93

Electronic Supplementary Table S5. Leave-one-out sensitivity analysis for SGLT2is compared to non-SGLT2i group for ARF.

Excluded study	Pooled OR	LCI 95%	HCI 95%
Cao 2025	0.86	0.79	0.94
Ferreira 2024	0.87	0.80	0.95
Nassif 2021	0.86	0.79	0.94
Monteiro 2019a	0.87	0.80	0.95
Monteiro 2019b	0.87	0.80	0.95
Allegretti 2019	0.86	0.79	0.94
EMPEROR studies	0.82	0.73	0.92
Bhatt 2021a	0.86	0.79	0.94
SCORED trials	0.87	0.78	0.96
Dagogo-Jack 2021	0.87	0.79	0.94
Damman 2020	0.86	0.79	0.94
Dekkers 2018	0.87	0.80	0.94
Fioretto 2018	0.86	0.79	0.94
Ge 2023	0.86	0.79	0.94
Kohan 2014	0.86	0.79	0.94
Lee 2021	0.86	0.79	0.94
Provenzano 2022	0.86	0.79	0.94
Schulze 2022	0.87	0.80	0.94
Wanner 2016	0.87	0.80	0.96

Electronic Supplementary Table S6. Leave-one-out sensitivity analysis for SGLT2is compared to non-SGLT2i group for diarrhea.

Excluded study	Pooled OR	LCI 95%	HCI 95%
Burns 2025	1.38	1.10	1.74
Nielsen 2024	1.32	0.98	1.76
Yabe 2023	1.44	1.25	1.65
Kario 2018	1.35	1.04	1.76
Scott 2018	1.45	1.27	1.67
Bhatt 2021a	1.20	0.85	1.69
SCORED trials	1.14	0.73	1.76
Cherney 2021	1.28	0.95	1.73
Cherney 2023	1.25	0.88	1.77
Damman 2020	1.42	1.20	1.68
de Boer 2020	1.31	0.98	1.75
Aart-van der Beek 2023	1.35	1.04	1.76

Electronic Supplementary Table S7. Leave-one-out sensitivity analysis for SGLT2is compared to non-SGLT2i group for genital infections.

Excluded study	Pooled OR	LCI 95%	HCI 95%
Cao 2025	3.35	2.70	4.15
Erichsen 2025	3.38	2.72	4.18
Fioretto 2016a	3.15	2.52	3.93
Fioretto 2016b	3.30	2.66	4.09
Fujiki 2024	3.36	2.71	4.16
Kristensen 2024	3.31	2.68	4.11
Pratley 2020	3.25	2.61	4.04
Sinclair 2014	3.29	2.66	4.08
Sinclair 2016	3.27	2.63	4.05
Jurgens 2023	3.31	2.67	4.10
Scott 2018	3.32	2.68	4.11
Benedikt 2024	3.33	2.69	4.13
Monteiro 2019a	3.42	2.71	4.33
Monteiro 2019b	3.28	2.64	4.07
Abraham 2021	3.33	2.68	4.13
Allegretti 2019	3.30	2.67	4.09
EMPEROR studies	3.51	2.72	4.54
Bhatt 2021a	3.31	2.67	4.10
Cheng 2022	3.28	2.65	4.07
Cherney 2021	3.34	2.69	4.13
Cherney 2023	3.35	2.70	4.16
Dagogo-Jack 2021	3.36	2.69	4.21
de Boer 2020	3.34	2.70	4.14
Dekkers 2018	3.33	2.68	4.12
Fioretto 2018	3.36	2.71	4.17
Fu 2023	3.33	2.68	4.12
Ge 2023	3.32	2.68	4.11
Haneda 2016	3.33	2.69	4.12
Kohan 2014	3.35	2.69	4.16
Lee 2021	3.38	2.72	4.20
Mansouri 2023	3.33	2.68	4.12
Wanner 2016	3.33	2.66	4.17

Electronic Supplementary Table S8. Leave-one-out sensitivity analysis for SGLT2is compared to non-SGLT2i group for volume depletion.

Excluded study	Pooled OR	LCI 95%	HCI 95%
Ferreira 2024	1.17	1.08	1.27
Fioretto 2016a	1.17	1.08	1.27
Fioretto 2016b	1.18	1.08	1.27
Fujiki 2024	1.18	1.09	1.27
Pratley 2020	1.17	1.08	1.27
Sinclair 2014	1.17	1.08	1.27
Sinclair 2016	1.17	1.08	1.27
Siqueira 2025 & Solomon 2022	1.17	1.08	1.27
Nassif 2021	1.17	1.08	1.27
Monteiro 2019a	1.19	1.10	1.29
Monteiro 2019b	1.17	1.08	1.27
Abraham 2021a	1.18	1.09	1.28
EMPEROR studies	1.16	1.05	1.29
Bhatt 2021a	1.18	1.09	1.28
SCORED trials	1.14	1.05	1.25
Cheng 2022	1.17	1.08	1.27
Cherney 2021	1.18	1.09	1.27
Cherney 2023	1.17	1.08	1.27
Dagogo-Jack 2021	1.18	1.09	1.28
Dekkers 2018	1.18	1.09	1.27
Fu 2023	1.18	1.09	1.27
Haneda 2016	1.18	1.08	1.27
Kohan 2014	1.17	1.08	1.27
McMurray 2019 & McMurray 2021	1.19	1.09	1.29
Tanaka 2020	1.17	1.08	1.27
Wanner 2016	1.20	1.10	1.30
Yeoh 2023	1.18	1.09	1.28

Electronic Supplementary Table S9. Leave-one-out sensitivity analysis for SGLT2is compared to non-SGLT2i group for polyuria.

Excluded study	Pooled OR	LCI 95%	HCI 95%
Reutens 2025	5.37	2.38	12.16
Jurgens 2023	4.19	1.78	9.87
Benedikt 2024	5.13	2.27	11.62
Allegretti 2019	8.36	2.36	29.59
Kario 2019	5.47	2.41	12.38
Mansouri 2023	5.10	2.24	11.59

Electronic Supplementary Table S10. Leave-one-out sensitivity analysis for SGLT2is compared to non-SGLT2i group for serious adverse events.

Excluded study	Pooled OR	LCI 95%	HCI 95%
Pratley 2020	0.83	0.78	0.89
Sinclair 2014	0.83	0.79	0.88
Sinclair 2016	0.84	0.79	0.90
Siqueira 2025 & Solomon 2022	0.84	0.78	0.90
Jurgens 2023	0.84	0.78	0.89
Nassif 2021	0.83	0.78	0.88
Yabe 2023	0.84	0.78	0.89
Scott 2018	0.83	0.78	0.89
Bartholdy 2025	0.83	0.78	0.89
Monteiro 2019a	0.84	0.79	0.90
Monteiro 2019b	0.84	0.79	0.90
Abraham 2021a	0.84	0.79	0.90
Allegretti 2019	0.83	0.78	0.89
EMPEROR studies	0.84	0.78	0.91
Bhatt 2021a	0.83	0.78	0.89
SCORED trials	0.82	0.77	0.89
Cherney 2021	0.84	0.78	0.89
Cherney 2023	0.84	0.78	0.89
Dagogo-Jack 2021	0.83	0.77	0.89
Damman 2020	0.84	0.78	0.90
Dekkers 2018	0.84	0.78	0.90
Fioretto 2018	0.84	0.78	0.90
Haneda 2016	0.84	0.78	0.89
Kohan 2014	0.84	0.78	0.89
McMurray 2019 & McMurray 2021	0.84	0.78	0.90
Provenzano 2022	0.84	0.78	0.89
Refardt 2020	0.84	0.78	0.89
Aart-van der Beek 2023	0.84	0.78	0.89
Voors 2022	0.84	0.79	0.90
Wanner 2016	0.84	0.79	0.90

Electronic Supplementary Table S11. Leave-one-out sensitivity analysis for SGLT2is compared to non-SGLT2i group for myocardial infarction.

Excluded study	Pooled OR	LCI 95%	HCI 95%
Cao 2025	0.74	0.61	0.90
Lytvyn 2025	0.74	0.61	0.89
Nassif 2021	0.74	0.61	0.90
Monteiro 2019a	0.71	0.56	0.89
Monteiro 2019b	0.76	0.62	0.93
SCORED trials	0.75	0.56	1.00
Cherney 2021	0.75	0.62	0.91
Lee 2021	0.73	0.61	0.89

Electronic Supplementary Figure S1. Forest plot for the risk of amputation with SGLT2i compared to non-SGLT2i.

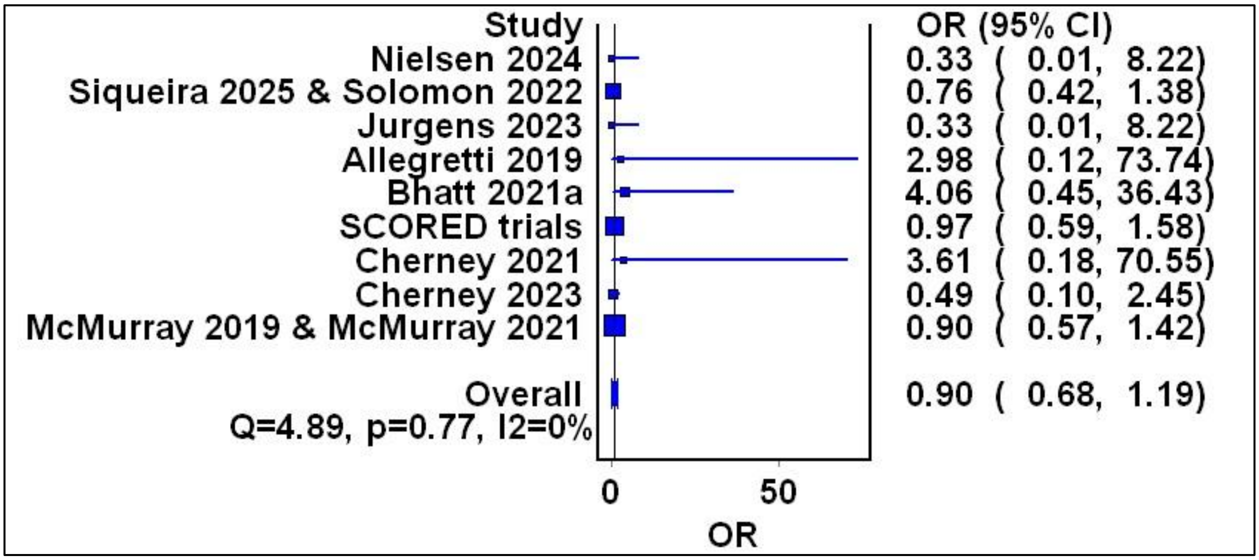

Electronic Supplementary Figure S2. Forest plot for the risk of constipation with SGLT2i compared to non-SGLT2i.

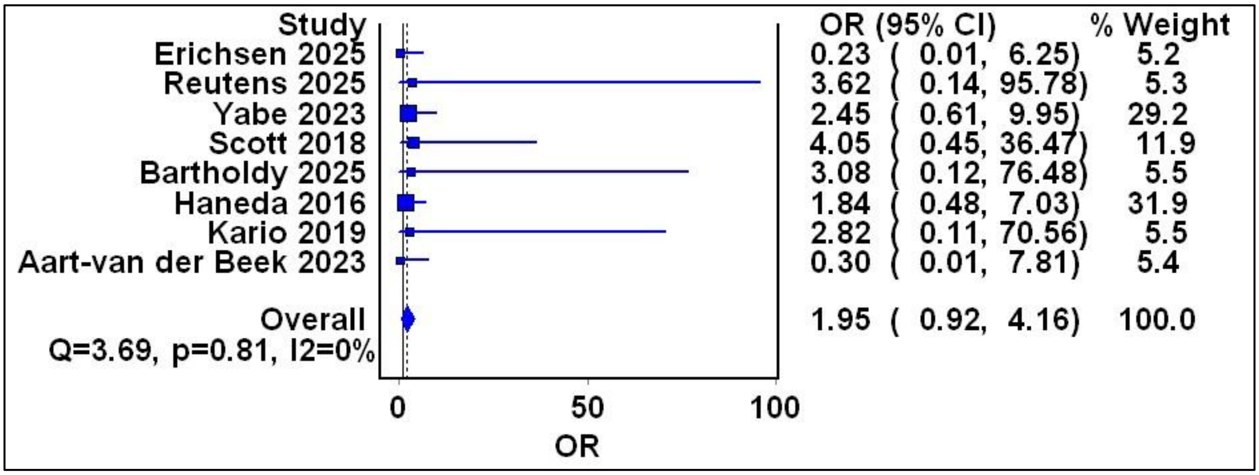

Electronic Supplementary Figure S3. Forest plot for the risk of therapy discontinuation with SGLT2i compared to non-SGLT2i.

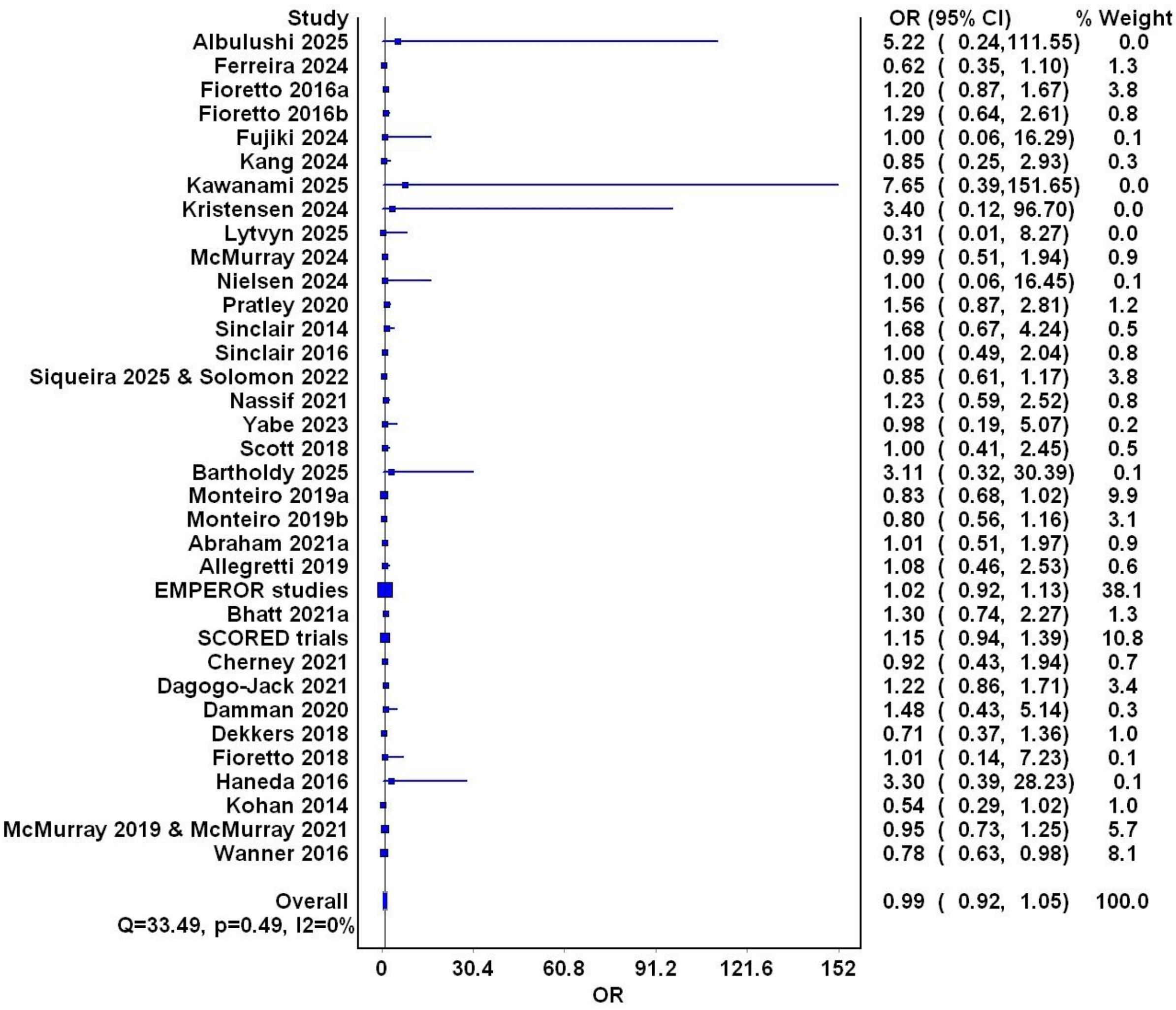

Electronic Supplementary Figure S4. Forest plot for the risk of diabetic ketoacidosis with SGLT2i compared to non-SGLT2i.

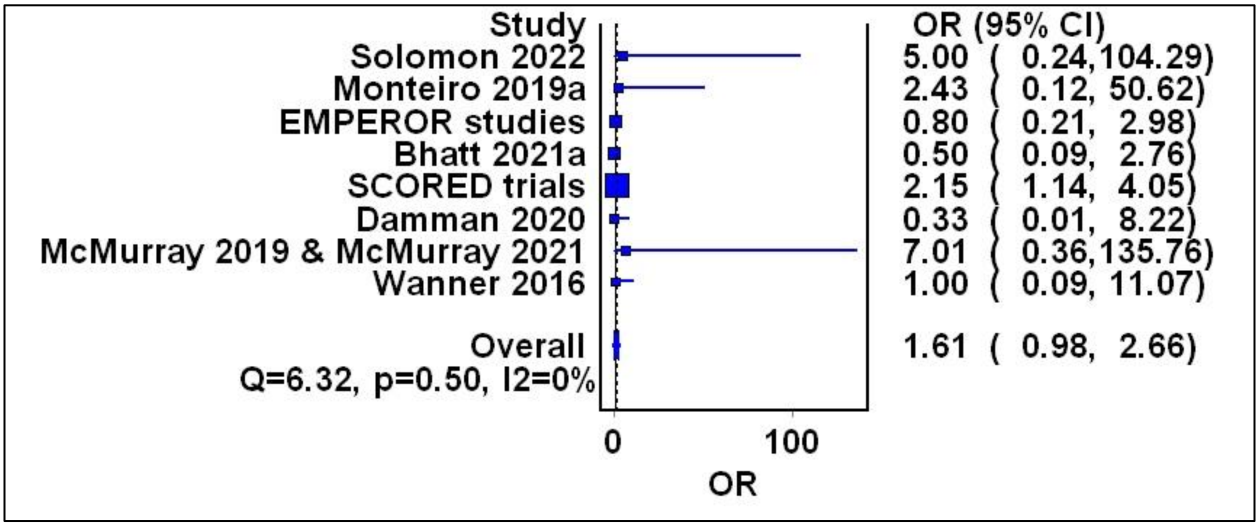

Electronic Supplementary Figure S5. Forest plot for the risk of fracture with SGLT2i compared to non-SGLT2i.

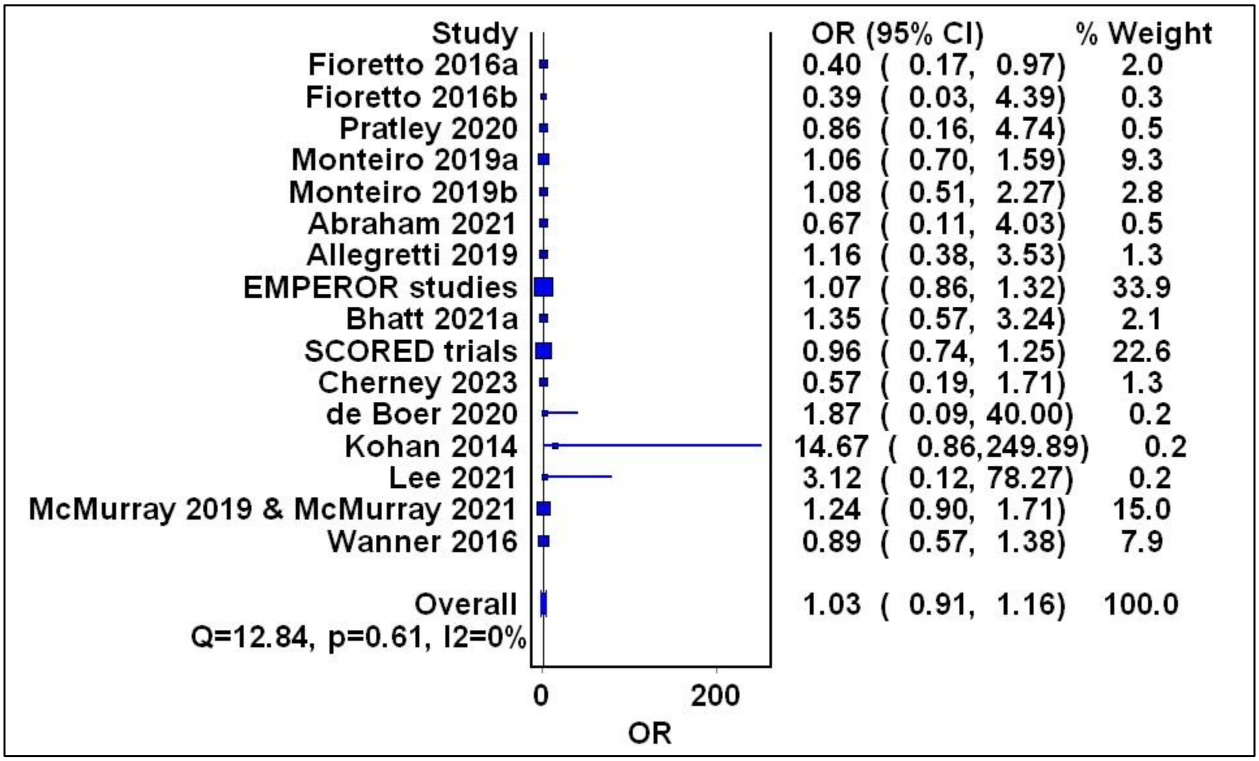

Electronic Supplementary Figure S6. Forest plot for the risk of hepatic dysfunction with SGLT2i compared to non-SGLT2i.

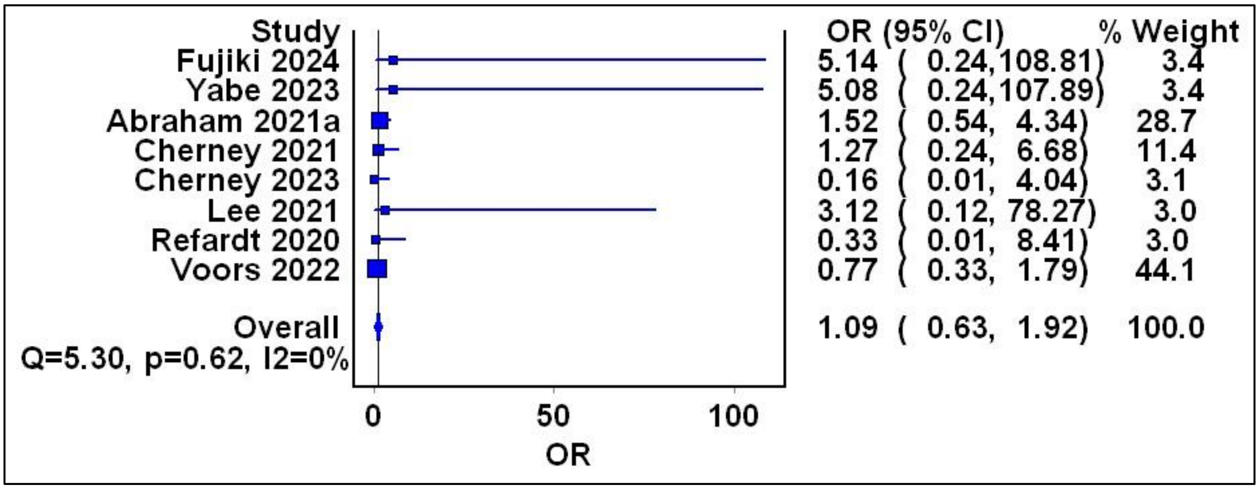

Electronic Supplementary Figure S7. Forest plot for the risk of hyperkalemia with SGLT2i compared to non-SGLT2i.

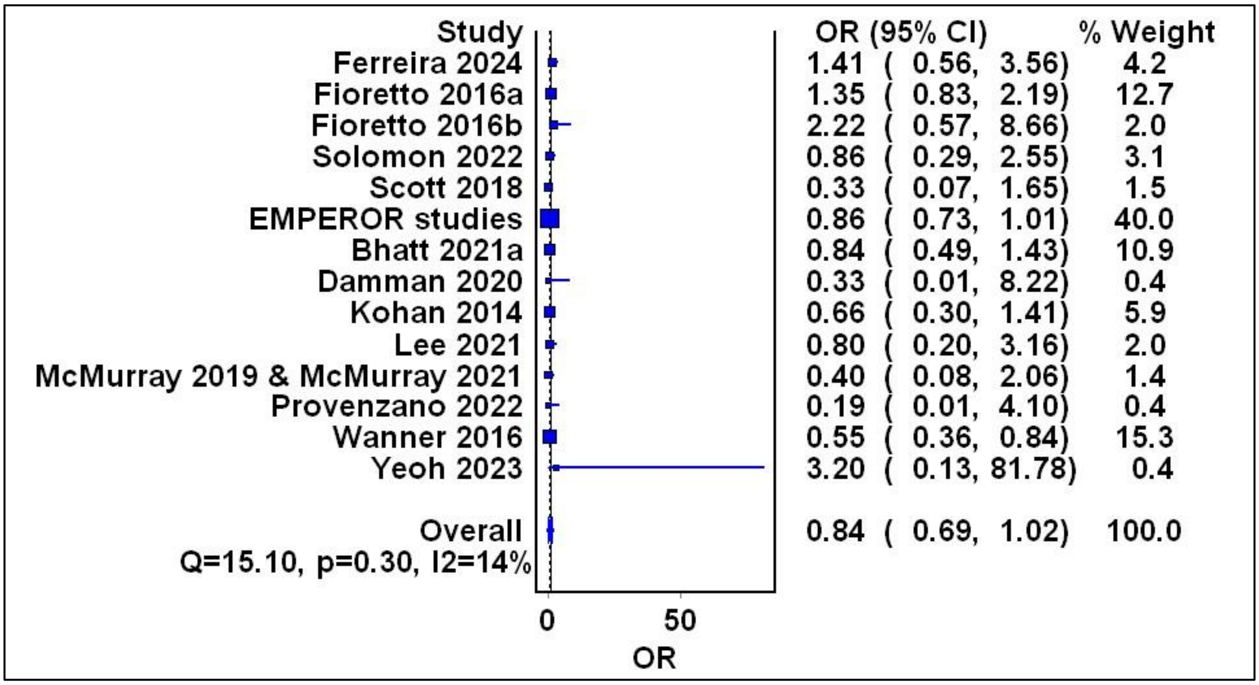

Electronic Supplementary Figure S8. Forest plot for the risk of hypotension with SGLT2i compared to non-SGLT2i.

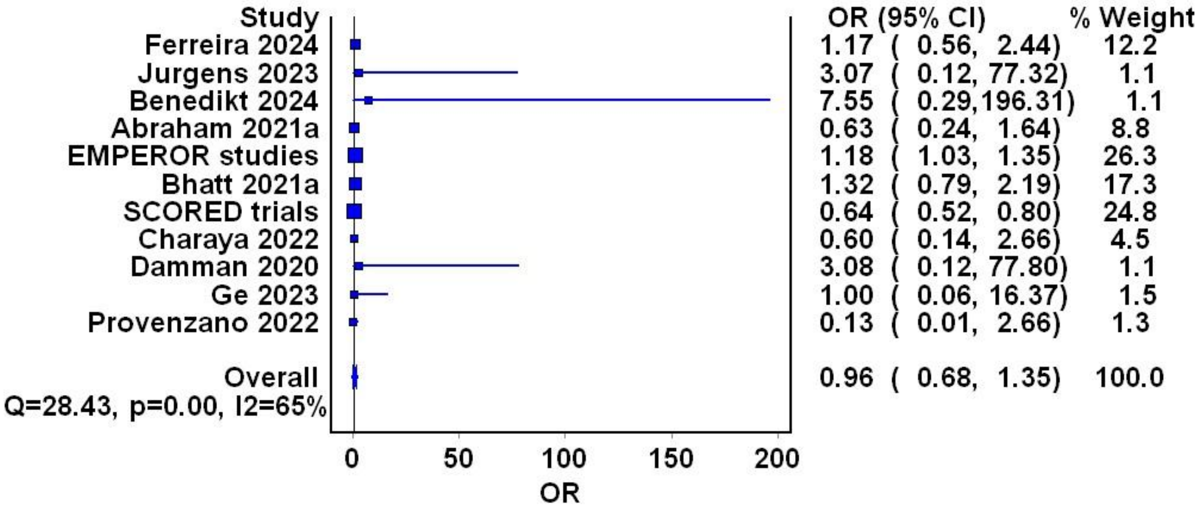

Electronic Supplementary Figure S9. Forest plot for the risk of malignancies with SGLT2i compared to non-SGLT2i.

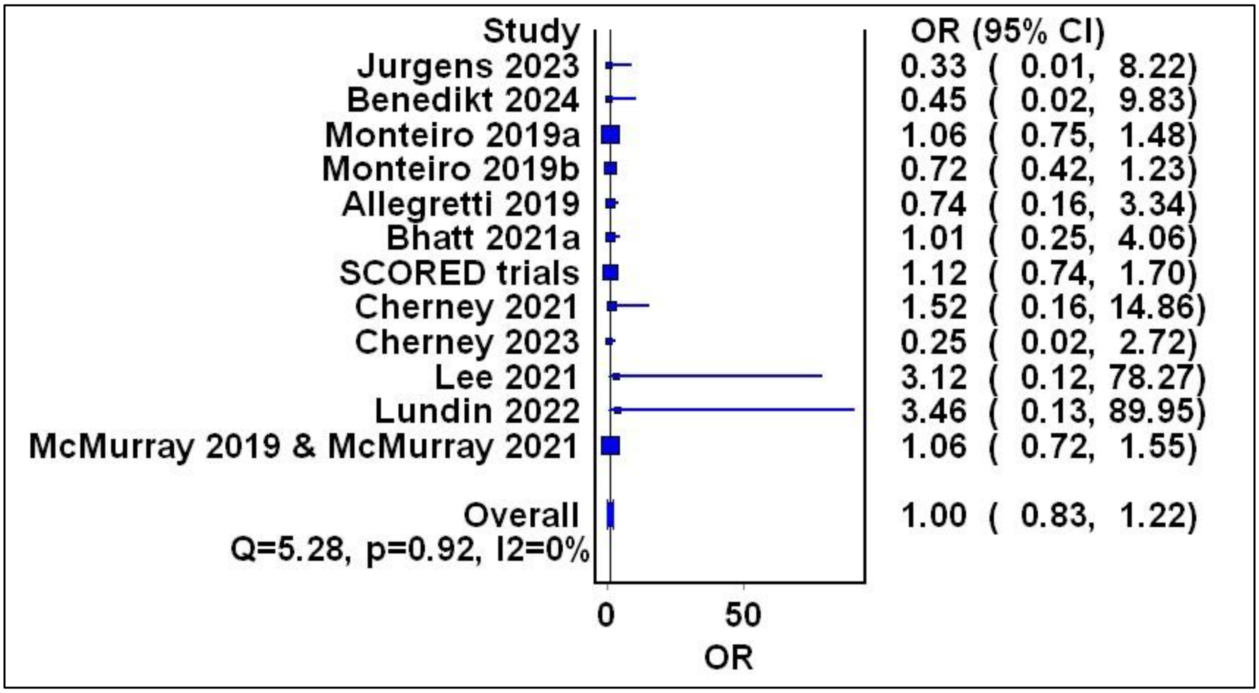

Electronic Supplementary Figure S10. Forest plot for the risk of stroke with SGLT2i compared to non-SGLT2i.

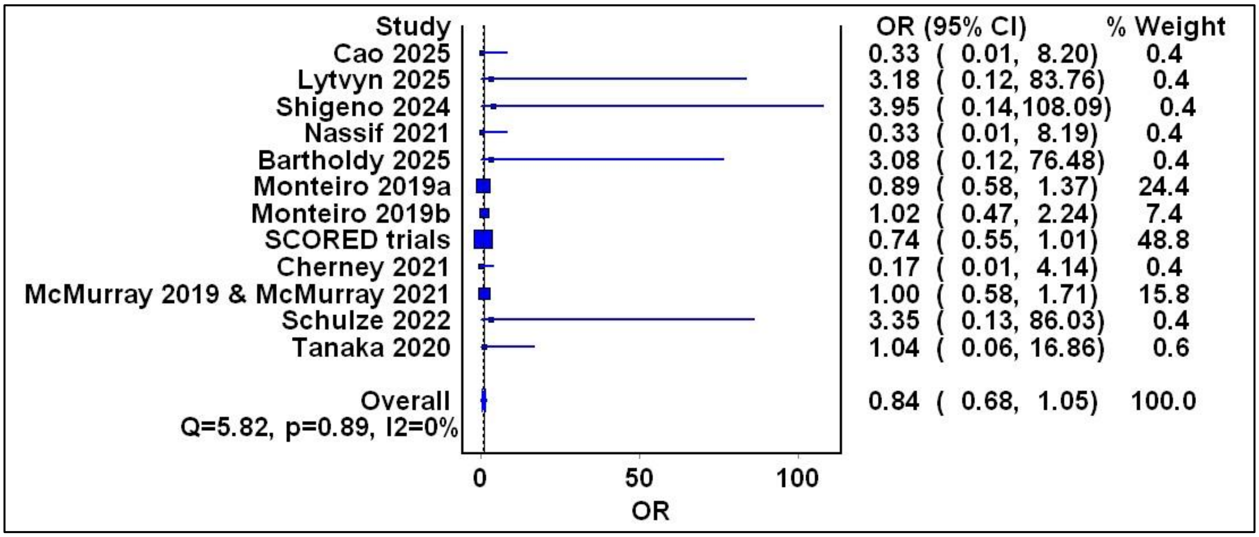

Electronic Supplementary Figure S11. Forest plot for the risk of hypoglycemia with SGLT2i compared to non-SGLT2i.

Study	Experimental Events	Experimental Total	Control Events	Control Total	OR	95% CI	Weight
Cao 2025	1	100	0	100	3.03	[0.12; 75.28]	0.1%
Fioretto 2016a	2	620	0	655	5.30	[0.25; 110.60]	0.1%
Fujiki 2024	0	75	1	75	0.33	[0.01; 8.20]	0.1%
Kawanami 2025	0	56	1	58	0.34	[0.01; 8.50]	0.0%
Nielsen 2024	1	49	2	49	0.49	[0.04; 5.58]	0.1%
Pratley 2020	14	235	2	120	3.74	[0.84; 16.72]	0.2%
Sinclair 2016	120	335	50	155	1.17	[0.78; 1.76]	3.2%
Siqueira 2025 & Solomon 2022	1	572	1	579	1.01	[0.06; 16.22]	0.1%
Yabe 2023	1	65	1	65	1.00	[0.06; 16.34]	0.1%
Scott 2018	21	306	22	307	0.95	[0.51; 1.77]	1.3%
Monteiro 2019a	480	1667	246	808	0.92	[0.77; 1.11]	15.4%
Monteiro 2019b	117	424	62	228	1.02	[0.71; 1.46]	4.0%
Abraham 2021	2	309	1	311	2.02	[0.18; 22.39]	0.1%
Allegretti 2019	39	157	38	155	1.02	[0.61; 1.70]	2.0%
EMPEROR studies	100	4858	106	4852	0.94	[0.71; 1.24]	6.8%
Bhatt 2021a	26	605	17	611	1.57	[0.84; 2.92]	1.3%
SCORED trials	406	5291	416	5286	0.97	[0.84; 1.12]	25.6%
Cheng 2022	9	62	6	62	1.58	[0.53; 4.76]	0.4%
Cherney 2021	73	184	38	93	0.95	[0.57; 1.58]	2.0%
Cherney 2023	204	527	99	260	1.03	[0.76; 1.39]	5.6%
Dagogo-Jack 2021	425	1177	224	597	0.94	[0.77; 1.15]	12.5%
de Boer 2020	3	91	1	33	1.09	[0.11; 10.87]	0.1%
Dekkers 2018	54	151	26	69	0.92	[0.51; 1.66]	1.5%
Fioretto 2018	20	160	22	161	0.90	[0.47; 1.73]	1.2%
Fu 2023	1	30	0	30	3.10	[0.12; 79.23]	0.0%
Ge 2023	2	60	0	60	5.17	[0.24; 110.01]	0.1%
Haneda 2016	3	95	3	50	0.51	[0.10; 2.63]	0.2%
Kohan 2014	71	168	43	84	0.70	[0.41; 1.18]	1.9%
McMurray 2019 & McMurray 2021	14	2368	28	2368	0.50	[0.26; 0.95]	1.3%
Tanaka 2020	0	118	2	123	0.21	[0.01; 4.32]	0.1%
Aart-van der Beek 2023	1	18	1	17	0.94	[0.05; 16.35]	0.1%
Voors 2022	5	260	4	264	1.27	[0.34; 4.80]	0.3%
Wanner 2016	391	1212	233	607	0.76	[0.62; 0.94]	12.6%
**Random effects model**		**22405**		**19292**	**0.94**	**[0.87; 1.01]**	**100.0%**

Heterogeneity: $I^2 = 0.0\%$, $\tau^2 = 0$, $p = 0.8303$

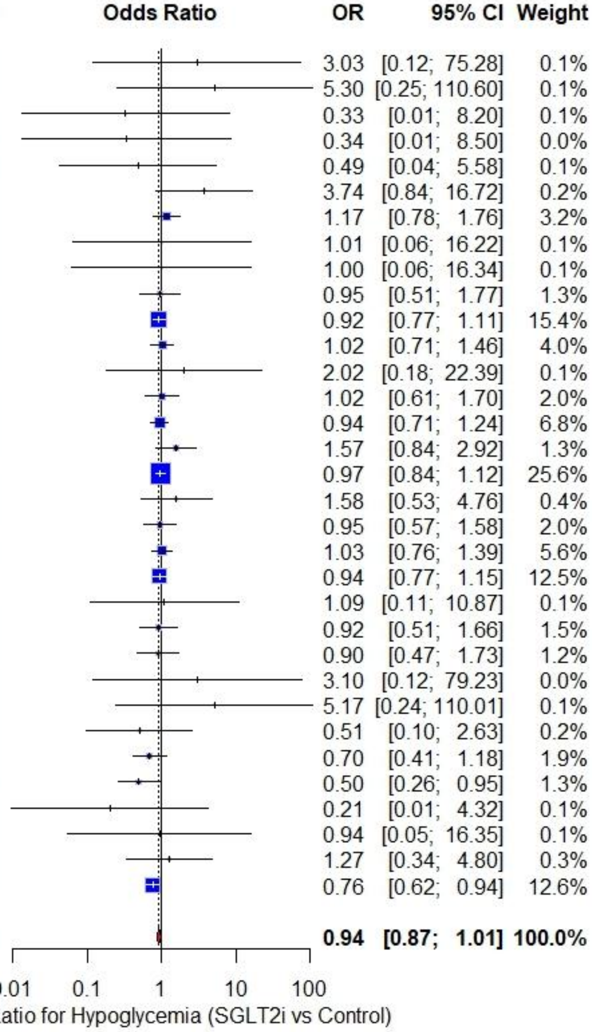

Electronic Supplementary Figure S12. Forest plot for the risk of UTI with SGLT2i compared to non-SGLT2i.

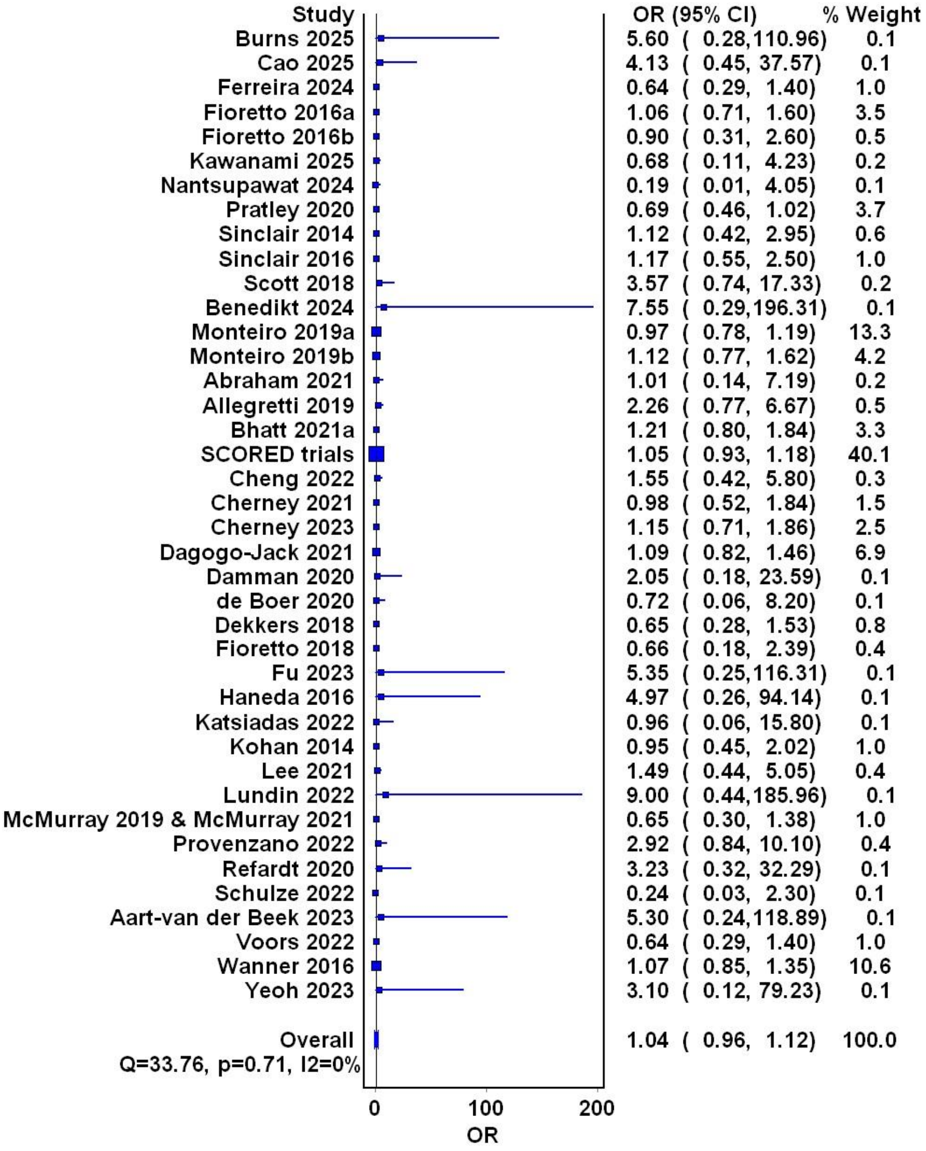

Electronic Supplementary Figure S13. Forest plot for mixed treatment comparison pooled estimates for the risk of constipation with various doses of SGLT2is compared to non-SGLT2i group.

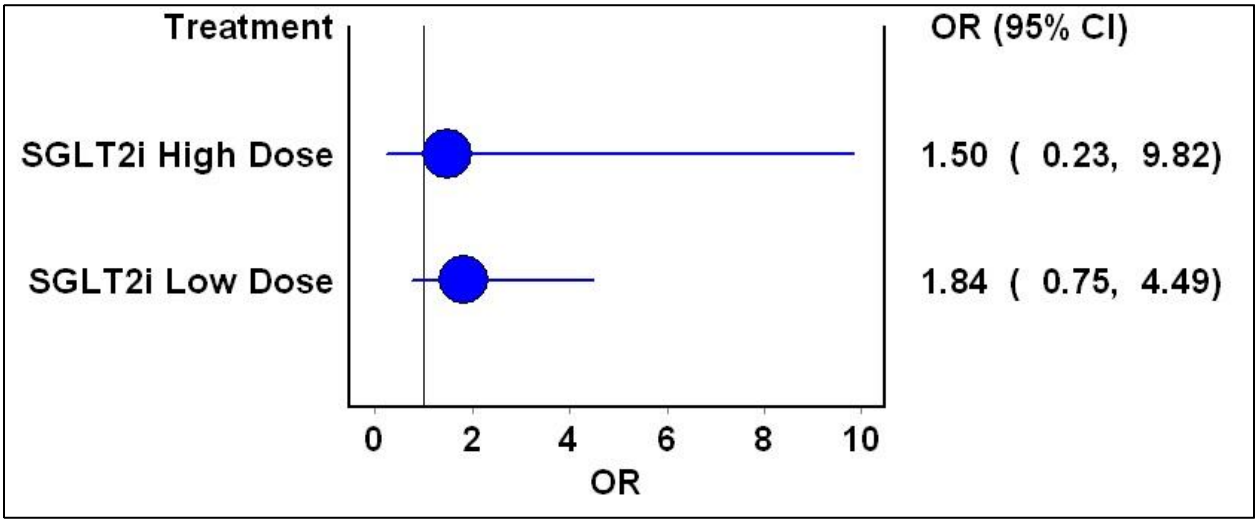

Electronic Supplementary Figure S14. Forest plot for mixed treatment comparison pooled estimates for the risk of therapy discontinuation with various doses of SGLT2is compared to non-SGLT2i group.

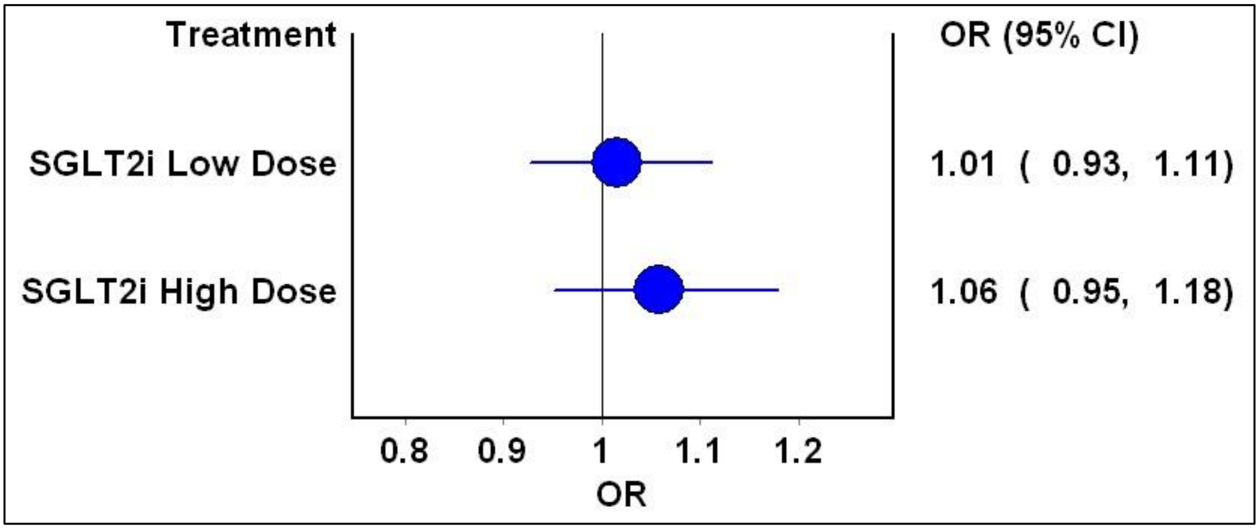

Electronic Supplementary Figure S15. Forest plot for mixed treatment comparison pooled estimates for the risk of diabetic ketoacidosis with various doses of SGLT2is compared to non-SGLT2i group.

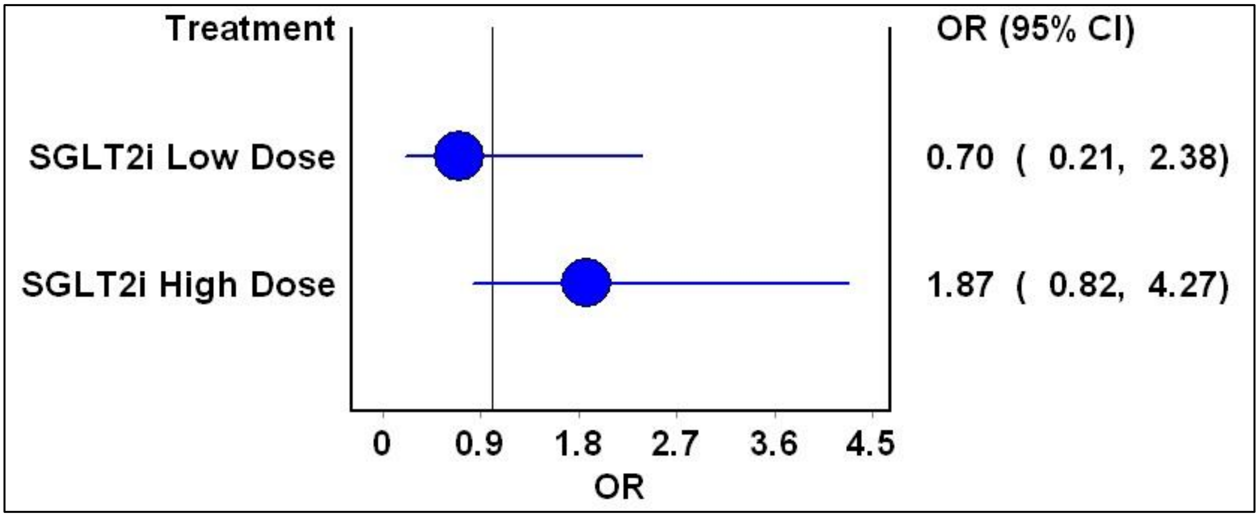

Electronic Supplementary Figure S16. Forest plot for mixed treatment comparison pooled estimates for the risk of fracture with various doses of SGLT2is compared to non-SGLT2i group.

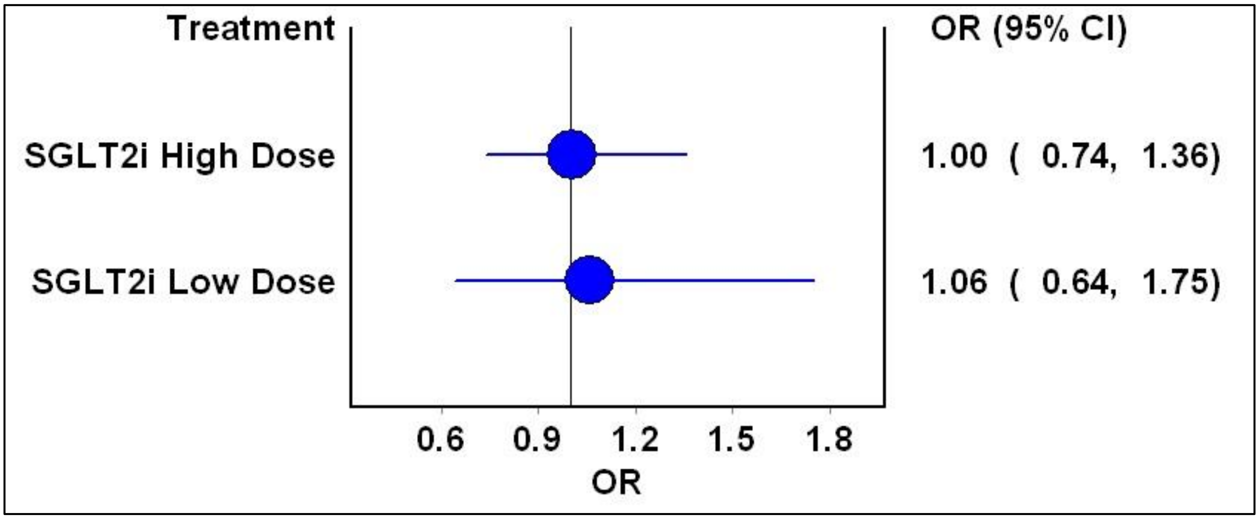

Electronic Supplementary Figure S17. Forest plot for mixed treatment comparison pooled estimates for the risk of hepatic dysfunction with various doses of SGLT2is compared to non-SGLT2i group.

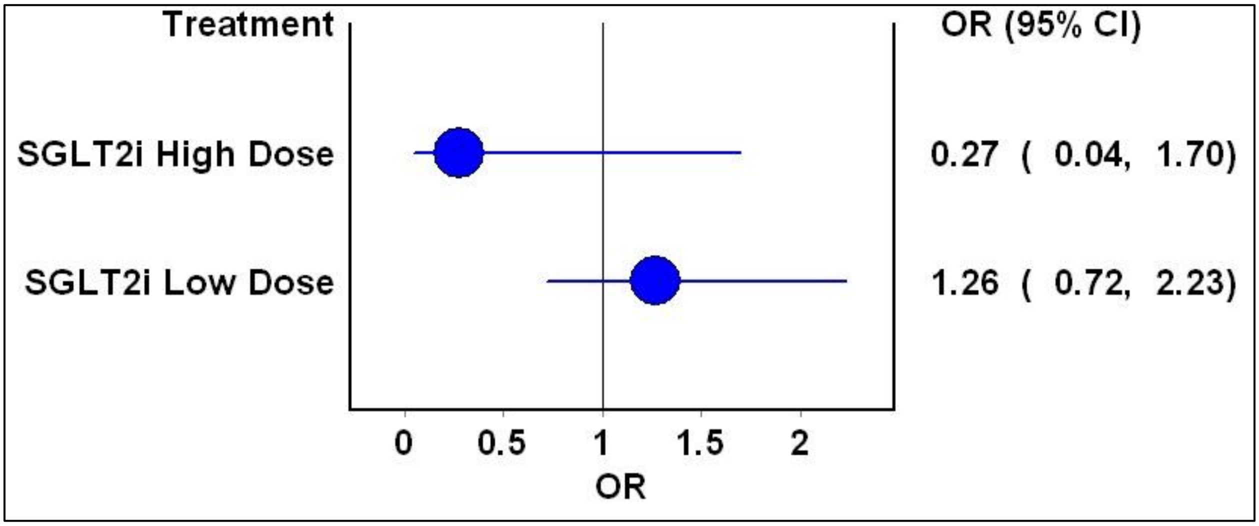

Electronic Supplementary Figure S18. Forest plot for mixed treatment comparison pooled estimates for the risk of hyperkalemia with various doses of SGLT2is compared to non-SGLT2i group.

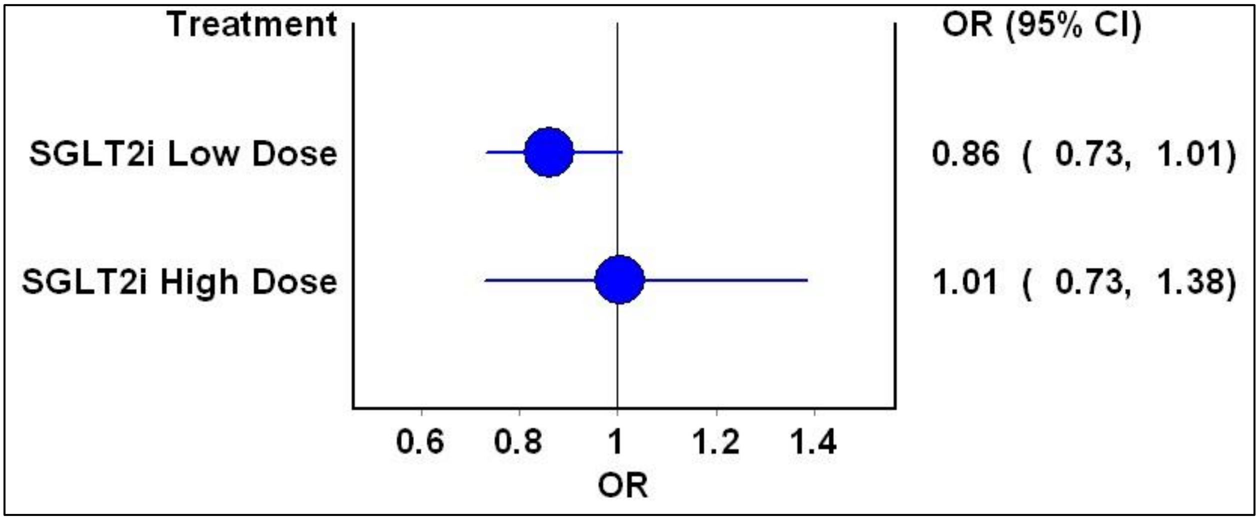

Electronic Supplementary Figure S19. Forest plot for mixed treatment comparison pooled estimates for the risk of hypoglycemia with various doses of SGLT2is compared to non-SGLT2i group.

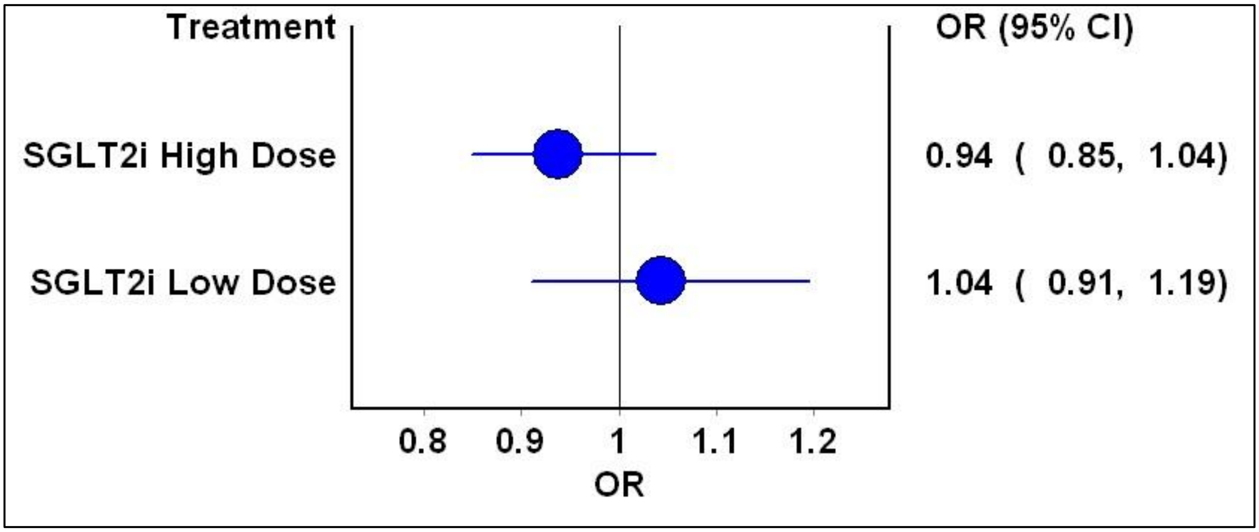

Electronic Supplementary Figure S20. Forest plot for mixed treatment comparison pooled estimates for the risk of malignancies with various doses of SGLT2is compared to non-SGLT2i group.

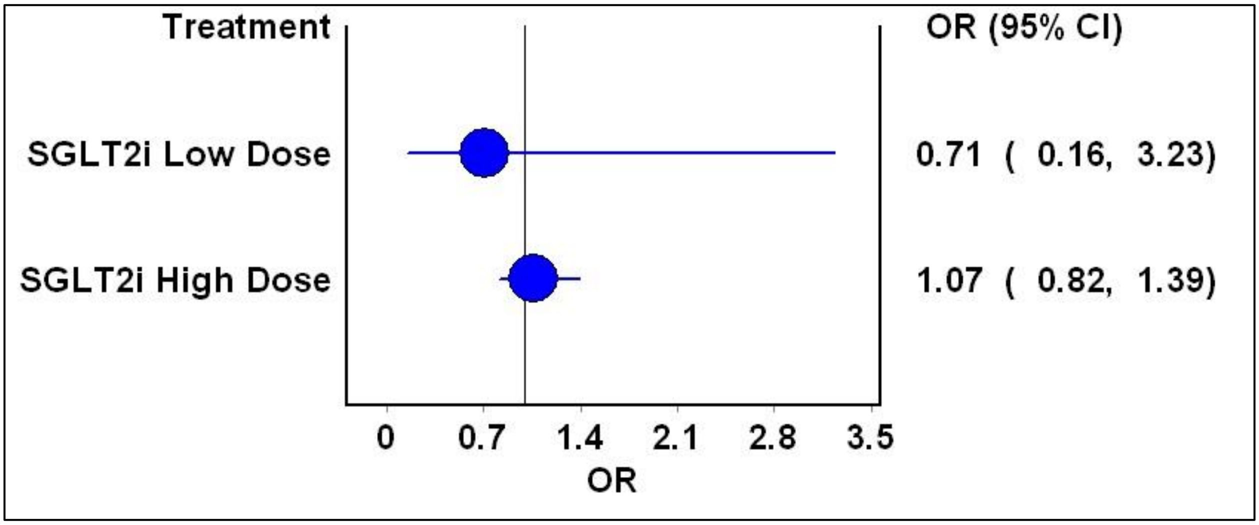

Electronic Supplementary Figure S21. Forest plot for mixed treatment comparison pooled estimates for the risk of stroke with various doses of SGLT2is compared to non-SGLT2i group.

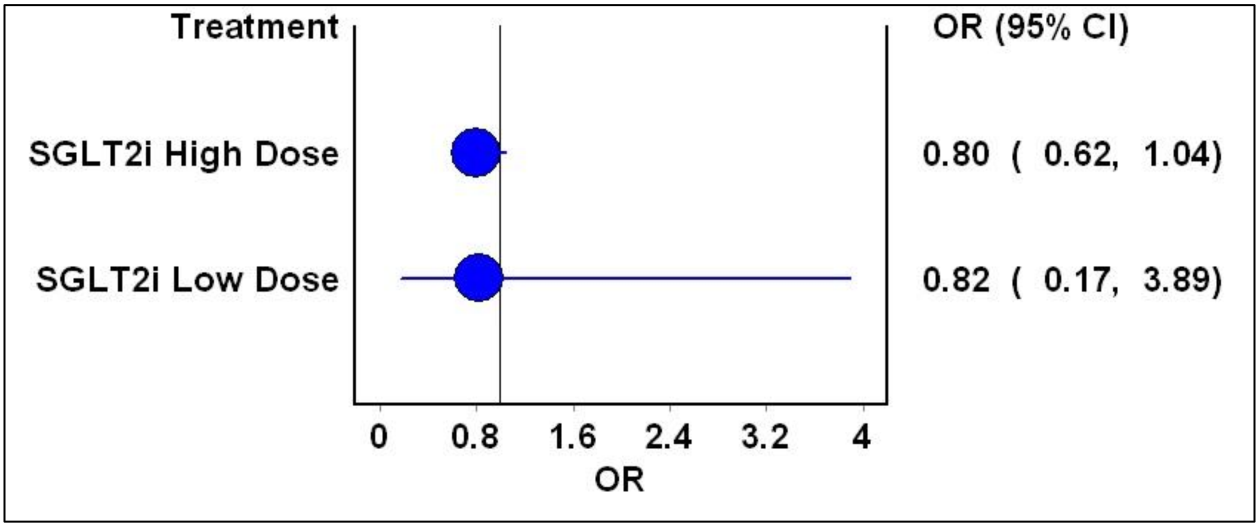

Electronic Supplementary Figure S22. Forest plot for mixed treatment comparison pooled estimates for the risk of urinary tract infections with various doses of SGLT2is compared to non-SGLT2i group.

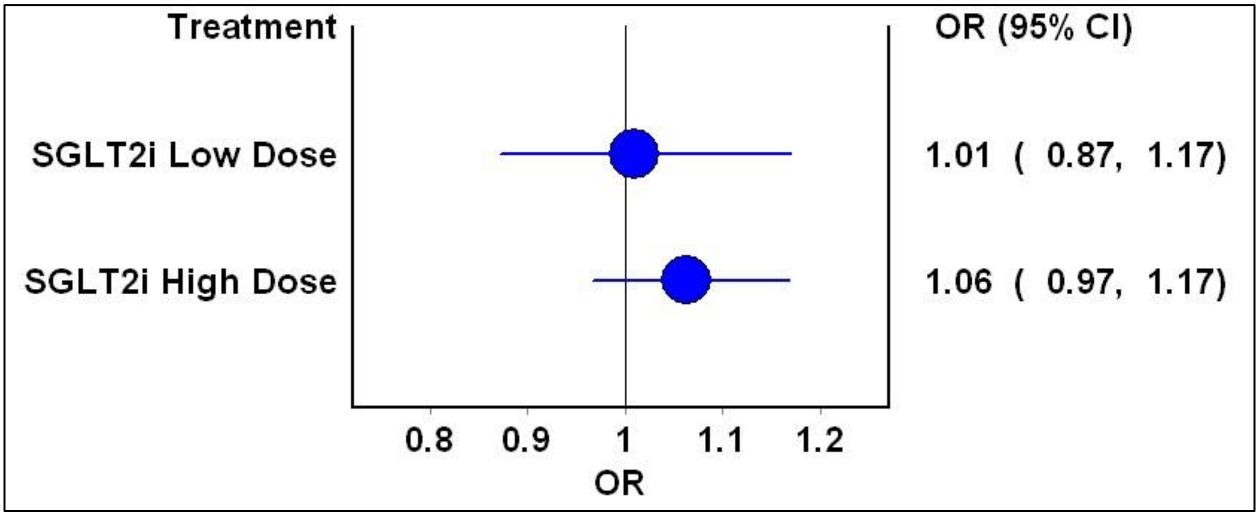

Electronic Supplementary Figure S23. Forest plot for mixed treatment comparison pooled estimates for the risk of therapy discontinuation of SGLT2is compared to non-SGLT2i group amongst various age groups.

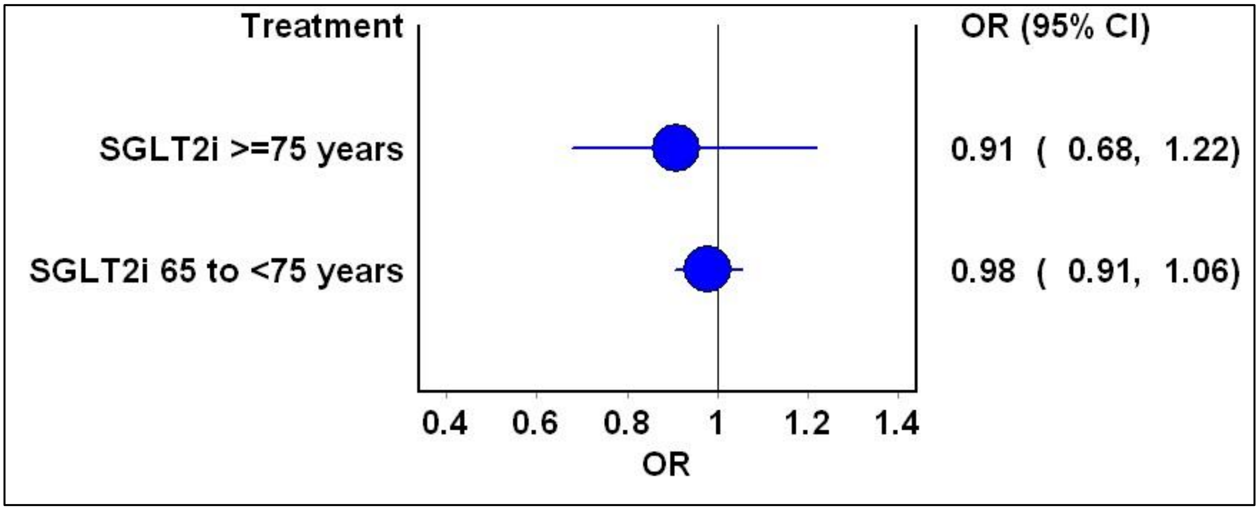

Electronic Supplementary Figure S24. Forest plot for mixed treatment comparison pooled estimates for the risk of fracture with SGLT2is compared to non-SGLT2i group amongst various age groups.

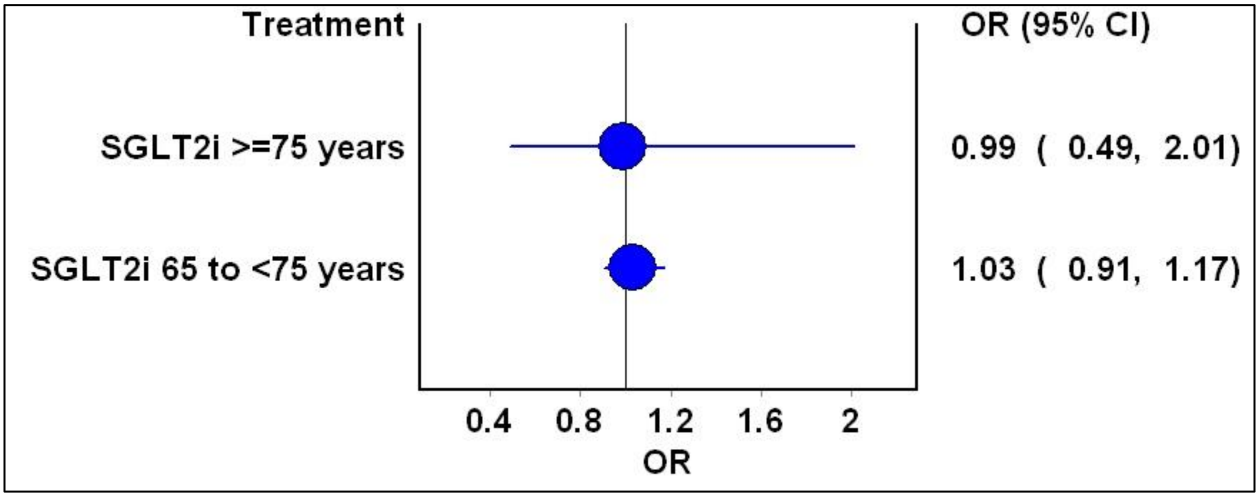

Electronic Supplementary Figure S25. Forest plot for mixed treatment comparison pooled estimates for the risk of malignancies with SGLT2is compared to non-SGLT2i group amongst various age groups.

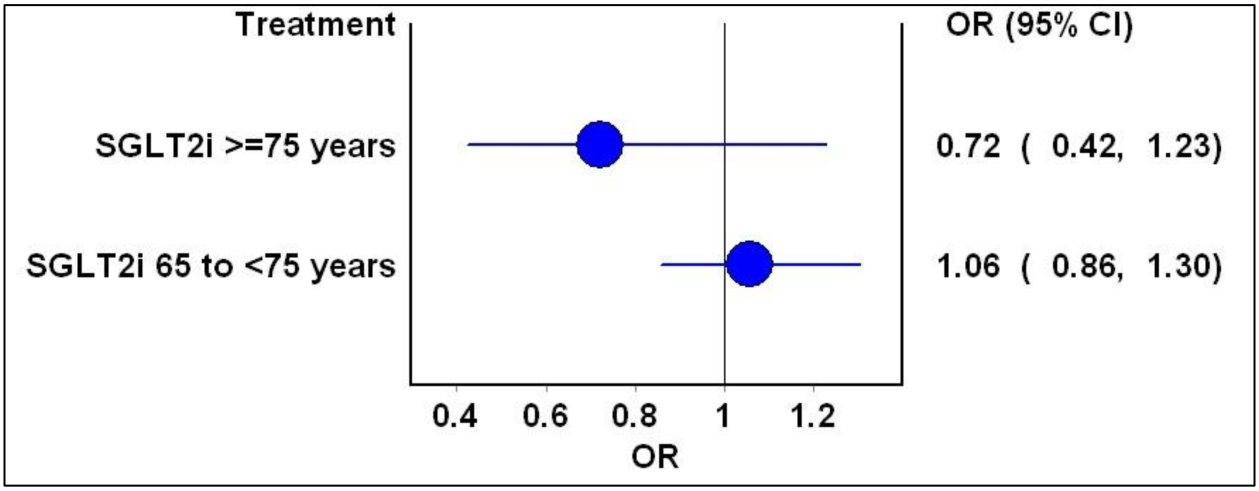

Electronic Supplementary Figure S26. Forest plot for mixed treatment comparison pooled estimates for the risk of stroke with SGLT2is compared to non-SGLT2i group amongst various age groups.

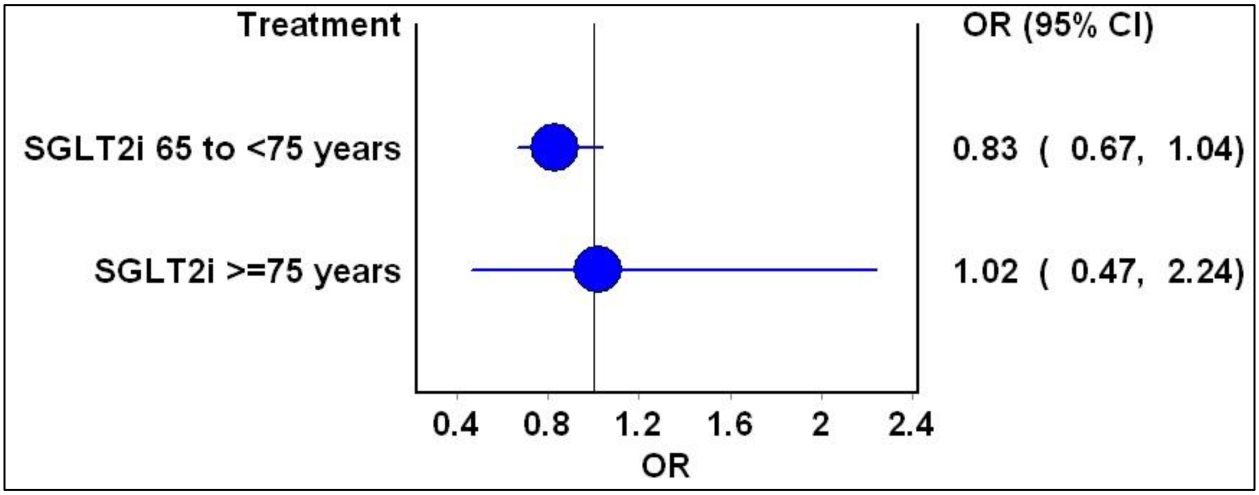

Electronic Supplementary Figure S27. Forest plot for mixed treatment comparison pooled estimates for the risk of urinary tract infection with SGLT2is compared to non-SGLT2i group amongst various age groups.

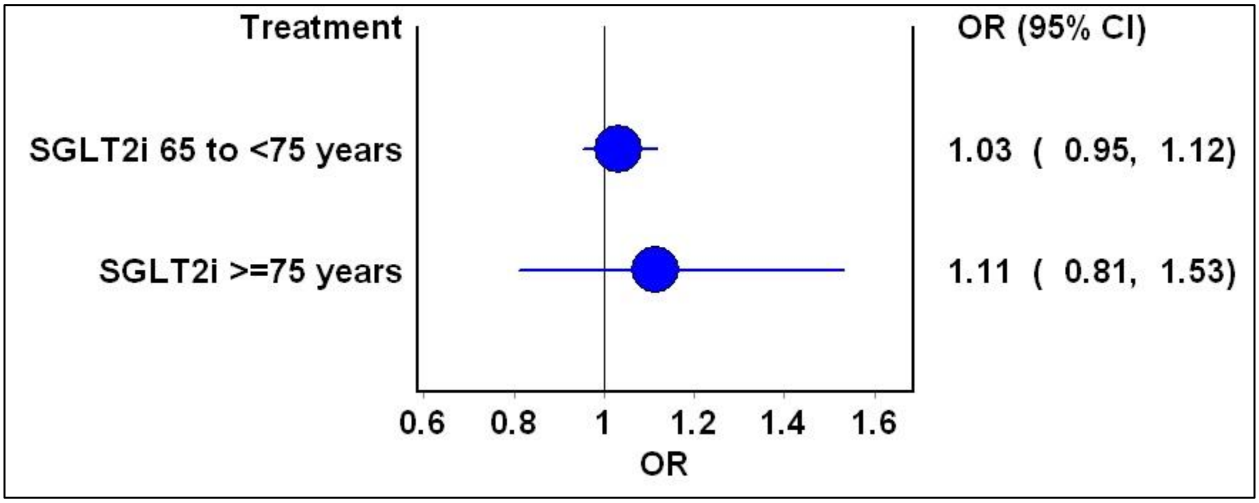

Electronic Supplementary Figure S28. Network plot for mixed treatment comparison pooled estimates for the individual SGLT2is compared to non-SGLT2i group across outcomes.

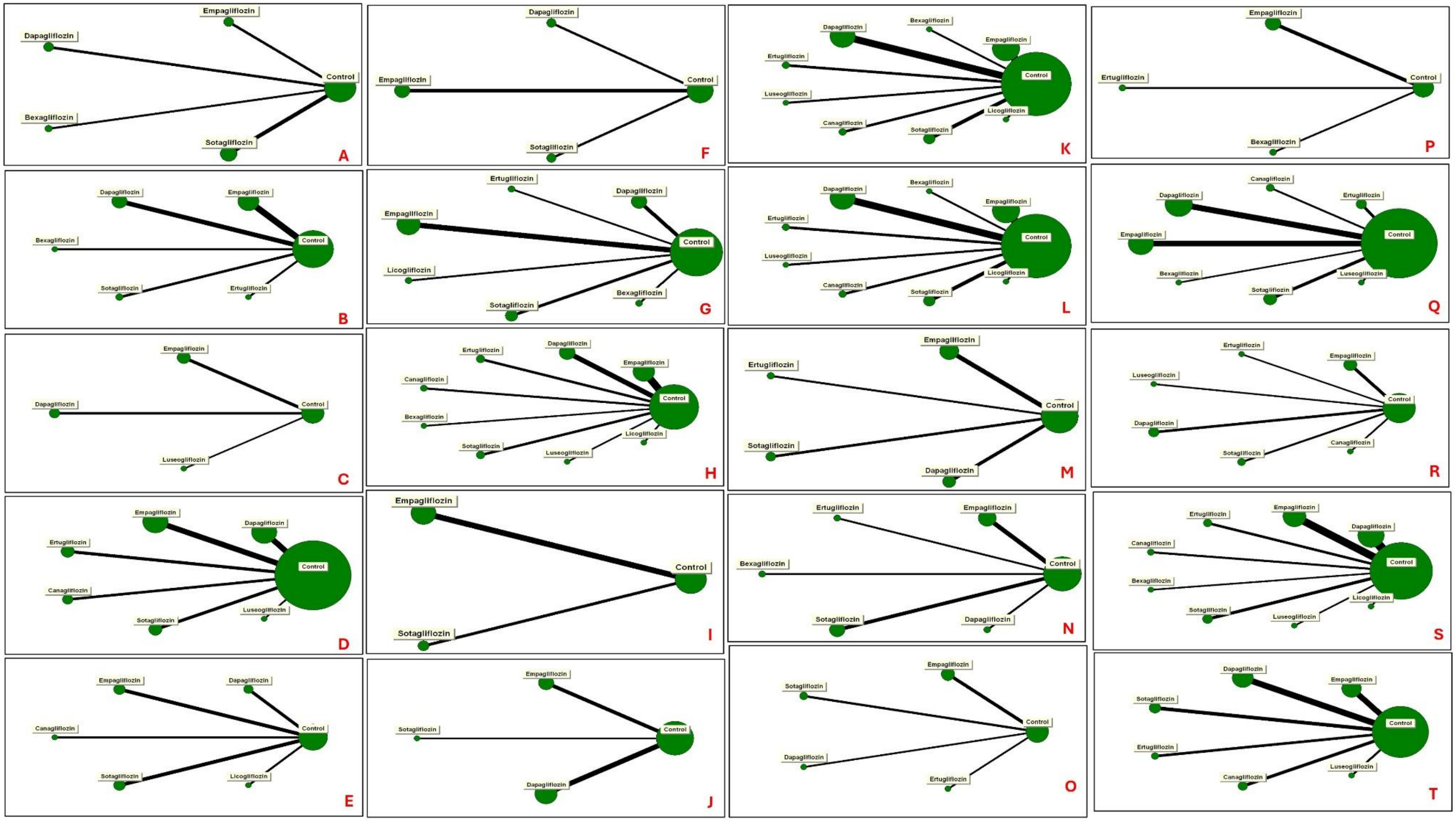

A- Amputation; B-ARF; C-Constipation; D-Death; E-Diarrhea; F-DKA; G-Fracture; I-hep dysfunction; J-Hyperkalemia; K-Hyperkalemia; L-hypoglycemia; M-Hypotension; N-Malignancies; O-MI; P-Polyuria; Q-SAE; R-Stroke; S-UTI; and T-Volume depletion.

Electronic Supplementary Figure S29. Forest plot for mixed treatment comparison pooled estimates for the risk of constipation with individual SGLT2is compared to non-SGLT2i group.

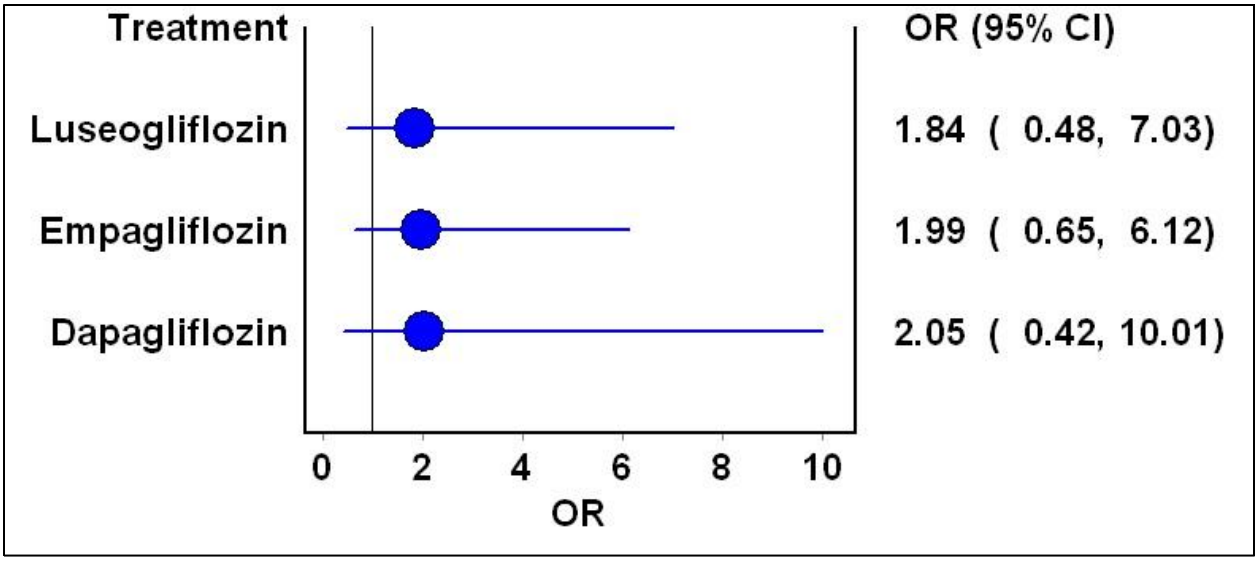

Electronic Supplementary Figure S30. Forest plot for mixed treatment comparison pooled estimates for the risk of hypoglycemia with individual SGLT2is compared to non-SGLT2i group.

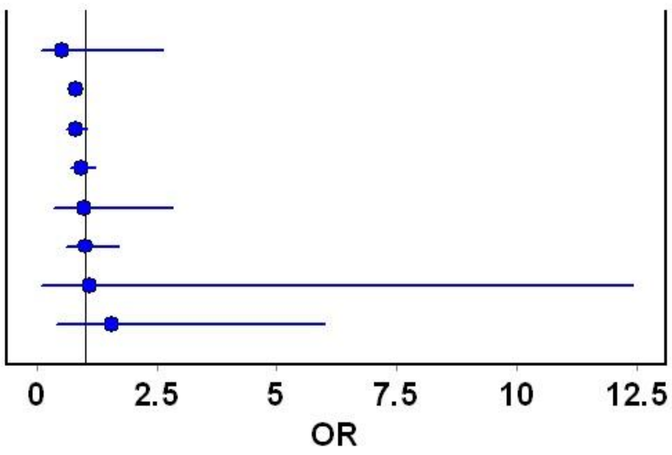

Treatment	OR (95% CI)
Luseogliflozin	0.51 (0.10, 2.63)
Empagliflozin	0.79 (0.71, 0.89)
Dapagliflozin	0.80 (0.62, 1.04)
Sotagliflozin	0.92 (0.70, 1.22)
Canagliflozin	0.97 (0.33, 2.81)
Bexagliflozin	1.02 (0.61, 1.70)
Licogliflozin	1.08 (0.09, 12.43)
Ertugliflozin	1.57 (0.41, 5.99)

Electronic Supplementary Figure S31. Forest plot for mixed treatment comparison pooled estimates for the risk of hyperkalemia with individual SGLT2is compared to non-SGLT2i group.

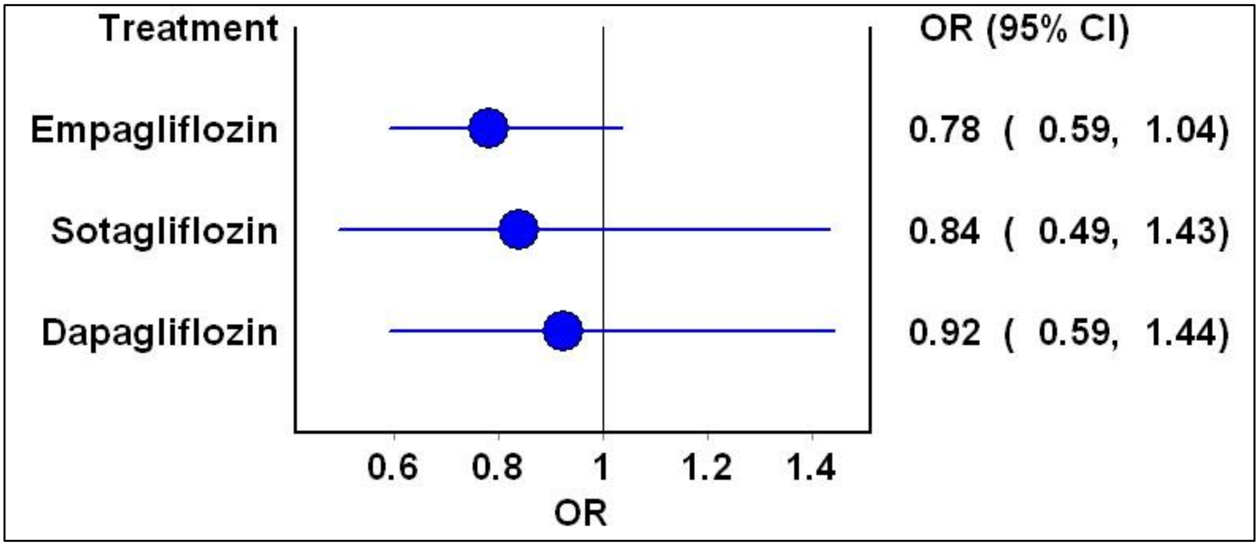

Electronic Supplementary Figure 32. Forest plot for mixed treatment comparison pooled estimates for the risk of fracture with individual SGLT2is compared to non-SGLT2i group.

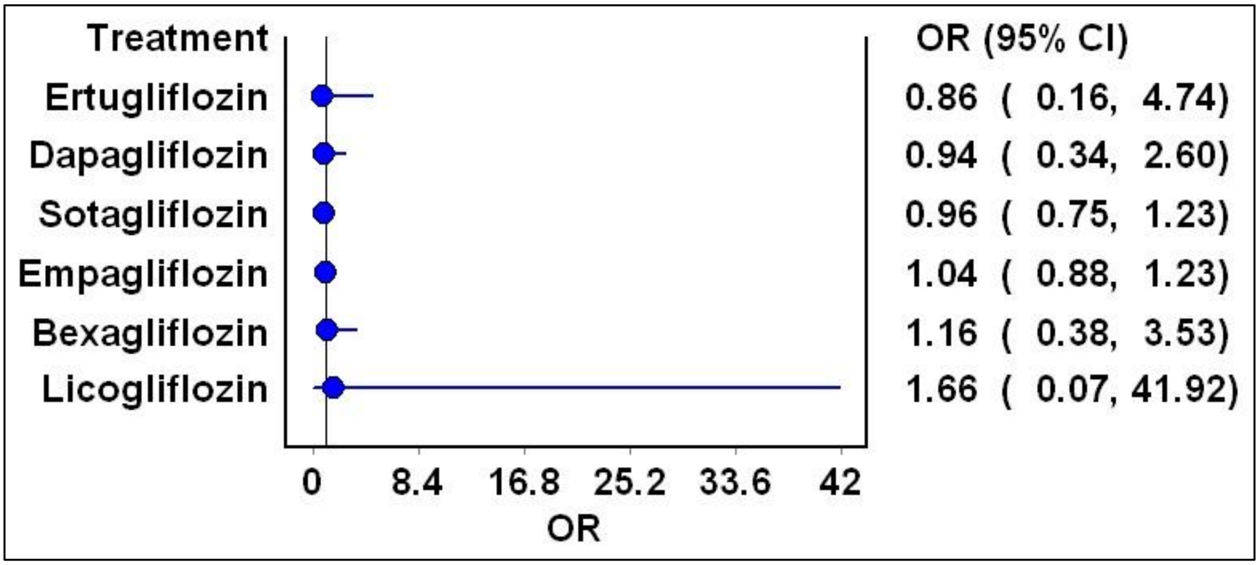

Electronic Supplementary Figure S33. Forest plot for mixed treatment comparison pooled estimates for the risk of hepatic dysfunction with individual SGLT2is compared to non-SGLT2i group.

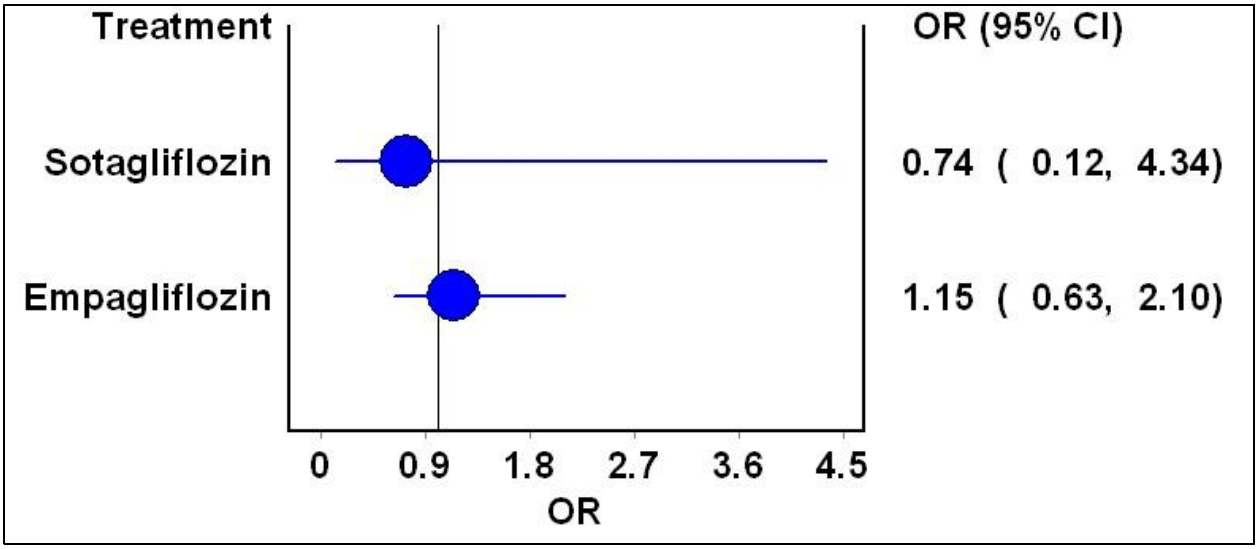

Electronic Supplementary Figure S34. Forest plot for mixed treatment comparison pooled estimates for the risk of amputation with individual SGLT2is compared to non-SGLT2i group.

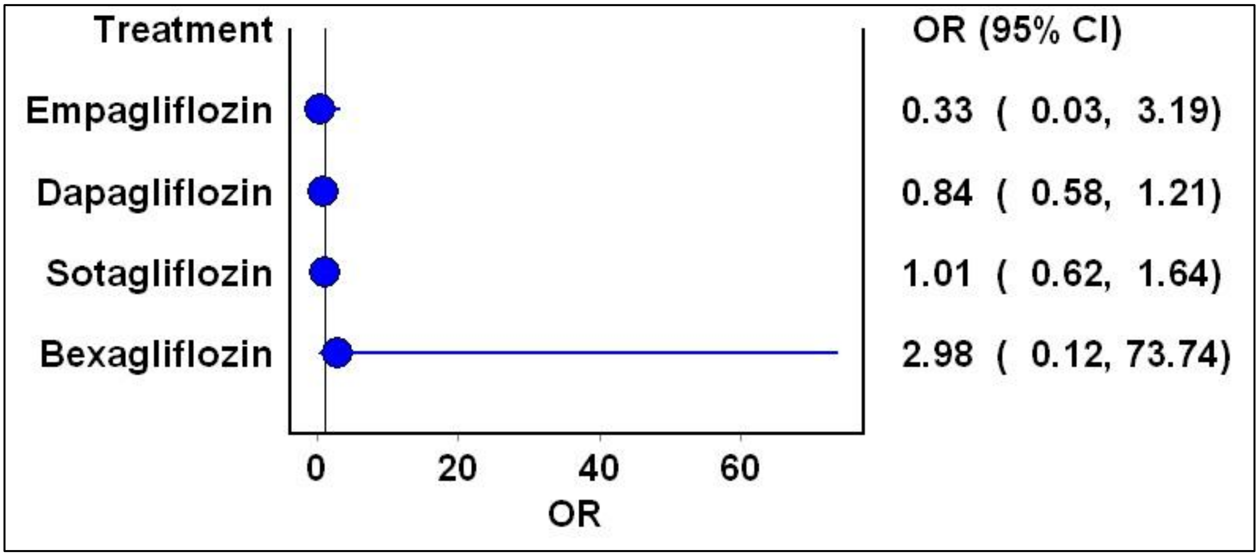

Electronic Supplementary Figure S35. Forest plot for mixed treatment comparison pooled estimates for the risk of DKA with individual SGLT2is compared to non-SGLT2i group.

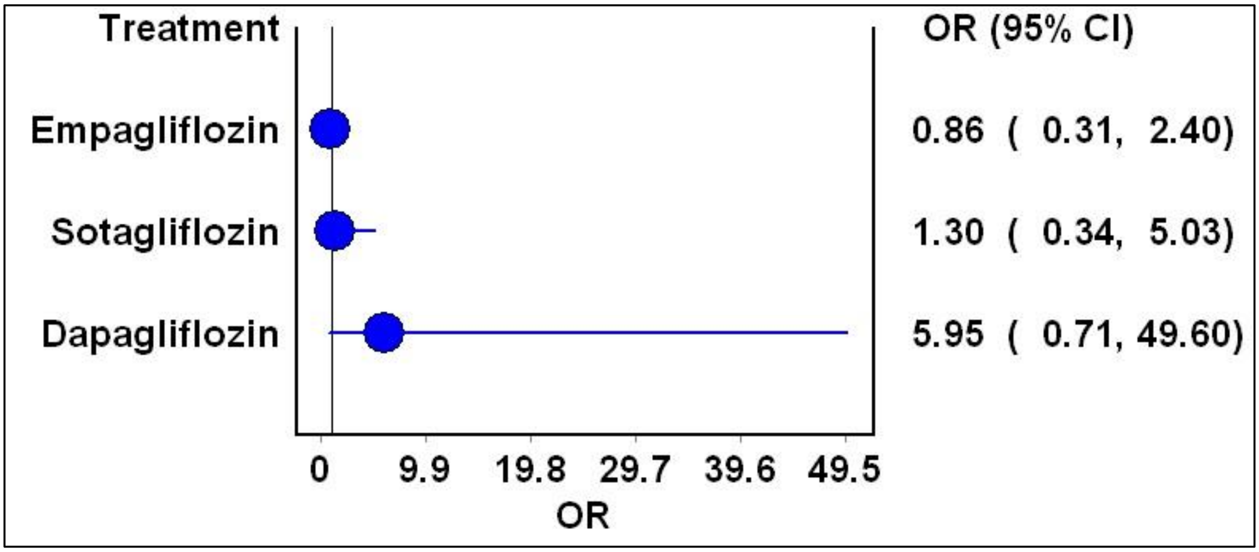

Electronic Supplementary Figure S36. Forest plot for mixed treatment comparison pooled estimates for the risk of malignancies with individual SGLT2is compared to non-SGLT2i group.

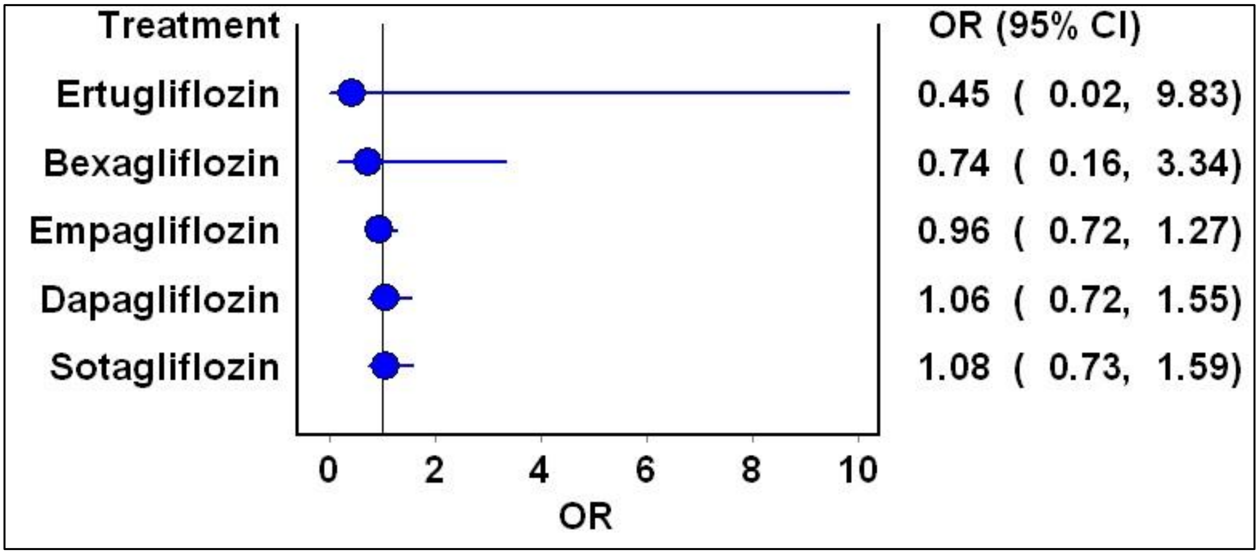

Electronic Supplementary Figure S37. Bootstrap histogram for the pooled estimates for the risk of volume depletion with SGLT2is compared to non-SGLT2i group.

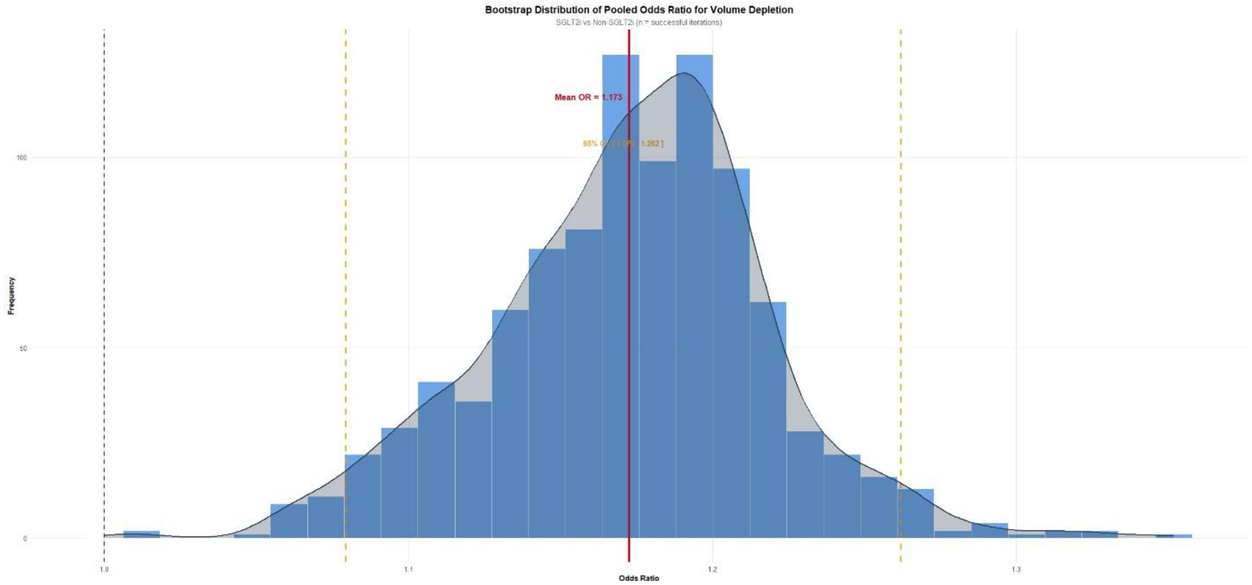

Electronic Supplementary Figure S38. Bootstrap histogram for the pooled estimates for the risk of mortality with SGLT2is compared to non-SGLT2i group.

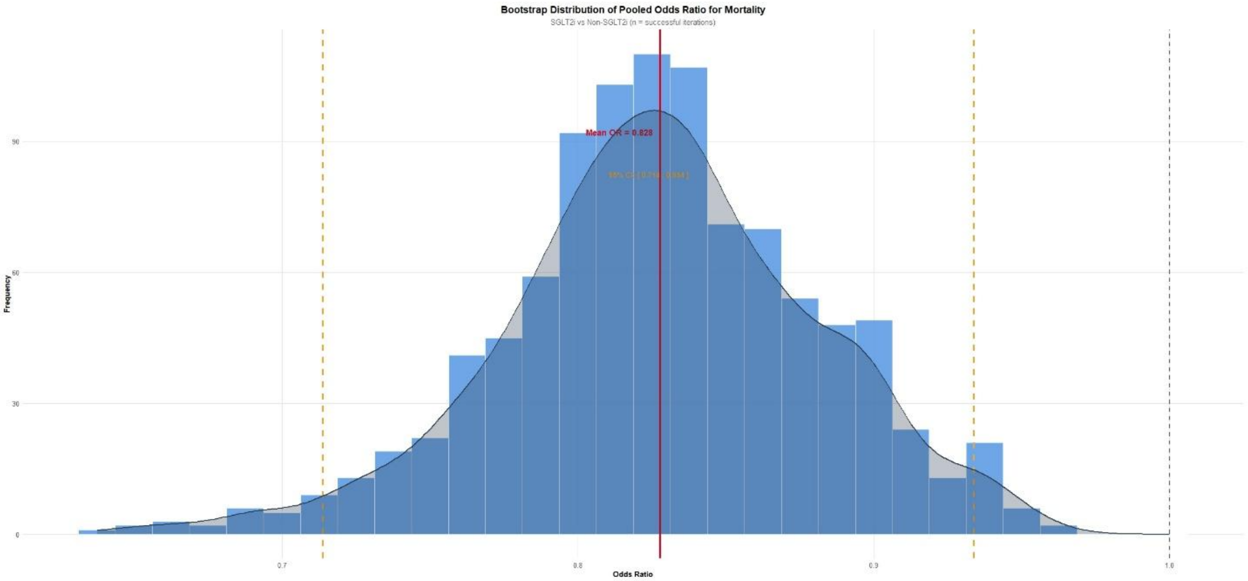

Electronic Supplementary Figure S39. Bootstrap histogram for the pooled estimates for the risk of polyuria with SGLT2is compared to non-SGLT2i group.

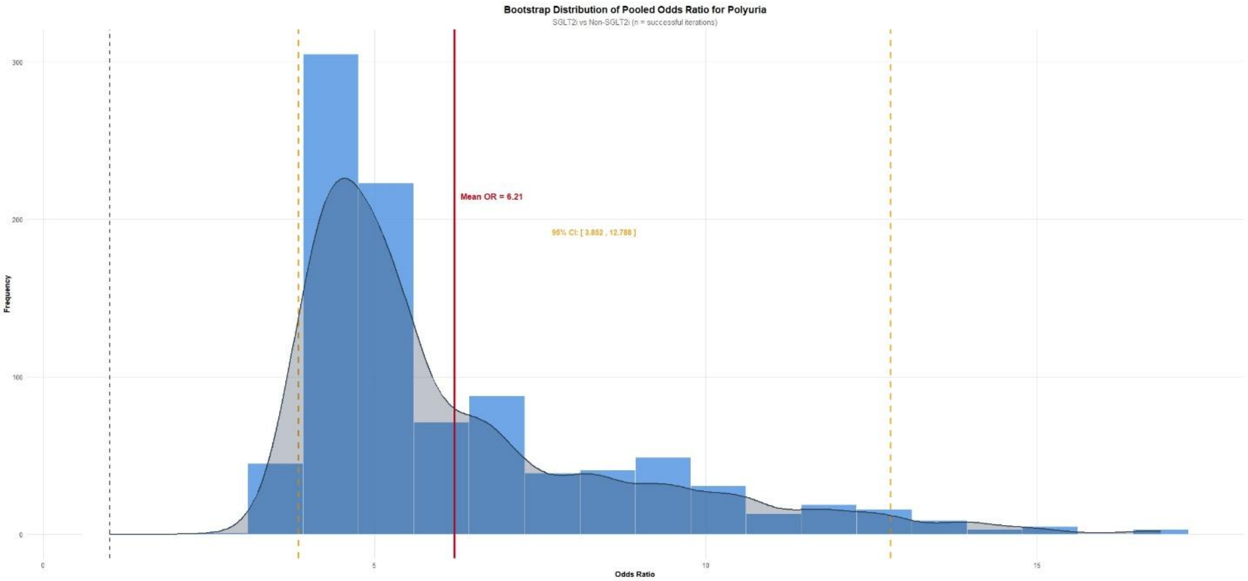

Electronic Supplementary Figure S40. Bootstrap histogram for the pooled estimates for the risk of serious adverse events with SGLT2is compared to non-SGLT2i group.

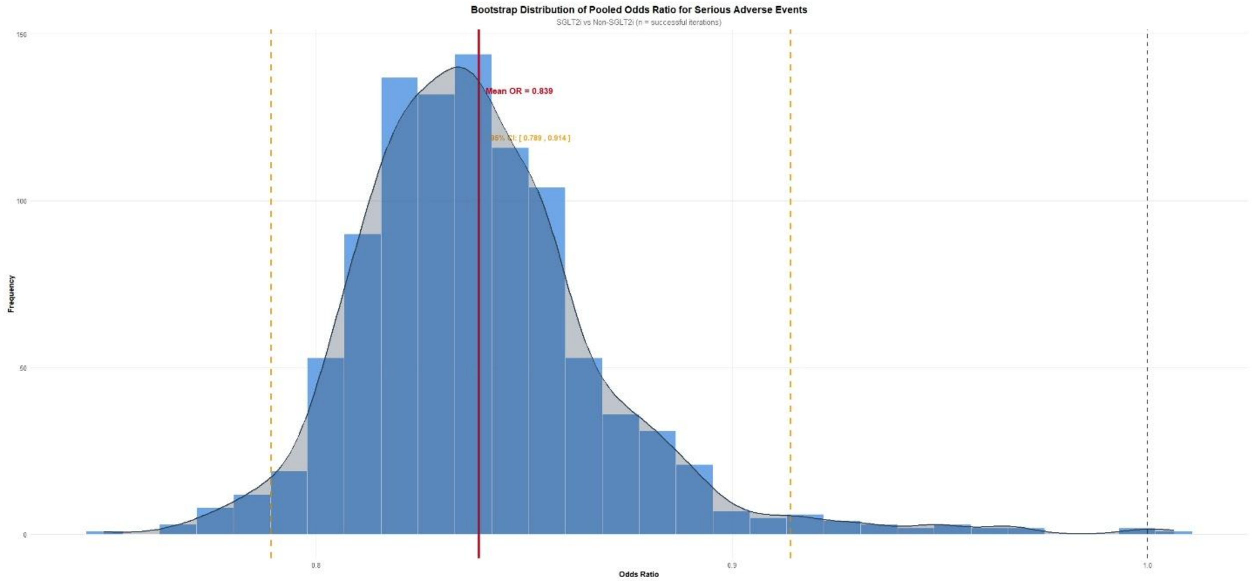

Electronic Supplementary Figure S41. Bootstrap histogram for the pooled estimates for the risk of diarrhea with SGLT2is compared to non-SGLT2i group.

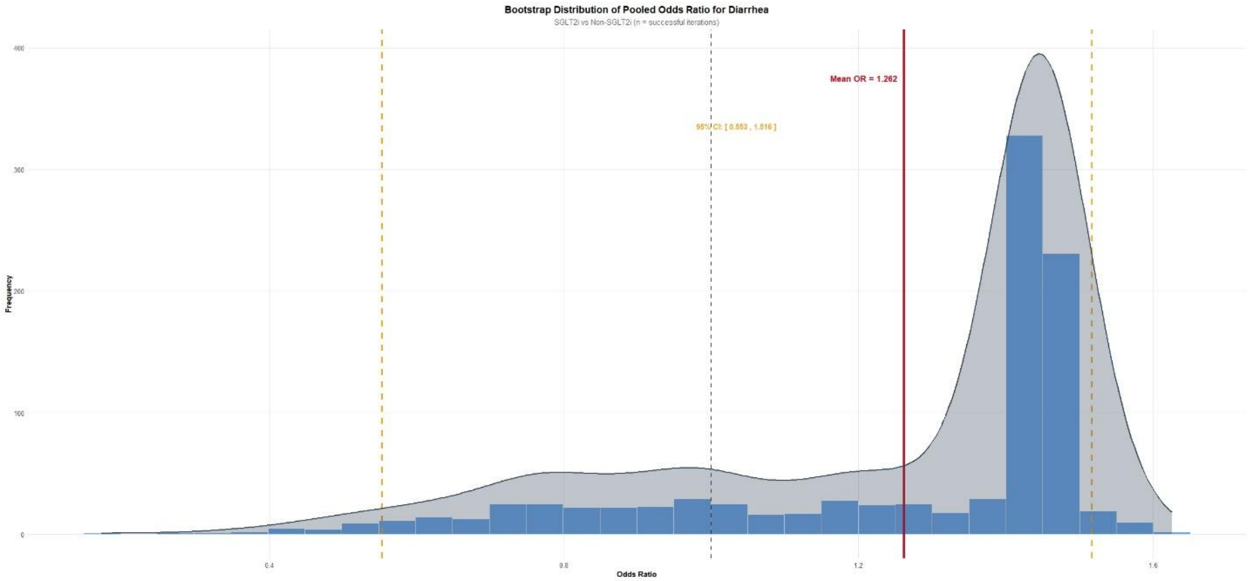

Electronic Supplementary Figure S42. Bootstrap histogram for the pooled estimates for the risk of myocardial infarction with SGLT2is compared to non-SGLT2i group.

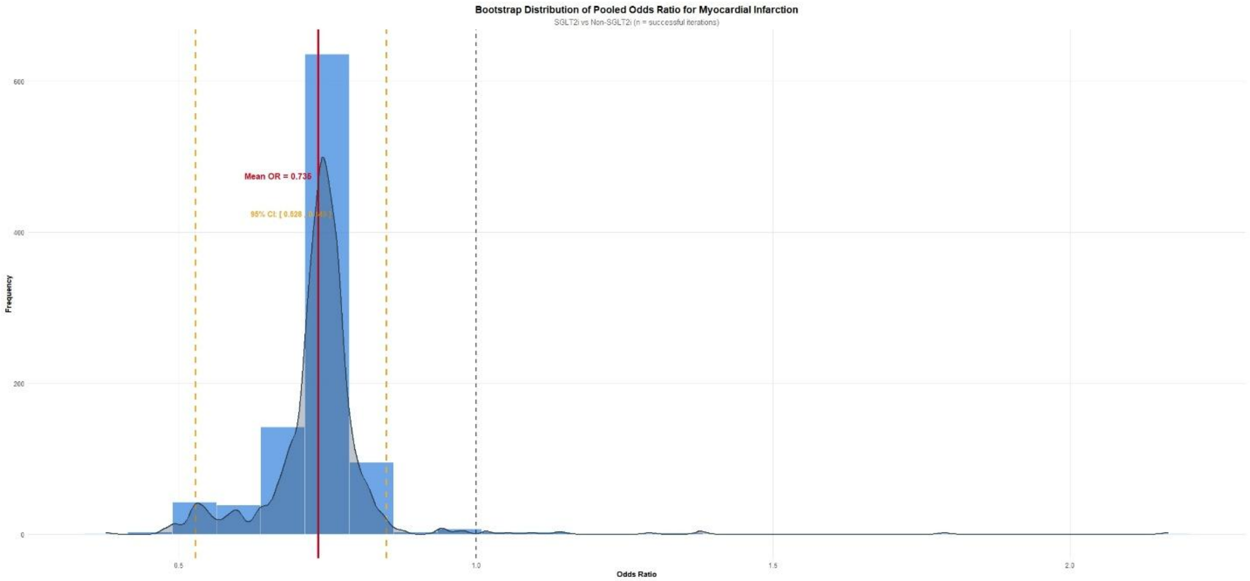

Electronic Supplementary Figure S43. Bootstrap histogram for the pooled estimates for the risk of genital infections with SGLT2is compared to non-SGLT2i group.

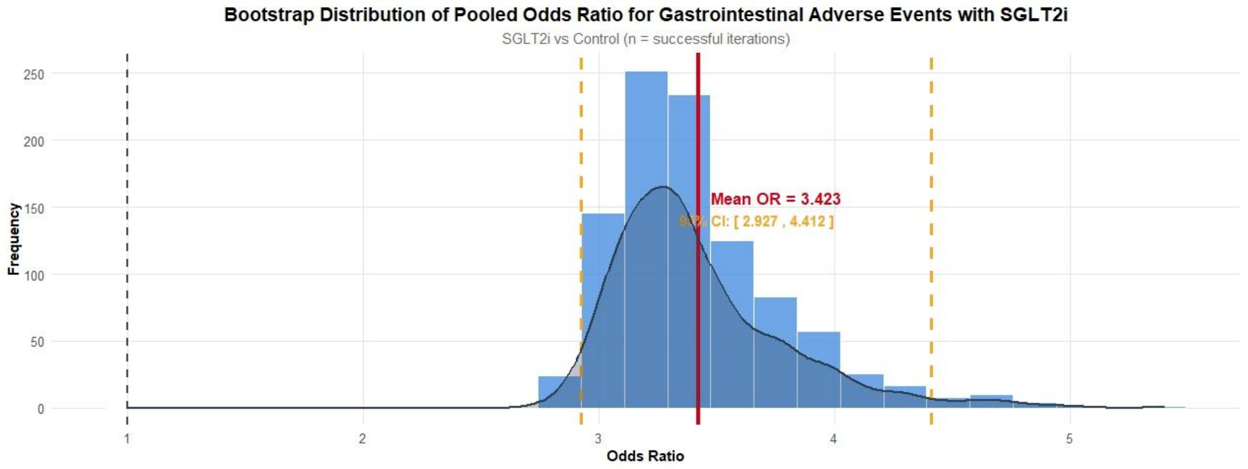

Electronic Supplementary Figure S44. Bootstrap histogram for the pooled estimates for the risk of ARF with SGLT2is compared to non-SGLT2i group.

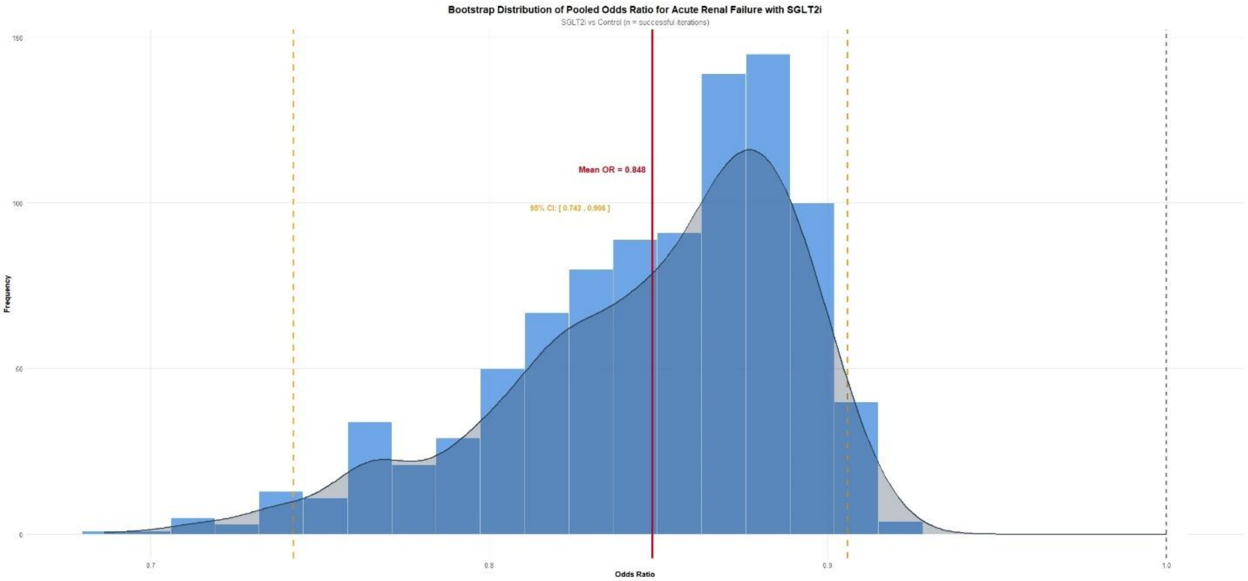

Electronic Supplementary Figure S45. TSA plot for the risk of ARF with SGLT2is compared to non-SGLT2i group.

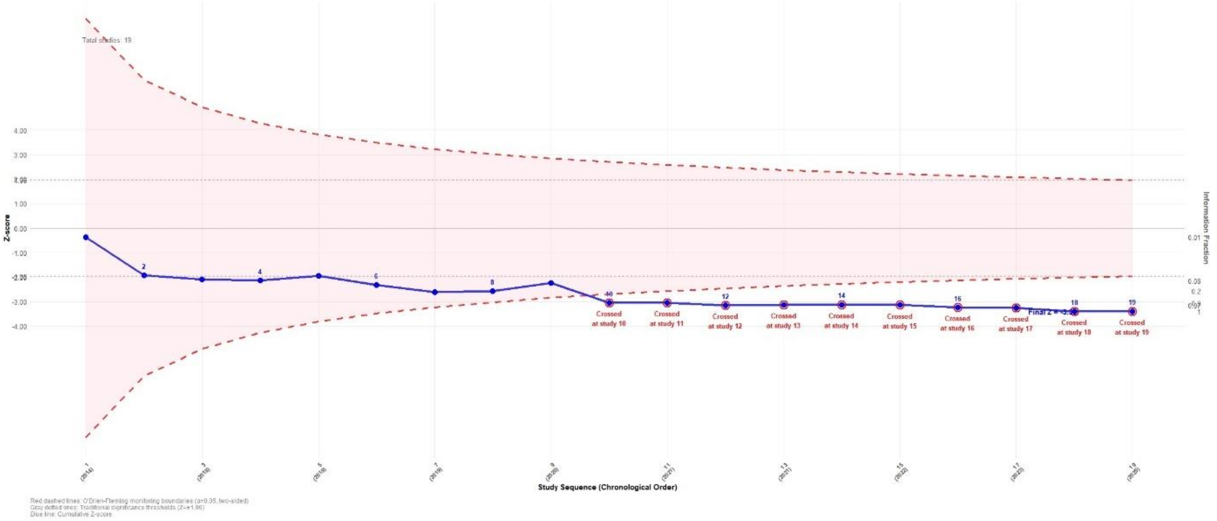

Electronic Supplementary Figure S46. TSA plot for the risk of mortality with SGLT2is compared to non-SGLT2i group.

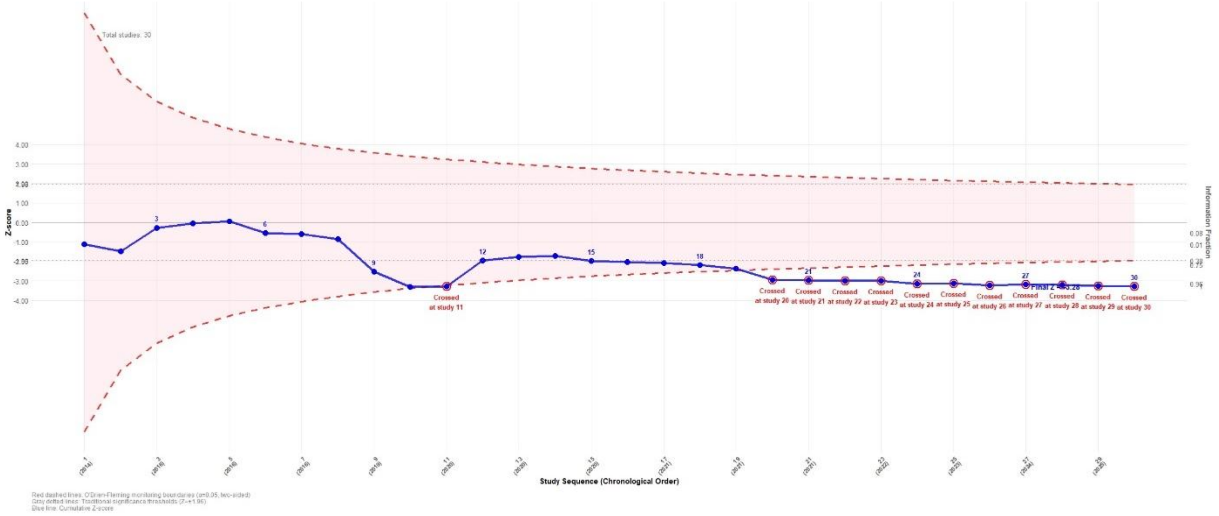

Electronic Supplementary Figure S47. TSA plot for the risk of SAE with SGLT2is compared to non-SGLT2i group.

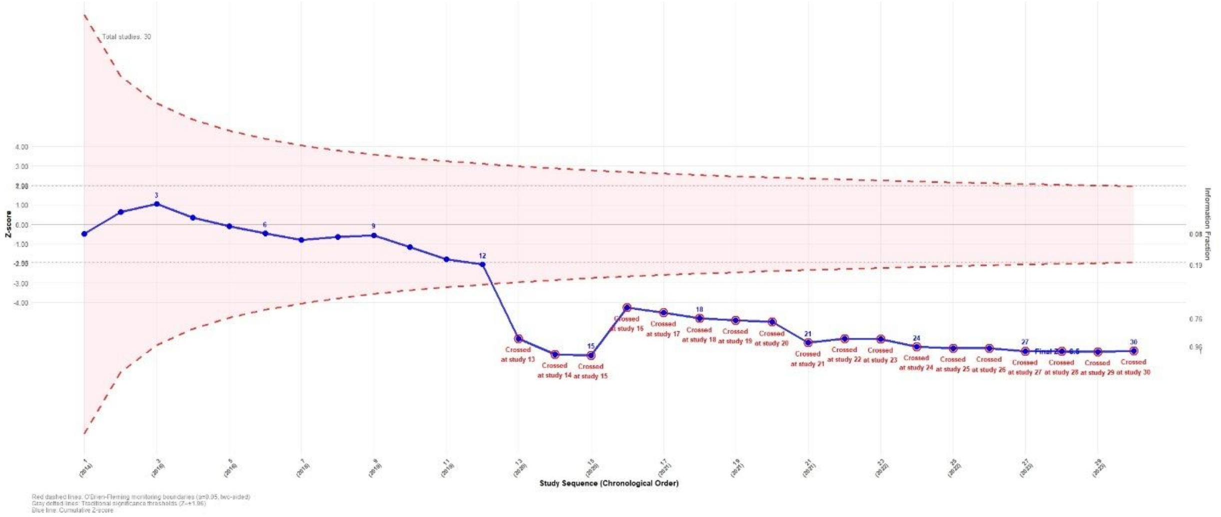

Electronic Supplementary Figure S48. TSA plot for the risk of genital infections with SGLT2is compared to non-SGLT2i group.

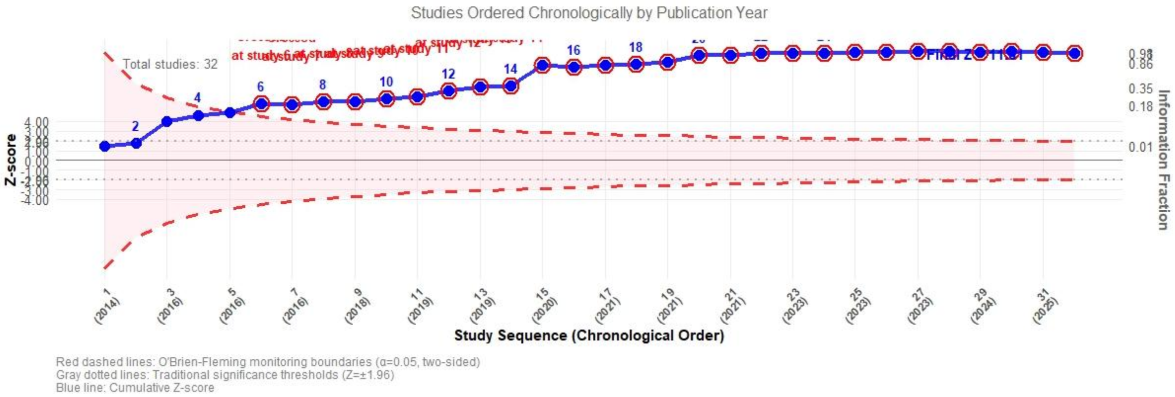

Electronic Supplementary Figure S49. TSA plot for the risk of polyuria with SGLT2is compared to non-SGLT2i group.

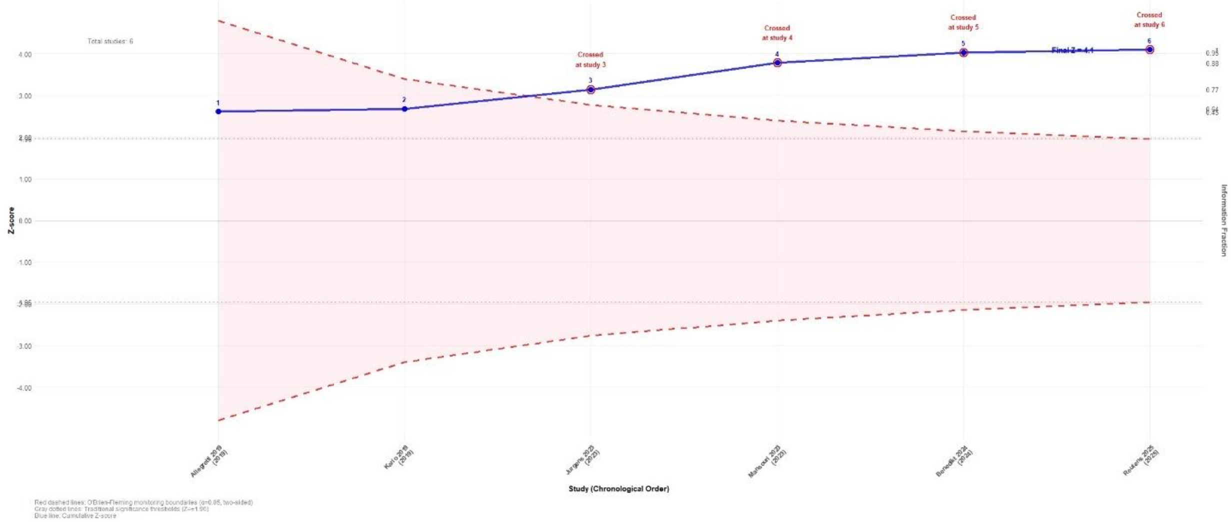

Electronic Supplementary Figure S50. TSA plot for the risk of myocardial infarction with SGLT2is compared to non-SGLT2i group.

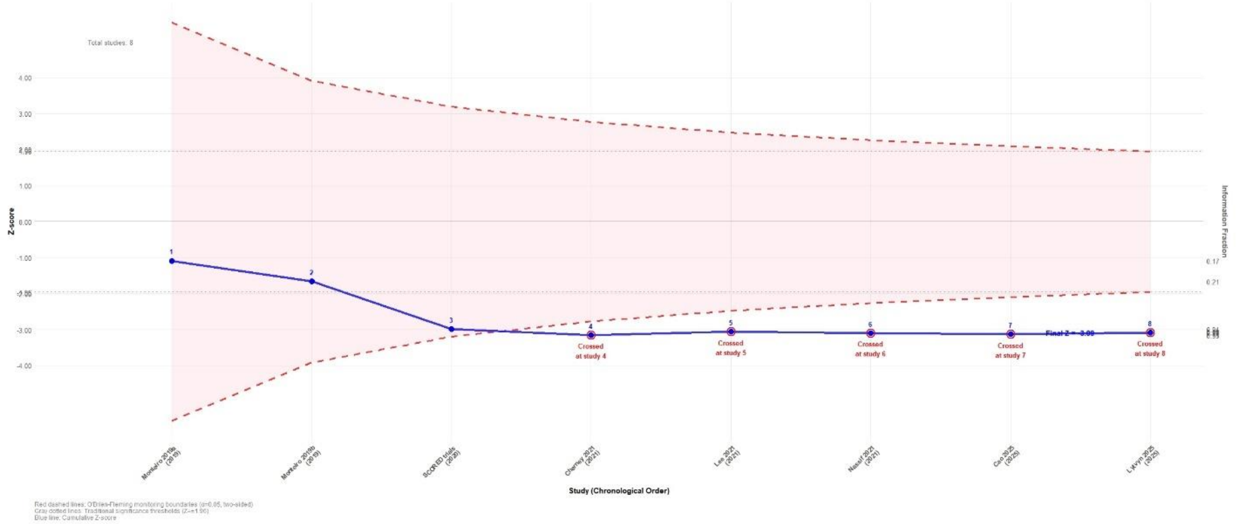

Electronic Supplementary Figure S51. TSA plot for the risk of diarrhea with SGLT2is compared to non-SGLT2i group.

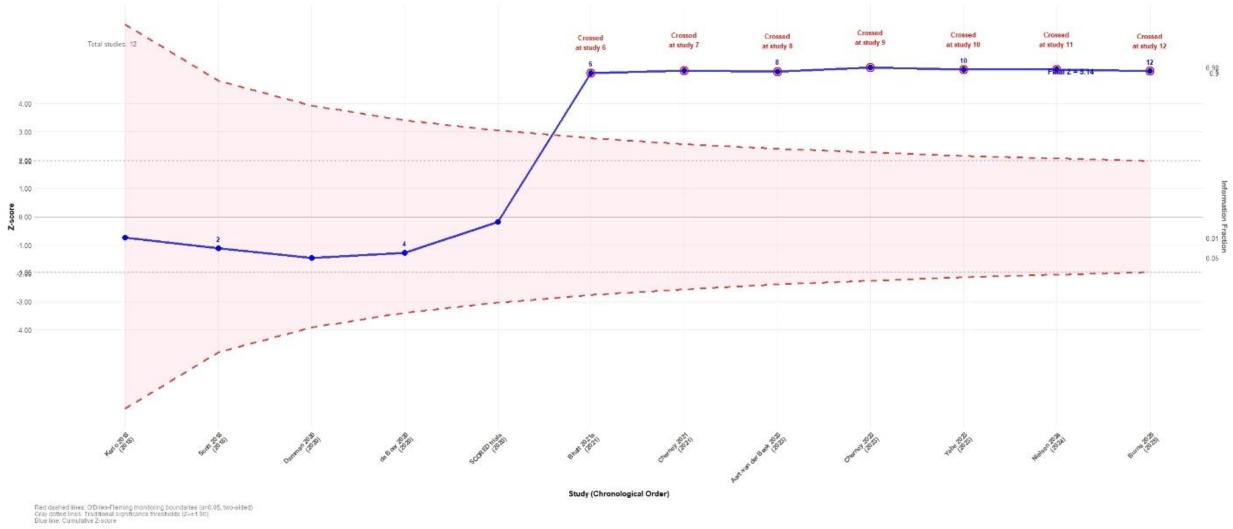

Electronic Supplementary Figure S52. Funnel plot for the risk of diarrhea with SGLT2is compared to non-SGLT2i group.

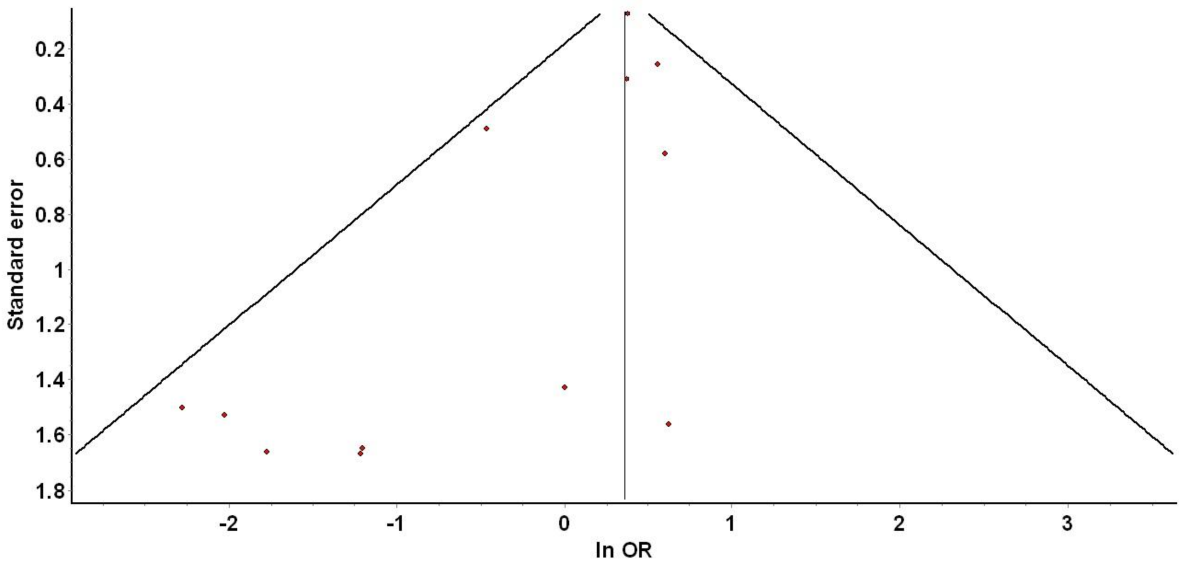

Electronic Supplementary Figure S53. Funnel plot for the risk of ARF with SGLT2is compared to non-SGLT2i group.

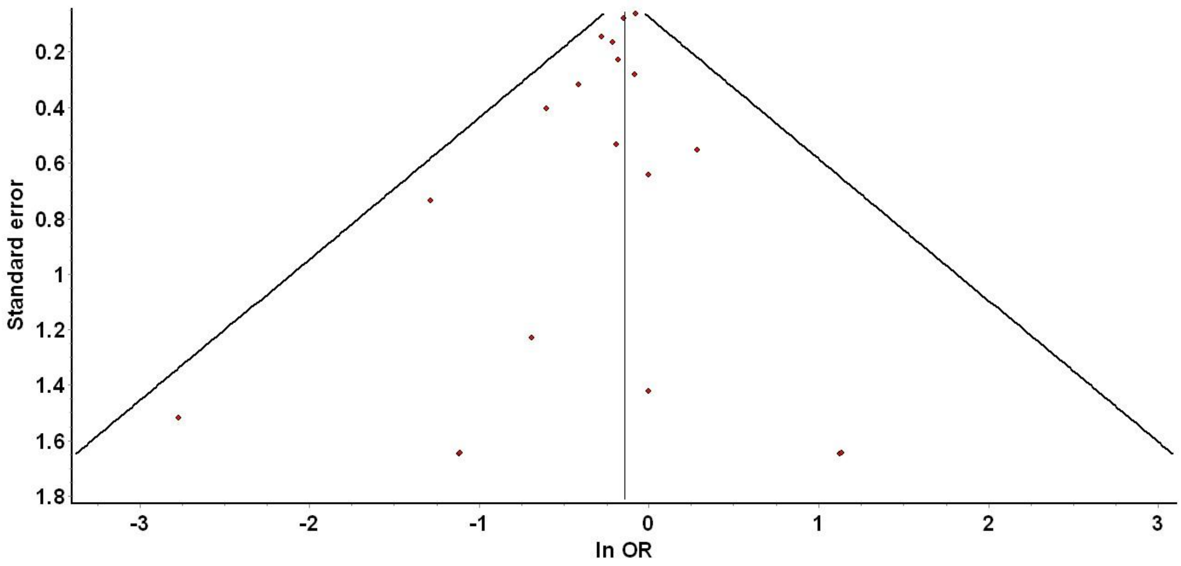

Electronic Supplementary Figure S54. Funnel plot for the risk of mortality with SGLT2is compared to non-SGLT2i group.

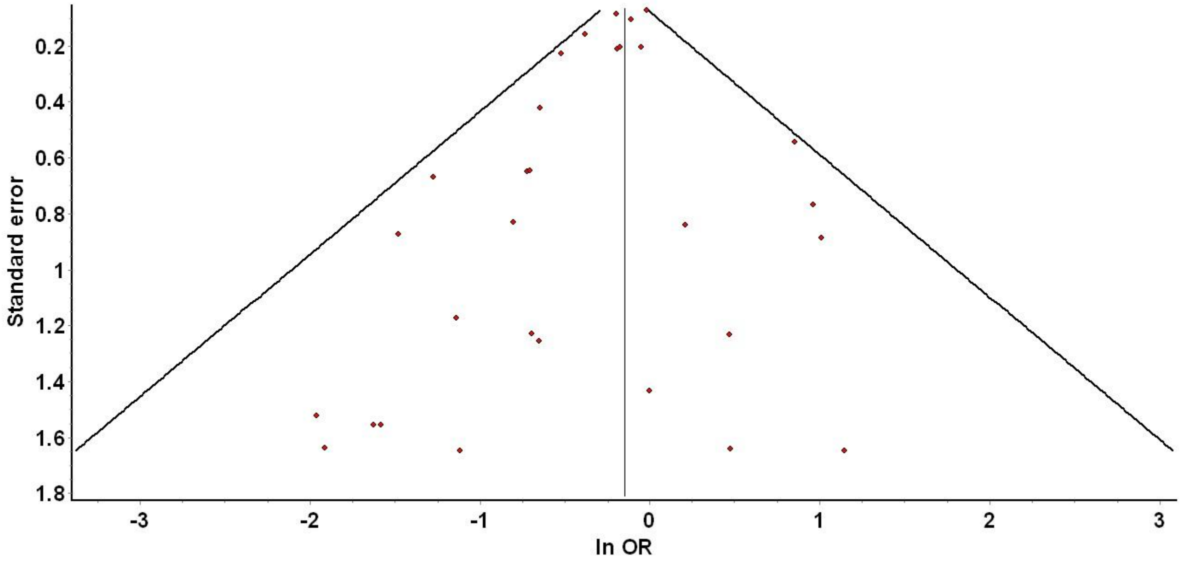

Electronic Supplementary Figure S55. Funnel plot for the risk of SAE with SGLT2is compared to non-SGLT2i group.

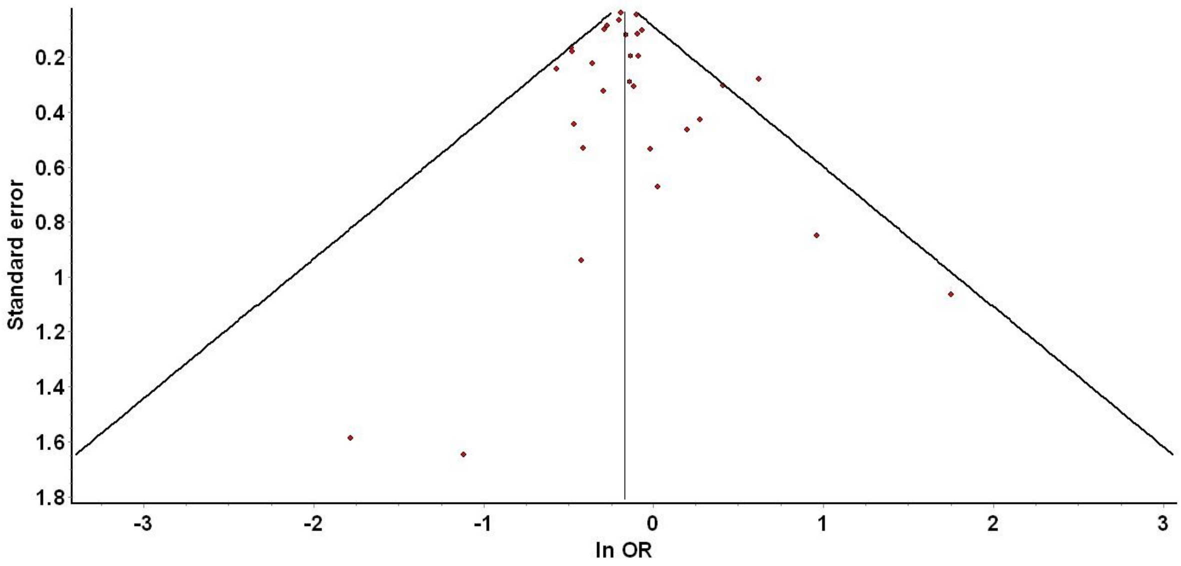

Electronic Supplementary Figure S56. Funnel plot for the risk of polyuria with SGLT2is compared to non-SGLT2i group.

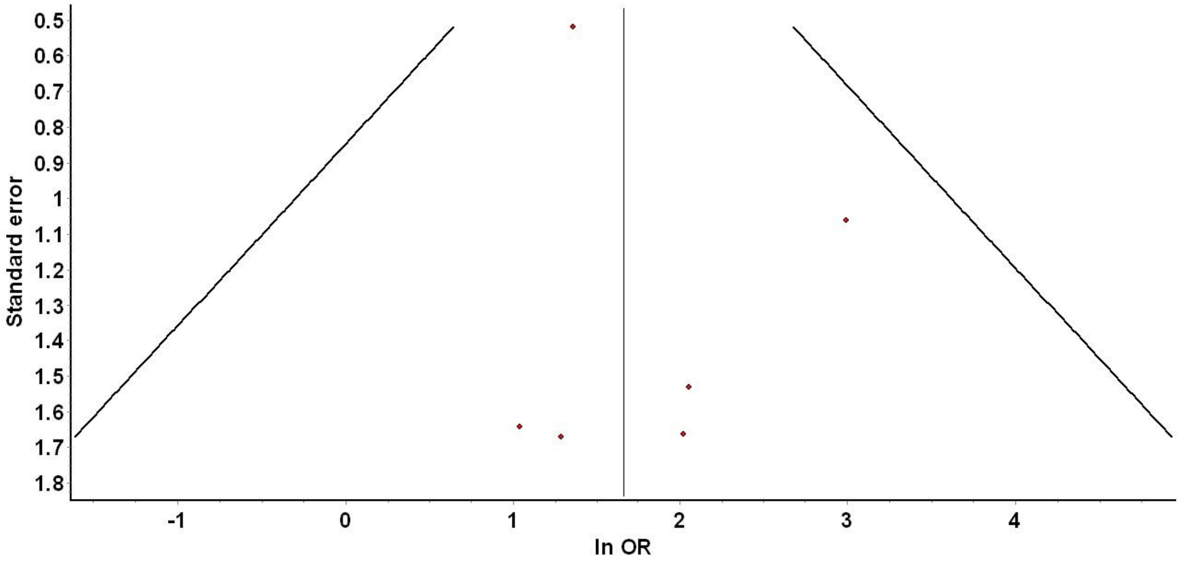

Electronic Supplementary Figure S57. Funnel plot for the risk of volume depletion with SGLT2is compared to non-SGLT2i group.

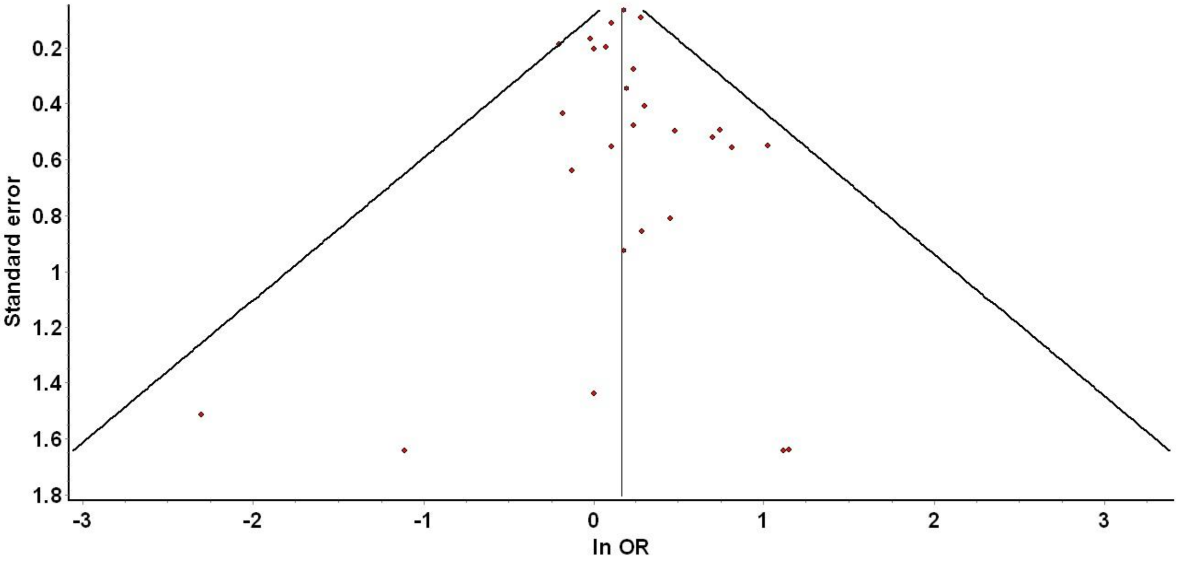

Electronic Supplementary Figure S58. Funnel plot for the risk of myocardial infarction with SGLT2is compared to non-SGLT2i group.

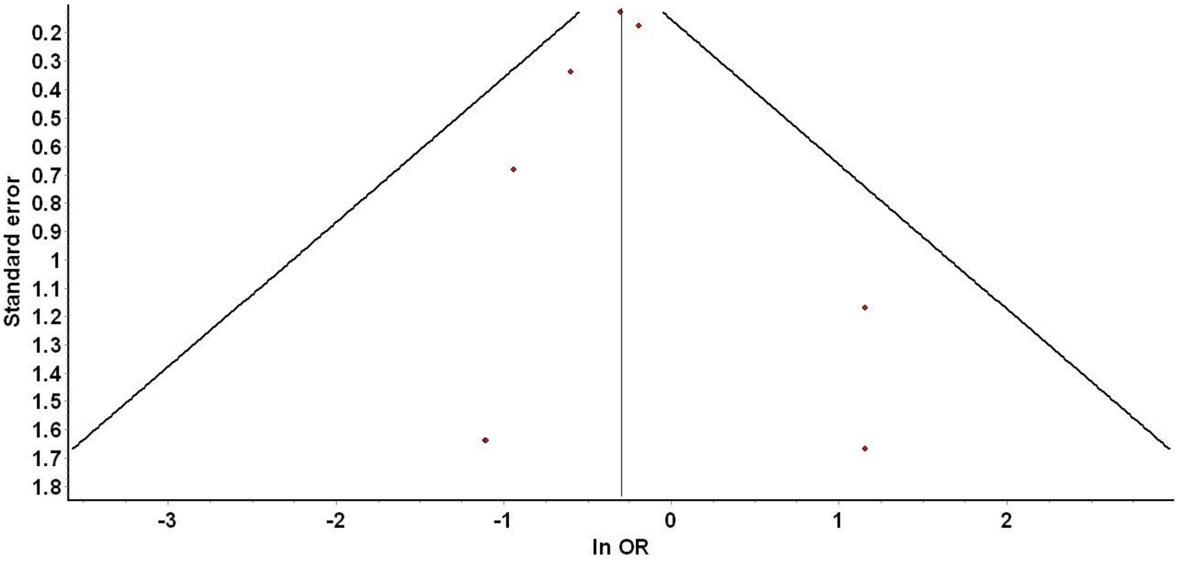

Electronic Supplementary Figure S59. Funnel plot for the risk of genital infections with SGLT2is compared to non-SGLT2i group.

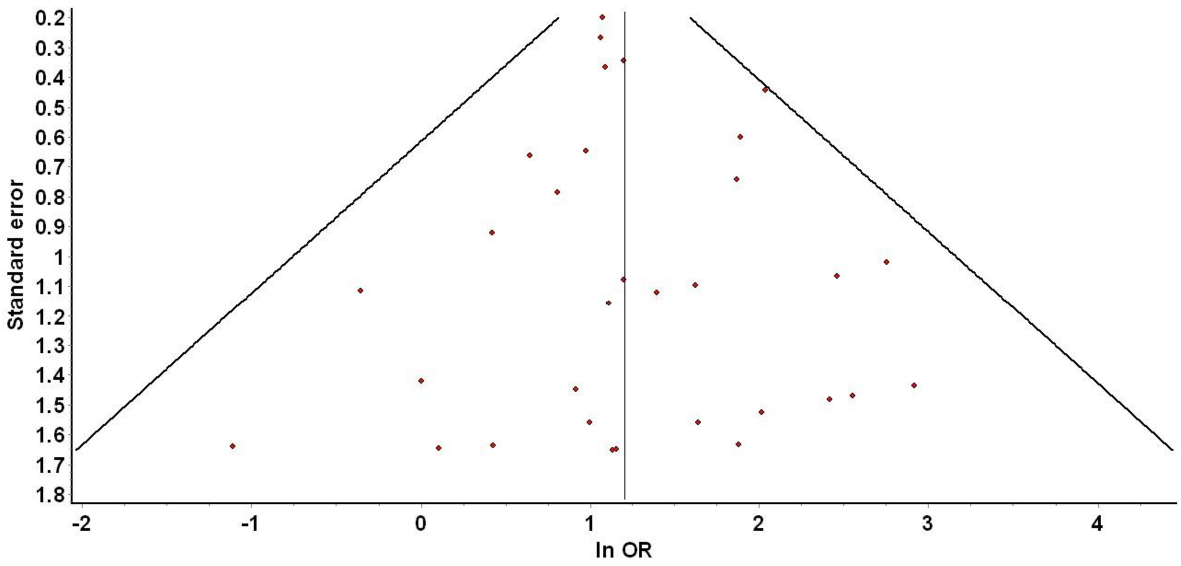

Supplement: Supplementary file 1 [file medsci-14-00153-s001.zip › medsci-4134613-supplementary.pdf]
